# Structural and Mechanistic Analysis of the Choline Sulfatase from *Sinorhizobium melliloti*: A Class I Sulfatase Specific for an Alkyl Sulfate Ester

**DOI:** 10.1016/j.jmb.2018.02.010

**Published:** 2018-03-30

**Authors:** Bert van Loo, Markus Schober, Eugene Valkov, Magdalena Heberlein, Erich Bornberg-Bauer, Kurt Faber, Marko Hyvönen, Florian Hollfelder

**Affiliations:** 1 Department of Biochemistry, University of Cambridge, 80 Tennis Court Road, Cambridge CB2 1GA, United Kingdom; 2 Institute for Evolution and Biodiversity, University of Münster, Hüfferstrasse 1, D-48149 Münster, Germany; 3 Department of Chemistry, Organic & Bioorganic Chemistry, University of Graz, Heinrichstrasse 28, A-8010 Graz, Austria

**Keywords:** arylsulfatase, alkaline phosphatase superfamily, phosphatase, catalytic promiscuity, oligomerization, AS, arylsulfatase, *Sm*CS, *Sinorhizobium meliloti* choline sulfatase, CS, choline sulfatase, AP, alkaline phosphatase, PAS, *Pseudomonas aeruginosa* AS, PMH, phosphonate monoester hydrolase, fGly, formyglycine, FGE, fGly-generating enzyme, *Sp*AS1, *Silicibacter pomeroyi* AS1, *Rl*PMH, *Rhizobium leguminosarum* PMH

## Abstract

Hydrolysis of organic sulfate esters proceeds by two distinct mechanisms, water attacking at either sulfur (S–O bond cleavage) or carbon (C–O bond cleavage). In primary and secondary alkyl sulfates, attack at carbon is favored, whereas in aromatic sulfates and sulfated sugars, attack at sulfur is preferred. This mechanistic distinction is mirrored in the classification of enzymes that catalyze sulfate ester hydrolysis: arylsulfatases (ASs) catalyze S–O cleavage in sulfate sugars and arylsulfates, and alkyl sulfatases break the C–O bond of alkyl sulfates. *Sinorhizobium meliloti* choline sulfatase (*Sm*CS) efficiently catalyzes the hydrolysis of *alkyl* sulfate choline-*O*-sulfate (*k*_cat_/*K*_M_ = 4.8 × 10^3^ s^− 1^ M^− 1^) as well as *aryl*sulfate 4-nitrophenyl sulfate (*k*_cat_/*K*_M_ = 12 s^− 1^ M^− 1^). Its 2.8-Å resolution X-ray structure shows a buried, largely hydrophobic active site in which a conserved glutamate (Glu386) plays a role in recognition of the quaternary ammonium group of the choline substrate. *Sm*CS structurally resembles members of the alkaline phosphatase superfamily, being most closely related to dimeric ASs and tetrameric phosphonate monoester hydrolases. Although > 70% of the amino acids between protomers align structurally (RMSDs 1.79–1.99 Å), the oligomeric structures show distinctly different packing and protomer–protomer interfaces. The latter also play an important role in active site formation. Mutagenesis of the conserved active site residues typical for ASs, H_2_^18^O-labeling studies and the observation of catalytically promiscuous behavior toward phosphoesters confirm the close relation to alkaline phosphatase superfamily members and suggest that *Sm*CS is an AS that catalyzes S–O cleavage in *alkyl* sulfate esters with extreme catalytic proficiency.

## Highlights

• Structural and biochemical characterization of *S. meliloti* choline sulfatase (*Sm*CS)
• *Sm*CS has the typical mechanism and substrate range of an AP-like arylsulfatase
• *Sm*CS is uniquely acting on a primary alkyl sulfate *via* S–O bond cleavage
• The C-terminus is *Sm*CS's business end: tetramerization and active site location

## Graphical abstract

**Figure:**
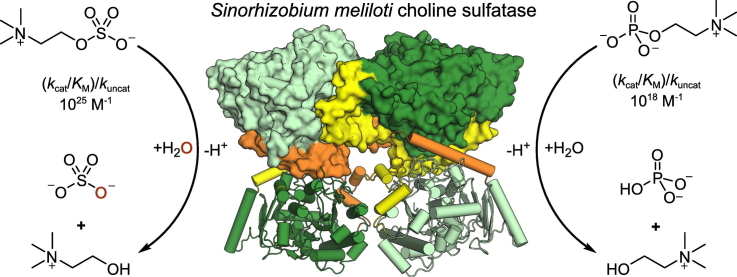

## Introduction

**Fig. 1:**
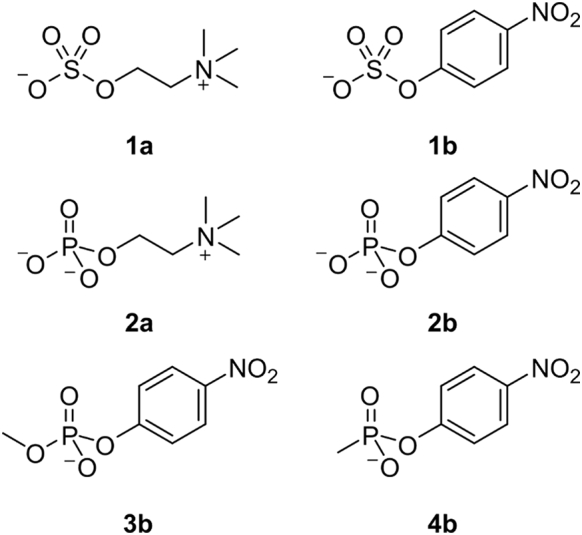
Substrates tested with *Sm*CS WT. **1a**: choline-*O*-sulfate, **1b**: 4-nitrophenyl sulfate, **2a**: phosphoryl choline, **2b**: 4-nitrophenyl phosphate, **3b**: 4-nitrophenyl methylphosphate, **4b**: 4-nitrophenyl methylphosphonate.

Sulfatases are ubiquotous enzymes with a variety or roles in eukaryotic and prokaryotic organisms. In humans, sulfatases are involved in lysosomal degradation of mucopolysaccharides [1], [2] (leading to disease phenotypes when absent [1], [3]), activation of steroid hormones [4], [5] and developmental processes [6], [7], [8], which is mirrored in other vertebrates [9], [10], [11]. Elsewhere, sulfatases play a role in sulfur harvesting [12], [13], [14], [15] and bacterial infection [16], [17], [18], [19]. In many cases, it is not known what the primary sulfate substrate is. The only microbial sulfatases currently assumed to be specific toward one particular substrate are the choline sulfatases (CSs). CSs enable microorganisms to use choline-*O*-sulfate (**1a** in Fig. 1) as a source of sulfur, carbon and nitrogen [20], [21], [22], or contribute to osmoregulation [23].

**Fig. 2:**
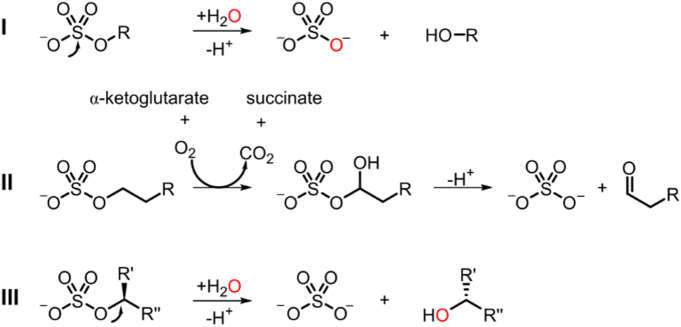
Classification of sulfatases based on their catalytic mechanism [24]. Class I and class III sulfatases catalyze the same net reaction—hydrolytic cleavage of the sulfate ester bond—*via* nucleophilic attack on the S–O and C–O bond, respectively (indicated with an arrow). Class II sulfatases catalyze the cleavage of the sulfate ester bond *via* a reductive dioxygenase mechanism [25].

Sulfatases have been grouped in three classes based on their catalytic mechanism [24]. Class I sulfatases (*aryl*sulfatases, or ASs) hydrolyze a wide variety of sulfate esters by net attack of water on the sulfur center (Fig. 2), resulting in inorganic sulfate and the corresponding alcohol. Structurally and mechanistically, they belong to the alkaline phosphatase (AP) superfamily [26], [27], [28], [29], [30], [31], [32], [33], [34]. Class II sulfatases are specific for *alkyl* sulfates and convert these substrates into inorganic sulfate and the corresponding aldehyde *via* a reductive dioxygenase mechanism (Fig. 2) [25]. Class III sulfatases are also specific for *alkyl* sulfates and catalyze the same net reaction as class I sulfatases [24], but employ a different mechanism (Fig. 2). The active site nucleophile attacks at carbon rather than at the sulfur [35], [36], possibly exploiting the higher reactivity at the carbon center compared to sulfur in alkyl sulfates [37]. The latter difference is reversed in *aryl*sulfates: resonance effects lower the reactivity of the carbon center in the aryl group. The same resonance effects facilitate leaving group departure for the reaction that involves the sulfur center. Hydrolysis of the sulfate ester bond in sulfated sugars has thus far only been shown to be catalyzed by ASs, suggesting that enzyme-catalyzed hydrolysis of sulfated sugars mainly proceeds *via* S–O attack (possibly due to steric hindrance preventing attack at the carbon center).

As described above, CSs enable bacteria to use choline-*O*-sulfate as a source of sulfur, carbon and nitrogen [22], [38]. Expression of CS-encoding genes in *Pseudomonads* is exclusively induced by its substrate choline-*O*-sulfate [20], [21], [22], [38], preventing the energetically costly production of an enzyme that is only useful when its eponymous substrate is available. This phenomenon can be explained by the presence of the transcriptional regulator BetR, which promotes transcription of its own gene (*betR*) and the CS-encoding gene *betC* in the presence of choline-*O*-sulfate [20], [21]. Osteras *et al*. [23] showed that in addition to enabling *Sinorhizobium meliloti* to use choline-*O*-sulfate as a resource, CS is also involved in osmoregulation. In this bacterium, the product of CS-catalyzed hydrolysis of choline-*O*-sulfate, choline, can be readily converted into glycine betaine, which the organism could use either as an osmoprotectant or as a source of carbon and nitrogen.

Based on sequence homology analysis, *S. meliloti* CS (*Sm*CS) was identified as a member of the AP superfamily and is most closely related to class I ASs [23]. However, its proposed native substrate is a primary *alkyl* sulfate, paradoxically a substrate expected to be converted by a class III sulfatase. This poses the three questions: (i) Can CS catalyze the hydrolysis of non-natural *aryl*sulfates such as 4-nitrophenyl sulfate (**1b**, Fig. 1) or coumarin sulfate, a phenomenon observed for several of the class I sulfatases that act primarily on sulfated sugars [27], [39], [40]? (ii) Is CS catalytically promiscuous, a widely observed phenomenon among AP superfamily members [26], [41], [42], [43], [44], [45], [46], [47], [48]? (iii) Does CS act on its native substrate with a similar mechanism as its close relatives from the AP superfamily, that is, by nucleophilic attack on the sulfur? The latter is expected to be 14.2 kcal mol^− 1^ more difficult than attack on carbon and fission of the C–O bond [37] (for details, see supporting information, SI). Nucleophilic attack on the sulfur center in *alkyl*sulfates has been previously shown for a class I sulfatase. *Pseudomonas aeruginosa* AS (PAS) had been shown to catalyze the hydrolysis of secondary alkyl sulfate esters with retention of configuration around the carbon center [36], suggesting that this enzyme indeed hydrolyzes alkyl sulfates *via* S–O cleavage. However, the majority of enzymes that are able to catalyze the hydrolysis of alkyl sulfates are expected to be class III sulfatases [24]. The enzymes in class III indeed employ nucleophilic attack on carbon, shown by both H_2_^18^O labeling studies and the inversion of configuration at the carbon atom directly connected to the leaving group for the enantioselective hydrolysis of *sec*-alkyl sulfates [35], [49].

In order to resolve whether CS is acting as a class I or class III sulfatase and to determine its relationship to the AP superfamily, we have characterized *Sm*CS [23] biochemically and structurally. In this study, we show that the enzyme is a tetramer and that the quaternary structure is largely mediated by a C-terminal extension. These features match those of the tetrameric phosphonate monoester hydrolases (PMHs) and the recently discovered class of dimeric ASs [26], both members of the AP superfamily. However, the oligomeric topology of *Sm*CS presented in this study is significantly different and the oligomer interface plays a far more important role in the formation of the active site cavity. Analysis of the incorporation of oxygen isotope from H_2_^18^O showed that the enzyme attacks choline-*O*-sulfate at the sulfur center rather than at the chemically favored carbon atom and kinetic measurements for a series of arylsulfate and arylphosphate esters further confirmed its relation to class I ASs. Thus, the structural, phylogenetic and mechanistic analysis presented here, as well as the enzyme's promiscuity profile confirm the close relation of CS to class I ASs and validate it as a member of the AP superfamily.

## Results and Discussion

### *S. meliloti* BetC is a choline sulfatase

*Sm*CS, encoded by the *betC* gene, has been identified as a sulfatase of the AP superfamily [23], [27]. We cloned *betC* into a protein production vector resulting in an N-terminal Strep-tag fusion and overexpressed the *Sm*CS-encoding gene in *Escherichia coli*. *Sm*CS contains the Cys-X-Pro-X-Arg motif that is the target of the formyglycine (fGly) generating enzyme (FGE) that catalyzes the conversion of the cysteine in the recognition motif into fGly. ASs [1], [50], [51] and PMHs [47], [52] are well known to carry this post-translational modification that is brought about in *E. coli* by an unknown enzyme endogenous to the protein production host. Since the fGly modification is often incomplete [26], [47], [52], we overexpressed the *Sm*CS-encoding gene in an *E. coli* strain that also produces the FGE from *Mycobacterium tuberculosis* H37v (*Mtb*FGE) [53]. *E. coli*-produced *Sm*CS was purified with a typical yield of 6 mg of pure protein per 1 g of cells. The purified enzyme showed high activity toward choline-*O*-sulfate (sulfate monoester **1a** in Fig. 1) and *k*_cat_/*K*_M_ values varied between 1.2 × 10^3^ and 4.8 × 10^3^ s^− 1^ M^− 1^ for pH 6.8–8.8 (Fig. S1, Table S1), with a *k*_cat_ of 2.4 s^− 1^ and a *K*_M_ of 0.50 mM (*k*_cat_/*K*_M_ = 4.8 × 10^3^ s^− 1^ M^− 1^) at the optimum pH (7.6) for *k*_cat_/*K*_M_ (Table 1, Fig. S1).The catalytic efficiency (*k*_cat_/*K*_M_) toward choline-*O*-sulfate at optimum pH was ~ 200-fold higher than previously reported for the same enzyme fused to a C-terminal His-tag and produced without co-expression of a gene encoding a FGE [54] (Table S3), both of which are expected to affect the activity of *Sm*CS (see SI for a more detailed explanation). However, based on the incomplete fGly modification observed in the X-ray structure (see below for details), the true catalytic efficiency of the fGly-form of *Sm*CS WT may be higher than the activity levels observed here.

**Table 1:** Native and promiscuous reactions of wild-type *Sm*CS

pH	Substrate	*k*_cat_ (s^− 1^)	*K*_M_ (M)	*k*_cat_/*K*_M_ (s^− 1^ M^− 1^)	(*k*_cat_/*K*_M_)/*k*_1_^a^ (M^− 1^)	(*k*_cat_/*K*_M_)/*k*_2_^a^
7.6^b^	**1a**	2.4 ± 0.2	(5.0 ± 0.6) × 10^− 4^	(4.8 ± 0.5) × 10^3^	1.7 × 10^25^	9.6 × 10^26^
	**2a**	(2.67 ± 0.01) × 10^− 2^	(2.1 ± 0.2) × 10^− 3^	13 ± 1	2.7 × 10^18^	1.5 × 10^20^
6.0^c^	**1b**	0.22 ± 0.01	(1.9 ± 0.2) × 10^− 2^	12 ± 2	6.3 × 10^10^	3.5 × 10^12^
	**2b**	n.a.	n.a.	< 7.5 × 10^− 4^^d^	< 5.6 × 10^5^	< 3 × 10^7^
	**3b**	> 4 × 10^− 3^	> 5 × 10^− 2^	(8.0 ± 0.2) × 10^− 2^	2.2 × 10^8^	1.1 × 10^9^
	**4b**	0.1 ± 0.01	(6.7 ± 0.8) × 10^− 2^	1.5 ± 0.3	1.3 × 10^9^	7.0 × 10^10^

a For details on *k*_1_ (=* k*_uncat_, a first order rate constant for hydrolysis) and *k*_2_ (=* k*_w_, the second-order reaction rate constant for the reaction of H_2_O), see Table S2 for details.

b 100 mM Tris–HCl (pH 7.6) at 25 °C.

c 100 mM imidazole–HCl (pH 6.0) at 25 °C.

d See supporting information for the detection limits for enzyme-catalyzed hydrolyses of the various sulfo- and phosphoesters.

CS had been previously identified as a member of the AP superfamily closely related to the AS subgroup in this enzyme superfamily [23]. These enzymes have been assigned ASs based on their ability to catalyze the hydrolysis of non-natural aryl sulfates, such as 4-nitrophenyl sulfate (**1b**, Fig. 1) [26], [46], and coumarin sulfate [27]. *Sm*CS was shown to be active toward aryl sulfate monoester **1b**. However, its second-order rate constant (*k*_cat_/*K*_M_ = 12 s^− 1^ M^− 1^ at pH 6.0; Fig. S1, Table 1) was considerably lower than for choline-*O*-sulfate (**1a**). Mutations in the active-site residues that are conserved between *Sm*CS and *Sp*AS1 [26], [55] decreased catalytic efficiencies toward 4-nitrophenyl sulfate hydrolysis (Table 2), confirming that *Sm*CS, and not a contaminating enzyme, is catalyzing the hydrolysis of aryl sulfates. The activity toward sulfate monoester **1b** is modest compared to the catalytic efficiencies of many of its family members (*k*_cat_/*K*_M_ ~ 10^3^–10^7^ s^− 1^ M^− 1^). We tested alkyl sulfates **1c**–**1h** (Fig. S2) for activity with *Sm*CS. No conversion of any of these sulfate esters could be detected after 24 h (suggesting a *k*_cat_/*K*_M_ < 4 × 10^− 2^ s^− 1^ M^− 1^; see SI for details). Even 3,3-dimethyl butyl-*O*-sulfate **1c** (Fig. S2), isosteric to choline-*O*-sulfate, is not converted, suggesting that *Sm*CS hydrolyzes alkyl sulfates only when substrate carries the positively charged quaternary ammonium group.

**Table 2:** Kinetic parameters^a^ for enzyme-catalyzed hydrolysis of sulfate monoesters **1a** and **1b** for *Sm*CS wild type and mutants

	Choline-*O*-sulfate **1a**				4-Nitrophenyl sulfate **1b**			
	*k*_cat_ (s^− 1^)	*K*_M_ (M)	*k*_cat_/*K*_M_ (s^− 1^ M^− 1^)	ΔΔ*G*_mut_ (kcal mol^− 1^)^b^	*k*_cat_ (s^− 1^)	*K*_M_ (M)	*k*_cat_/*K*_M_ (s^− 1^ M^− 1^)	ΔΔ*G*_mut_ (kcal mol^− 1^)^b^
WT	2.4 ± 0.2	(5.0 ± 0.6) × 10^− 4^	(4.8 ± 0.7) × 10^3^	n.a.	(3.4 ± 0.7) × 10^− 1^	(9.9 ± 1.9) × 10^− 2^	3.4 ± 1.0	n.a.
C54A			≥ 2 × 10^− 3^	< 8.7			< 3.8 × 10^− 5^	> 6.7
C54S	(1.12 ± 0.02) × 10^− 2^	(9.4 ± 0.1) × 10^− 3^	1.19 ± 0.02	4.9	(7.3 ± 0.1) × 10^− 5^	(3.9 ± 0.2) × 10^− 2^	(1.89 ± 0.06) × 10^− 3^	4.4
K102 L			< 7.5 × 10^− 5^	> 10.7			< 3.8 × 10^− 5^	> 6.7
H104A	(3.47 ± 0.01) × 10^− 2^	(1.33 ± 0.01) × 10^− 2^	2.60 ± 0.02	4.4	(7.8 ± 0.3) × 10^− 4^	(2.02 ± 0.01) × 10^− 1^	(3.8 ± 0.1) × 10^− 3^	4.0
H201A			≥ 7 × 10^− 4^	< 9.3			< 3.8 × 10^− 5^	> 6.7
K309 L			≥ 2 × 10^− 3^	< 8.7	(1.6 ± 0.5) × 10^− 5^	(8.9 ± 2.7) × 10^− 2^	(1.76 ± 0.01) × 10^− 4^	5.8
E386L	> 1.2 × 10^− 2^	> 1.5 × 10^− 2^	0.81 ± 0.01	5.1	(7.6 ± 0.3) × 10^− 1^	(5.7 ± 0.4) × 10^− 2^	(1.3 ± 0.1) × 10^1^	− 0.79
Δ12	(2.1 ± 0.2) × 10^− 2^	(1.09 ± 0.01) × 10^− 2^	1.93 ± 0.01	4.6	(1.59 ± 0.01) × 10^− 2^	(9.0 ± 0.1) × 10^− 2^	(1.78 ± 0.04) × 10^− 1^	1.8
Δ23			≥ 1 × 10^− 2^	< 7.7			(4.77 ± 0.03) × 10^− 3^	3.9

a Recorded at 25 °C in 100 mM Tris–HCl (pH 7.6).

b ΔΔ*G*_mut_ = RTln[(*k*_cat_/*K*_M_)_WT_/(*k*_cat_/*K*_M_)_mutant_] = 1.36Log[(*k*_cat_/*K*_M_)_WT_/(*k*_cat_/*K*_M_)_mutant_].

### Structure of *Sm*CS

**Fig. 3:**
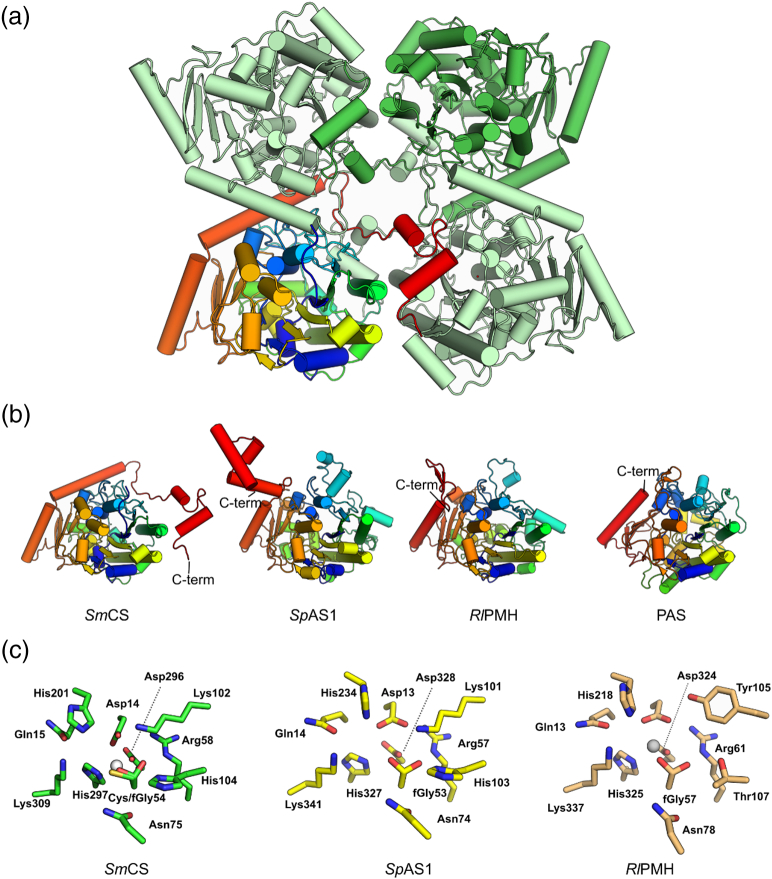
Structure of *Sm*CS. (a) Quaternary structure of *Sm*CS with one of the protomers coloured in a rainbow pattern from blue at the N-terminus to red in the C-terminus. (b) Similarity to other AP superfamily member protomers: AS from *S. pomeroyi* (*Sp*AS1, PDB: 4UPI[26]), PMH from *R. leguminosarum* (*Rl*PMH, 2VQR [52]) and AS from *P. aeruginosa* (PAS, 1HDH [28]). All four structures are shown in the same orientation, with blue-to-red rainbow coloring from N- to C-terminus. The C-terminal extensions that mediate oligomerization are visible in all structures with the exception of the monomeric PAS. C-termini of all structures are labeled for clarity. (c) Conserved active site residues of CS *Sm*CS, AS *Sp*AS1 and PMH *Rl*PMH (see also Table S7).

In order to understand the determinants of substrate specificity and catalytic mechanism, we crystallized *Sm*CS and determined its three-dimensional structure by molecular replacement and refined the structure to 2.79-Å resolution (Table S4, Fig. 3). The enzyme crystallizes with eight molecules in the asymmetric unit, and analysis of the structure using the PISA server [56] suggests that the protein is tetrameric with each of the protomers containing two oligomerization interfaces of ~ 2800 and ~ 1600 Å^2^ (see Figs. S3–S4 and Table S5 for details). This is consistent with analysis of *Sm*CS by size-exclusion chromatography and multi-angle laser light scattering (Fig. S5), which shows that the protein forms tetramers in solution with *ca*. 240-kDa molecular mass (*M*_w_ of a protomer ~ 61 kDa).

The protomer structure of *Sm*CS shows a globular protein with an α/β fold, a central β-sheet surrounded by α-helices, characteristic to proteins in the AP superfamily [30], [31], [42], confirming that *Sm*CS is indeed a member of this superfamily. Similarity to the AS/PMH subgroup of the AP superfamily allows identification of the active site of *Sm*CS by comparing the enzyme with *Silicibacter pomeroyi* AS1 [26] (*Sp*AS1) and *Rhizobium leguminosarum* PMH [52] (*Rl*PMH) (Fig. 3c). The active site residues of *Sm*CS match completely with the conserved active site residues of *Sp*AS1 and are identical in 9 out of 11 residues in *Rl*PMH (Tables S6–S7). These conserved active site residues include residues Asp14, Asp296 and His297 that are expected to coordinate a divalent metal ion. MicroPIXE analysis (particle-induced X-ray emission) of the purified enzyme showed a mixture of Ca, Fe, Mn and Zn (Table S8) with occupancies similar to the ones published for the PMHs [26], [47], [52] and dimeric ASs [26] (Table S8). The nucleophile (Cys54/fGly54) is coordinated to His104 and the metal ion. Well-defined and continuous electron density was observed for the active site residues (Asp14, Cys54 and His297) that tetrahedrally coordinated a metal ion. This metal was thus refined as Ca^2 +^, because it predominated in microPIXE experiments (Table S8), presumably reflecting its high concentration in the 2 × YT medium, and this was the metal ion used for modeling the crystal structure. The density around the metal was however less well defined than other parts of the protein, most likely reflecting the fact that the metal site was not fully occupied and therefore the coordinating residues were not in a single fixed conformation, held in place by the metal. However, activity measurements after the addition of metal ions detected in PIXE experiments showed the largest additional rate enhancements for Mn^2 +^, suggesting that it is the likely candidate for the catalytic metal ion (Fig. S6), consistent with the vicinity to Mn^2 +^-containing PMHs and dimeric ASs indicated in the phylogenetic tree (Fig. 8). Additional positive density was observed in the vicinity of Cys54 (Fig. S6), suggesting that the latter was partially converted into fGly during production in *E. coli*. Given that the exact level of fGly conversion is unknown, the structure was modeled with both cysteine and formyl glycine in this position, giving them each 50% occupancy. The resulting model is in good agreement with the electron density around the active site and reflects our understanding of the active site heterogeneity, even if it cannot be taken as an accurate measure of it.

The active site is completed by Lys102 and Asn75, which have been postulated to interact with the non-bridging oxygens of the sulfate ester substrate [26], [28], and the His201–Lys309 general acid pair that is likely to be responsible for the protonation of the leaving group as the transition state is approached [58]. While enzyme-catalyzed hydrolysis of the model substrate **1b** with its nitrophenolate leaving group (p*K*_a_ 7.01 [59]) may not rely very strongly on this catalytic feature, protonation of the energetically disfavored alkoxide anion expected to be formed during *Sm*CS-catalyzed hydrolysis of choline-*O*-sulfate **1a** (p*K*_a_ cholate leaving group: 13.9 [60]) is essential for efficient catalysis. Lys309 interacts directly with Gln15 as described previously for the analogous active site in *Rl*PMH (residues Lys337 and Gln13) [52].

### Oligomerization facilitated by the C-terminal region

The most noticeable structural differences between subgroups within the AP-type ASs and PMHs are in their C-termini, which differ in both length and sequence. In the case of *Sm*CS, the C-terminal tail folds almost around the whole protomer and contains three α-helices that mediate oligomerization (for more details see below). Enzymes of a recently described sulfatase subgroup also contain a C-terminal α-helical tail, but in that particular case, it adopts an entirely different conformation. In PMHs, the tail is composed of the short β-hairpin, whereas monomeric PAS lacks the extended tail entirely (Fig. 3b).

**Fig. 4:**
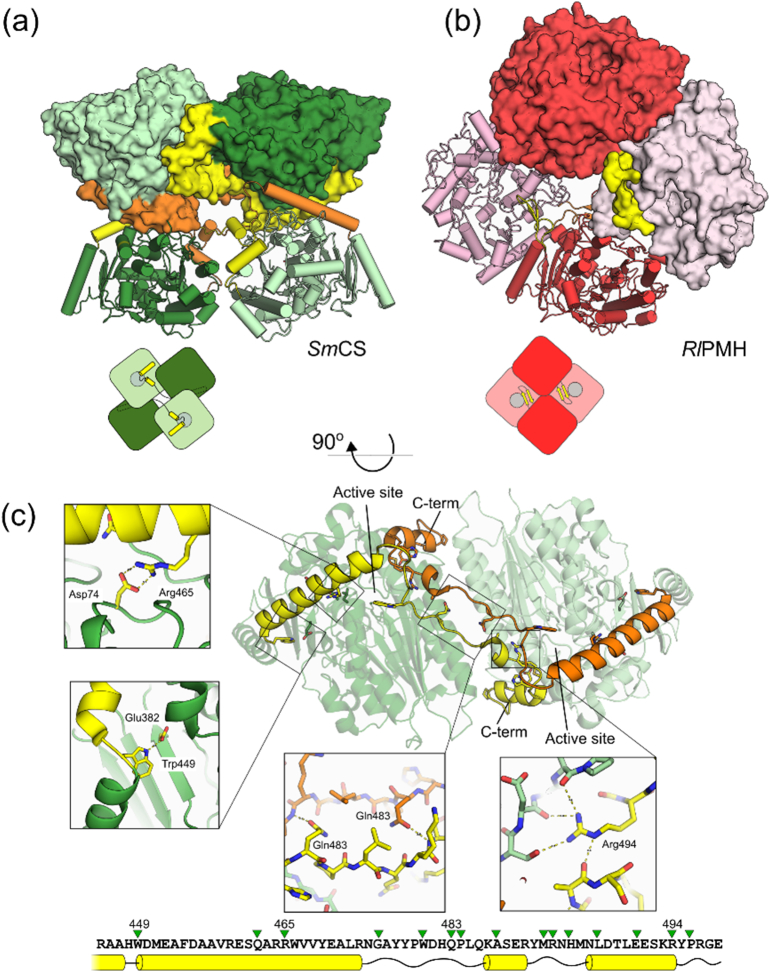
Oligomerization of *Sm*CS. (a) Tetrameric *Sm*CS shown with two protomers with the large dimer interface rendered as cartoon diagrams or with molecular surface. For each dimer, the C-terminal extensions mediating dimerization are depicted in yellow and orange respectively. (b) Tetrameric *Rl*PMH [52] with the dark red cartoon protomer shown in the same orientation as the dark green protomer of *Sm*CS. As in panel a, the two dimers with large interaction interface are shown as cartoons or with molecular surface, and color in dark and light red, with C-terminal extensions in yellow and orange. The small diagram underneath the oligomers highlights the difference in oligomeric organization between the two proteins. (c) C-terminal tail of *Sm*CS. Cartoon diagram of the lower dimer from panel a rotated 90° along the horizontal axis showing the two intertwined C-terminal extensions in yellow and orange. The zoomed-in regions show details of some of the fully conserved residues that mediate the interaction of the C-terminus with the globular core domain. All 100% conserved residues in the tails of all CSs are indicated above the sequence of the *Sm*CS tail with green triangles (see also Fig. S8).

We have previously shown that different subgroups of AP superfamily-type sulfatases and PMHs differ in their quaternary structures. *Sm*CS is extending the scope of oligomeric forms further. While *Sm*CS and PMHs are both tetrameric, they assemble into their respective oligomeric structures very differently (Fig. 4a and b). Both can be seen as a dimer of dimers, and in both cases, the C-termini interact with another protomer, yet the larger protein–protein interface that mediates further oligomerization is entirely different. In *Sm*CS, the C-terminal region (residues 449–512) appears to be part of the small and large oligomerization interfaces. The long α-helix (residues 451–473) at the start of the C-terminal tail directs the tail toward the other protomer and itself is part of the interface with the second dimer that makes up the tetramer; that is, it is involved in forming the dimer of dimers. The bulk of the larger interface (red in Fig. S3a) is formed by head-to-tail interactions between two long α-helices (residues 160–182) and the C-termini that cross from one protomer to another, interacting with each other in the middle. Many of the residues that form specific interactions in the tail appear to be highly conserved in CSs, indicating that this form of oligomerization is conserved among these enzymes (Figs. 4c and S8). In particular, Trp449 appears to guide the long α-helix to its intended direction, resulting in fully conserved ionic interaction between Arg465 in the C-terminal tail and Asp74 in the core of the domain in the opposing protomer help to maintain its position. Two Gln483 side chains from different protomers hydrogen bond across the dimer interface, and Arg494 forms multiple hydrogen bonds with the opposing domain (Fig. 4c).

**Fig. 5:**
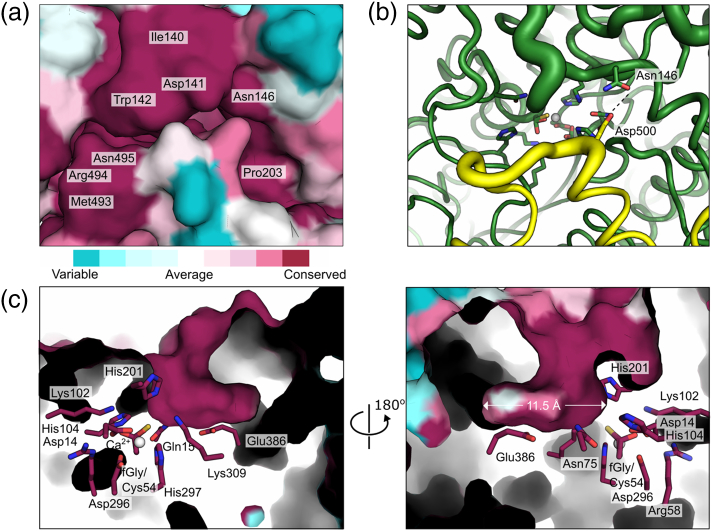
Active site entrance in *Sm*CS. (a) Surface representation of the entrance to the active site of *Sm*CS, colored by conservation among all CSs, as calculated using the ConSurf server [61], [62], [63], [64]. One hundred percent conserved residues are labeled on the surface. The color scale, from cyan to dark purple (in nine steps ranging from variable to fully conserved), is shown underneath. (b) Same view of the active site entrance as in panel a, with the backbone diameter reflecting variations in *B*-factors (i.e., higher diameter depicts higher *B*-factors). The tail of the incoming protomer is shown in yellow. Conserved residues Asn146 and Asp500 (see Fig. S8) forming a hydrogen bond across the opening are shown as sticks. The conserved active site residues (i.e., those conserved between CSs and ASs) are shown underneath the entrance site. (c) Cut-out of the active site cavity of *Sm*CS from two different, 180° rotated views, with active site residues shown as sticks. The divalent metal ion in the active site was fitted as Ca^2 +^, the most abundant metal in microPIXE, even though Mn^2 +^ is metal that, when added, provided the largest rate enhancement (Fig. S6) and is shown as a sphere. Coloring of the surface and carbon atoms in residues is according to the degree of conservation, as in panel a. The width of the active site indicated in the right-hand panel is taken from the surface of the cavity at its longest point. Details on which active site forming residues are conserved can be found in the supporting information (Fig. S8 and Table S9).

The extreme C-termini interact with the loops that cover the entrance of the active site cavity of the other protomers. We analyzed conservation of the residues in and around the active site among all CSs and found 100% conserved residues not only in the active site but also in the entrance to it, both in the subunit with catalytic residues and in the C-terminal tail (Fig. 5a). The active site is closed in this *Sm*CS structure, and the loops that cover the entrance would need to move to allow the subtrate to enter and the products to leave. Analysis of the entrance loops shows significantly higher *B*-factors for the loops that limit the access to the active site, suggesting structural mobility for these structures. A hydrogen bond between Asp500 and Asn146 seems to act as a latch that stabilizes the closed conformation (Fig. 5b). The conservation extends from the mouth all the way to the active site, a narrow L-shaped cavity which is ~ 11.5 Å in its longest dimension, offering sufficient space to accommodate choline-*O*-sulfate (Fig. 5c). Three of the residues that form the tunnel come from the C-terminal region of one of the other protomers (Leu499, Leu502 and Arg507). Most of the tunnel-forming and active site residues are also part of the larger of the two oligomerization interfaces (red in Figs. S3a and S4). The other, more buried part of the active site consists mostly of hydrophobic residues, and Glu386, predicted to position the quaternary amine present in the choline ester sitting at one end (Fig. 5c).

The deeply buried binding pocket of *Sm*CS stands in sharp contrast with the wide open, solvent exposed active site of *Rl*PMH (Fig. S9). In both tetrameric enzymes, the C-terminal tail that promotes the oligomerization extends toward the active site of the opposing protomer. However, the direct and close involvement of the oligomerization interface in the formation of the active site entrance and pocket as seen for *Sm*CS is absent in *Rl*PMH.

Given the apparent critical role of the C-terminus in the formation of entry to the active site of CSs, we explored its role in oligomerization and in enzyme catalysis in more detail. We constructed truncated mutants lacking 12 and 23 C-terminal amino acids, respectively, by replacing Thr501 and Glu490, with stop codons (*Sm*CS Δ12 and Δ23). These two residues were chosen because they are not part of the oligomerization interface itself. The Δ12 mutant was still predominantly present in the tetrameric form (87%). However, a fraction of the enzyme was now present as a dimer (11%) and a monomer (2%) (Fig. S5). The deletion of the last 12 residues was detrimental to the enzymatic activity (Table 2), resulting in a 20- (sulfate monoester **1b**) and a 2.5 × 10^3^- (**1a**) fold decrease in *k*_cat_*/K*_M_, respectively. This decrease could not be explained solely by the lower proportion of the tetrameric state as compared to wild type. The effect of the truncation on catalysis of choline-*O*-sulfate (**1a**) hydrolysis (ΔΔ*G*_mut_ = 4.6 kcal mol^− 1^, Table 2) was significantly greater than for the model substrate 4-nitrophenyl sulfate (**1b**) hydrolysis (ΔΔ*G*_mut_ = 1.8 kcal mol^− 1^; Table 2). The 23-amino-acid C-terminal deletion caused an even more dramatic change in distribution between the various oligomeric states: now the enzyme occurs predominantly in its dimeric form (78%; Fig. S5). Catalysis was affected to a larger extent than for the Δ12 mutant and the native substrate once more suffered a larger decrease than the promiscuous substrate **1b** [ΔΔ*G*_mut_ < 7.7 kcal mol^− 1^ for choline-*O*-sulfate (**1a**); ΔΔ*G*_mut_ = 3.9 kcal mol^− 1^ for 4-nitrophenyl sulfate (**1b**); Table 2]. This observation suggests that the close interaction between oligomerization interface and active site pocket is mainly important for the ability to accept the choline leaving group, while the general ability to hydrolyze sulfate esters remains comparatively less affected.

### Catalytic mechanism

In near neutral aqueous solution, the hydrolysis of alkyl sulfates, such as choline-*O*-sulfate, occurs predominantly *via* nucleophilic attack on the carbon atom next to the bridging oxygen of the sulfate ester group (C–O attack) [37], in contrast to aryl sulfate hydrolysis, which proceeds *via* nucleophilic attack at the sulfur center (breaking the S–O bond). As expected, the recently discovered class III sulfatases catalyze attack at carbon (C–O cleavage) for the hydrolysis of primary and secondary alkyl sulfates [35], [49] (Fig. 2) and are unable to catalyze the hydrolysis of aryl sulfates [24]. *Sm*CS is active toward choline-*O*-sulfate, an alkyl sulfate, but is incapable of converting any other alkyl sulfates. However, the enzyme is able to hydrolyze aryl sulfates. This raises the question whether *Sm*CS should be classed as a class I or III sulfatase. *Sm*CS-catalyzed turnover of choline-*O*-sulfate in the presence of H_2_^18^O showed no incorporation of the ^18^O label in the choline product (Fig. S10), indicating that *Sm*CS hydrolyzes choline-*O*-sulfate *via* S–O cleavage, thus employing a class I mechanism (Fig. 2). The latter observation suggests that *Sm*CS has to overcome an extra 14.2 kcal mol^− 1^ in activation free energy (see SI for details) as a result of using the energetically disfavored route. This means that *Sm*CS has a catalytic proficiency ((*k*_cat_/*K*_M_)/*k*_1_) of 1.7 × 10^25^ M^− 1^ for choline-*O*-sulfate hydrolysis (Table 1); its proficiency toward the more activated 4-nitrophenyl sulfate substrate is substantially lower at 4.6 × 10^9^ M^− 1^. Highly proficient enzymatic S–O attack on alkyl sulfate esters has been reported previously [65], [66], with catalytic proficiencies reaching values as high as 10^29^ M^− 1^ [37]. However, the amino acid sequences of these enzymes were never determined, making *Sm*CS the most proficient fully characterized sulfatase and similarly proficient as fructose-1,6-bisphosphatase ((*k*_cat_/*K*_M_)/*k*_1_ = 1.4 × 10^25^ M^− 1^), one of the most proficient enzymes known to date [67].

S–O cleavage of alkyl sulfate esters has thus far only been reported for PAS [36] (although some reports make this claim without providing a gene sequence [65], [66]). We tested PAS, *Sp*AS1 [26], *Sp*AS2 [26] and *Rl*PMH [52], all of which are members of the AP superfamily that are expected to employ the class I mechanism (Fig. 2), for activity toward choline-*O*-sulfate. No choline formation could be detected with any of these enzymes (*k*_cat_/*K*_M_ < 7.5 × 10^− 5^ s^− 1^ M^− 1^, see SI for details on the detection limit for CS activity). Class III alkylsulfatases Pisa1 [49], [68] and SdsA1 [24] also showed no detectable activity toward choline-*O*-sulfate. The similarity of *Sm*CS to ASs (in terms of overall structure as well as the catalytic residues) supports the case for for S–O attack and is also consistent with the effect mutations in residues that are conserved between *Sm*CS and ASs (i.e., all mutants expect E386L, Δ12 and Δ23 as shown in Table 2) have on *Sm*CS-catalyzed conversion of choline-*O*-sulfate. By contrast, alkylsulfatases known to utilize C–O cleavage do not catalyze the hydrolysis of 4-nitrophenyl sulfate [69], underlining the diagnostic value of this reaction.

In order to test whether the active-site residues conserved between *Sm*CS and the previously described ASs [26], [27], [28] (i.e., all mutants in Table 2 except E386L, Δ12 and Δ23) perform the same function, we mutated several of them and determined kinetic parameters for the enzyme-catalyzed hydrolysis of sulfate monoesters **1a** and **1b** at pH 7.6 (Table 2). The effects of mutating these five residues were largely similar for both substrates (4 to > 10.7 kcal mol^− 1^ and 4 to > 6.7 kcal mol^− 1^ for sulfate monoesters **1a** and **1b**, respectively), suggesting that they are converted *via* the same mechanism. The enzyme-catalyzed conversion of 4-nitrophenyl sulfate (**1b**) is inhibited in the presence of choline (Fig. S11), also indicating that both substrates use the same active site. The different pH-rate profiles (Fig. S1), however, suggest that one or more different steps are limiting for catalysis.

**Fig. 6:**
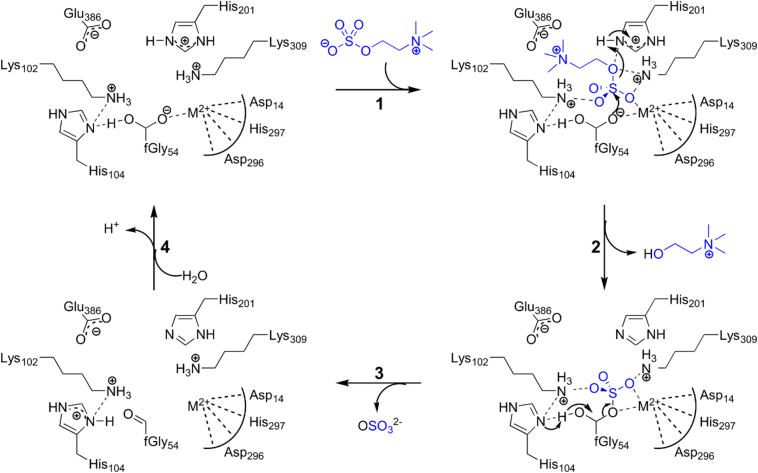
Proposed mechanism for choline-*O*-sulfate hydrolysis by *Sm*CS. The substrate choline-*O*-sulfate binds *via* charge–charge interactions of its (i) positively charged quaternary ammonium group with glutamate 386 (Glu386) and (ii) its negatively charged sulfate group with several positively charged amino acids and a divalent metal ion, M^2 +^) (step **1**). The substrate is subsequently attacked by the hydrated formylglycine (fGly54) nucleophile and choline departs assisted by leaving group stabilization by the His201–Lys309 pair (**2**). The covalent intermediate is then resolved by general base-catalyzed hemiacetal cleavage (**3**), as has been suggested for other ASs [27], followed by rapid rehydration of the formylglycine aldehyde (**4**). The identity of the metal ion is unclear. Addition of Mn^2 +^ brings about a threefold to fourfold rate enhancement. Addition of excess Cu^2 +^, Co^2 +^ and Fe^2 +^ also results in increased catalytic activity, whereas excess Mg^2 +^ or Ca^2 +^ has no and Zn^2 +^ an inhibitory effect, suggesting that Mn^2 +^ is the preferred catalytic metal. However, microPIXE analysis also indicated the presence of Ca^2 +^, Zn^2 +^ and Fe^2 +^ besides Mn^2 +^, of which Ca^2 +^ was the most abundant (presumably refelecting its high concentration in the 2YT medium used). Therefore the X-ray structure was refined as a Ca^2 +^-containing species (see Materials and Methods and Table S8 for details).

As mentioned above, the conserved active site residues sit at the corner of an access tunnel connecting to the solvent and a buried active pocket that is largely hydrophobic, with a glutamate (Glu386) sitting ~ 7 Ǻ away from the His201–Lys309 general acid pair that is thought to protonate the alkoxy leaving group of the substrate (analogous residues have been suggested for several AP-type sulfatases [26], [27], [28]) (Fig. 6). The ~ 10^3^-fold drop in catalytic efficiency (*k*_cat_/*K*_M_) in the Glu386Leu mutant (Table 2) is consistent with a role in catalysis, either *via* ground state binding of the substrate or *via* assisting with the correct positioning of the alkoxy leaving group for general acid catalysis by the His201–Lys309 pair upon departure. Furthermore, the enzyme-catalyzed hydrolysis of aryl sulfate monoester **1b** is virtually unchanged for this mutant, confirming that Glu386 is only important for choline-*O*-sulfatase activity. Choline binding in other proteins has often been attributed to interactions of the quaternary ammonium with π-electrons from aromatic amino acid side chains [70], [71], [72], [73], [74], [75], [76], [77], sometimes in combination with binding to negatively charged amino acids [78], [79]. The latter is the case for *P. aeruginosa* phosphorylcholine phosphatase, in which two glutamates and a tyrosine are shown to contribute to binding of the quaternary ammonium group [78], [80]. However, the effect of mutation of either of these residues is small (ΔΔ*G*_mut_ ~ 0.4–2.1 kcal mol^− 1^ per residue, based on data from Beassoni *et al*. [80]), compared to the effect of the Glu386Leu mutation in *Sm*CS (ΔΔ*G*_mut_ = 5.1 kcal mol^− 1^; Table 2). Based on the mutagenesis of the active site residues and their similarity to the active site residues in related ASs and PMHs (Fig. 3, Table S7), we propose that the catalytic cycle for *Sm*CS-catalyzed hydrolysis of choline-*O*-sulfate is similar to that of the AS-catalyzed hydrolysis of aryl sulfates [26], [55], [58] and the *Rl*PMH-catalyzed hydrolysis of phosphodiesters [52]. The specificity toward choline-*O*-sulfate is determined by the interaction of a negatively charged glutamate (Glu386) with the positively charged quaternary ammonium group of the substrate (Fig. 6).

### Emergence of the CSs within the AP superfamily

**Fig. 7:**
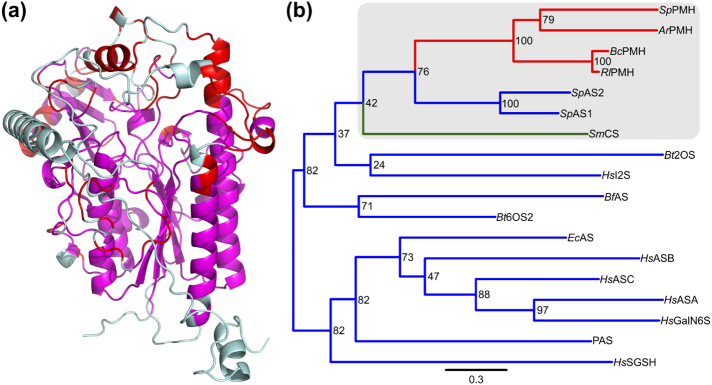
Structural alignment of *Sm*CS with all known AS (13) and PMH (4) structures (see SI and Table S6, S7, S10 and S11 for details). (a) Structure of a single *Sm*CS protomer with the regions that align with all 17 structures included in the multiple structural alignment indicated in magenta. In addition, the regions indicated in red align only with the two dimeric ASs (*Sp*AS1 and *Sp*AS2) and the four PMHs. (b) Phylogenetic relationship between all 18 enzymes based on the structural alignment. Only the positions that aligned structurally in all 18 proteins were considered (268 positions in total, indicated in magenta in panel a). A more expanded version (additional sequences) of the area indicated in grey is shown in Fig. 8.

As reported previously [23], [54] and confirmed in our study by the similarities of the three-dimensional structure (Fig. 3), its catalytic mechanism (Fig. 6) and the conserved active site residues (Table S7), *Sm*CS is a member of the AP superfamily and belongs to the AS/PMH subgroup within that superfamily. A structural alignment and resulting phylogenetic tree that includes *Sm*CS and all AP-type ASs and PMHs of known structure confirmed its close relation to the recently discovered dimeric ASs and tetrameric PMHs (Fig. 7) [26].

To further explore the relationship between CSs, PMHs and dimeric ASs, we created a multiple-sequence alignment of 87 (putative) CSs with all 85 (putative) PMHs and 95 (putative) ASs contained in the previously reported phylogenetic relationship between these two subclasses of the AP superfamily [26], in effect expanding that phylogenetic tree with the CSs. The putative CS-sequences were obtained from a BLAST search with the *Sm*CS sequence as bait on the available genome databases. Since the previously published AS/PMH tree contained only sequences from α- and β-proteobacteria [26], we limited the searchable dataset to those two bacterial classes. The final alignment included 60 sequences from α-proteobacteria (including *Sm*CS), 27 sequences from β-proteobacteria and all the previously identified (putative) ASs [26] and PMHs [26], [47], [52], [57] (Tables S12–S14).

The multiple-sequence alignment shows a high degree of conservation within the CS group (62%) compared to the average pairwise sequence identity of the complete alignment (36%) and the 51% and 50% identities within the AS and PMH clades, respectively. The active site residues as listed in Table S8 are 100% conserved for all CSs and align perfectly with the identical positions in ASs and PMHs. The residues that form the L-shaped active site in *Sm*CS are largely conserved for all (putative) CSs (Fig. S8, Table S9). The C-terminal tail (Figs. 4c and S3d) of the CSs shows a similar degree of sequence conservation to the CSs for the complete alignment (57% *versus* 62%), whereas for the PMHs (39% *versus* 50%) and the dimeric ASs (27% *versus* 51%), there is much more variability in the C-terminal region. The latter is in agreement with the fact that the CS-specific conserved residues that form the hydrophobic part of the active site and the active site tunnel are also part of the oligomerization interface that is largely formed by the C-terminus.

**Fig. 8:**
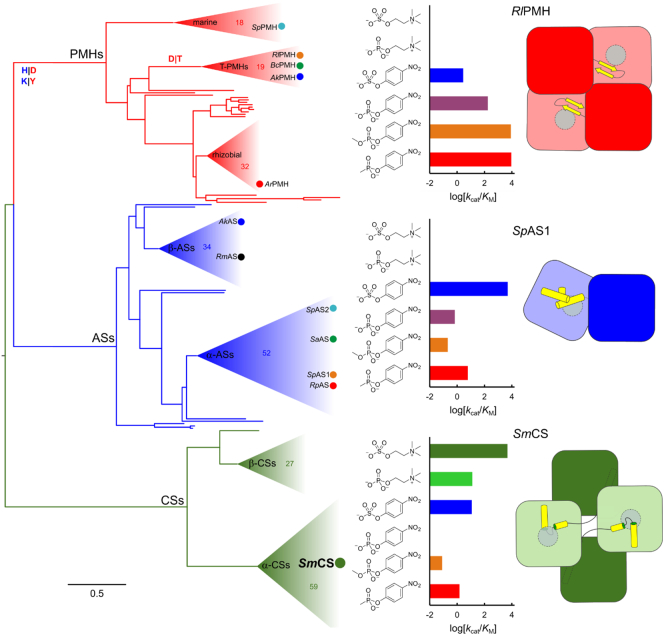
Maximum likelihood phylogenetic tree of the relationship between PMHs [26], [47], [52], [57], dimeric ASs [26] and CSs. Filled circles represent the extant, characterized members of the superfamily. The PMHs and the ASs show the same internal phylogeny as described previously [26]. The CSs show a clear division between enzymes originating from α- and β-proteobacteria (α-CSs and β-CSs, respectively), similar to the subdivision within the dimeric ASs. Representative substrate specificity profiles for each subgroup suggest that the divergence between sulfatases and PMHs is accompanied by a shift in substrate preference. In contrast, the emergence of enzyme-catalyzed hydrolysis of choline ester substrates appears to be unique to the CS clade. All three subgroups show involvement of the C-terminal regions in oligomerization. However, like the amino acids in the C-terminal regions, the eventual quaternary structures are highly divergent between the three subgroups. See Figs. S12–S14 and Table S12–S14 for details on the sequences included.

The phylogenetic tree based on the multiple-sequence alignment shows CSs, ASs and PMHs as three distinct phylogenetic clades (Fig. 8). The subdivisions within the dimeric ASs and PMHs are essentially similar to the ones that were observed when only ASs and PMHs were included in the alignment [26]. The CSs are a very distinct group and appear genetically further removed from the ASs and PMHs than those two classes are from each other. The CS clade shows a clear distinction between enzymes originating from α- and β-proteobacteria (Fig. 8).

### Quantification of catalytic promiscuity of *Sm*CS

A defining feature of the phospho- and sulfohydrolases of the AP superfamily is the ability to catalyze multiple hydrolytic reactions with substantial rate accelerations [42], [81]. The observation of promiscuity has been named “crosswise”; that is, the primary activities of the various family members are also promiscuous reactions in other family members [26], [41], [42], [43], [44], [45], [46], [47], [48], [55]. *Sm*CS had previously been shown to be active toward phosphoryl choline (**2a**, Fig. 1). These data were obtained with cell extracts [23], which means that enzymatic activity of other phosphatases present in cell extract could lead to overestimation of the actual *Sm*CS-catalyzed phosphorylcholine hydrolysis. We confirmed the activity toward phosphoryl choline **2a** for the purified enzyme and showed that *Sm*CS converts this substrate with a *k*_cat_ of 0.027 s^− 1^ and a *K*_M_ of 2.1 mM (*k*_cat_/*K*_M_ = 13 s^− 1^ M^− 1^; Table 1). The catalytic proficiency ((*k*_cat_/*K*_M_)/*k*_1_) of 2.7 × 10^18^ M^− 1^ for *Sm*CS toward this promiscuous substrate rivals that of primary activities of the highly proficient carboxypeptidase b and phosphotriesterase [67]. No turnover was detected for the more reactive nitrophenyl ester **2b** lacking the positive charge (*k*_cat_/*K*_M_ < 7.5 × 10^− 4^ s^− 1^ M^− 1^; see SI for a consideration of detection limits). When the previously observed correlation between the catalytic efficiencies (*k*_cat_/*K*_M_ values) of sulfatase-catalyzed hydrolysis of sulfate monoester **1b** and phosphate monoester **2b** [26] is extrapolated to the *k*_cat_/*K*_M_ value toward **1b** for *Sm*CS, the value for **2b** is around 10^− 3^ s^− 1^ M^− 1^ (Fig. S15a), close to the detection limit of 7.5 × 10^− 4^ s^− 1^ M^− 1^, which is in agreement with typical class I sulfatase substrate specificity between sulfate monoester **1b** and phosphate monoester **2b**. Phosphate diester **3b** and phosphonate monoester **4b** were hydrolyzed by *Sm*CS with *k*_cat_/*K*_M_ values of 0.02 and 1.5 s^− 1^ M^− 1^, respectively (measured at the optimum pH for sulfate monoester **1b,** pH 6.0; Table 1), corresponding to catalytic proficiencies ((*k*_cat_/*K*_M_)/*k*_1_) of 5.5 × 10^7^ and 7.0 × 10^10^ M^− 1^, respectively.

The observation of sulfatase-catalyzed hydrolysis of phosphate mono- (**2a**) and diesters (**3b**) and phosphonate monoesters (**4b**) reinforces the idea of “crosswise” catalytic promiscuity typical of the sulfatases of the AP superfamily [26], [41], [46]. The observed ratio of *k*_cat_/*K*_M_ values for the *Sm*CS-catalyzed arylsulfate hydrolysis and the promiscuous phosphodiester and phosphonate monoester reactions matched those observed previously [26] (Fig. S15b and c). Based on the previously published correlation for AS-catalyzed sulfate and phosphate monoester hydrolysis [26], and the detection limit for enzymatic hydrolysis of phosphate monoester **2b**, the lack of detectable *Sm*CS-catalyzed hydrolysis of phosphate monoester **2b** is not unexpected (Fig. S15a). The observation of conserved degrees of preference toward arylsulfate over arylphosphoesters confirms once more that *Sm*CS is acting as a typical AS, despite its high specificity toward choline-*O*-sulfate (**1a**).

The emergence of the ability to hydrolyze choline-*O*-sulfate thus far appears to be unique to the CSs described here. Its evolution within the AS/PMH group (Figs. 7b and 8) of the AP superfamily is unexpected, given that these enzymes catalyze S–O cleavage of sulfate esters, whereas for alkylsulfates such as choline-*O*-sulfate **1a**, C–O attack would be expected. A possible factor that drives adaptation toward an alternative mechanism compared to the chemically favored C–O cleavage could be the need to accommodate both binding of the quaternary ammonium as well as efficient catalysis. It is conceivable that these molecular recognition requirements cannot easily be reconciled: the strongly electronegative character typical of a nucleophilic residue or an activated water molecule would be prone to formation of a strong interaction with the positively charged quaternary ammonium present in the substrate, resulting in a non-productive substrate binding mode. In order to prevent the latter, an additional strongly electronegative residue would have to be present in the active site. Both strongly electronegative residues or groups would benefit from some distance between them to avoid strong coulombic repulsion within the active site. In each case, efficient catalysis would be precluded. By employing S–O attack, both the issues raised here are of lesser importance: (i) the fGly nucleophile is surrounded by positive charges, which prevents non-productive interaction of the nucleophile with the quaternary ammonium group, and (ii) the nucleophile for S–O attack can simply be further removed from the negatively charged residue that interacts with the quaternary ammonium group, limiting possible coulombic repulsion as compared to a nucleophile that would be well positioned for C–O attack.

## Conclusions

The biochemical characterization and crystal structure of the CS from *S. meliloti* (*Sm*CS) allows rationalization of its catalytic properties. Despite its specificity for a primary alkyl sulfate, a substrate expected to be converted by a class III sulfatase *via* C–O attack (Fig. 2), the enzyme was shown to hydrolyze choline-*O*-sulfate *via* S–O attack. The protein structure confirms its previously postulated similarity to ASs of the AP superfamily [23]: the enzyme is active as a tetramer and its oligomerization is mediated by the C-terminal tail. The latter is essential for the catalytic activity of *Sm*CS, in particular for its ability to catalyze the hydrolysis of choline-*O*-sulfate. The deeply buried, L-shaped active site is in fact largely formed at the interface between two interacting protomers. The relatedness to the AP superfamily is further confirmed by the fact that *Sm*CS uses the same reaction pathway as the AP superfamily type sulfatases. The substrate specificity profile toward 4-nitrophenyl sulfo- and phosphoesters is also consistent with typical AS behavior. We observed that the specificity toward choline-*O*-sulfate can be largely attributed to the interaction of a negatively charged glutamate (Glu386) with the positively charged quaternary ammonium in the choline leaving group. A subsequent multiple-sequence alignment including > 80 (putative) CSs showed us that this glutamate residue is fully conserved among CSs. The combination of its phylogenetic and mechanistic similarity to known ASs and its promiscuity profile all suggest that CS is a typical class I sulfatase that is specific toward a primary alkyl sulfate. The latter suggest that the classification of sulfatases according to the mechanism of the reaction they preferably catalyze is not necessarily an indicator of which substrate types they convert. In particular for alkyl sulfates, we have shown here and in a previous study [36] that class I sulfatases can convert alkyl sulfates *via* the disfavored S–O attack over C–O attack using essentially the same mechanism as for the catalysis of aryl sulfate hydrolysis.

## Materials and Methods

### Materials

Choline, sulfate monoester **1b,** phosphate monoesters **2a** and **2b** were purchased from Sigma. Sulfate monoesters **1a** [82], **1d**–**1h** [49], [68], phosphate diester **3b** [83] and phosphonate monoester **4b** [26] were synthesized as described previously. Sulfate monoester **1c** was synthesized using a similar procedure to that described for alkyl sulfates **1d**–**1h** [68]. Details on characterization are listed in the supporting information (Fig. S16). Alkylsulfatases Pisa1 [49] and SdsA1 [35], ASs PAS [46], *Sp*AS1 [26] and *Sp*AS2 [26], and *Rl*PMH [52] were prepared as described previously. The choline detection kit was purchased from Abcam. All restriction enzymes and T4 DNA ligase were from Fermentas. Vector pASK-IBA5plus and strep-tactin resin were purchased from Stratech Scientific. *Pfu* turbo was from Agilent.

### Cloning and mutagenesis

The gene encoding CS from *S. meliloti* 1021 (Uniprot accession number protein sequence: O69787, gene sequence positions 1491–3029 of GenBank accession number U39940) was amplified by PCR using the appropriate forward and reverse cloning primers with commercially available genomic DNA from *S. meliloti* 1021 (ATCC 51124D-5, LGC promochem) as a template (Table S15). Primers were used at 0.4 nM in a reaction with 0.2 mM dNTPs and 0.05 U μL^− 1^ *Pfu*-Turbo® DNA polymerase. The temperature program used was 15 min at 95 °C without polymerase, followed by 30 cycles of 60 s 95 °C, 45 s 68 °C–0.5 °C per cycle (each cycle the temperature of this segment was lowered by 0.5 °C), 240 s at 72 °C, and finished with 10 min at 72 °C. The PCR product was digested with *BamH*I and *Hind*III restriction endonucleases and subsequently ligated into *BamH*I–*Hind*III digested pASK-IBA5plus plasmid DNA using T4 DNA ligase. The ligation mixture was transformed into *E. coli* TOP10 by electroporation. The resulting transformants were plated on LB medium containing ampicillin (100 mg L^− 1^). Colonies were checked for insert using a PCR reaction with *Taq* polymerase and colony material as the template. Positive colonies were used to inoculate 5 mL of liquid LB medium containing ampicillin (100 mg L^− 1^) and grown overnight at 37 °C. Plasmid DNA was extracted and the insert was sequenced using pASK-IBA5plus sequencing primers.

Site-directed mutants of *Sm*CS were constructed using the QuikChange method (Agilent) using the primers listed in Table S15 with pASK-IBA5plus*Sm*CS WT plasmid as a template.

### Protein production and purification

Plasmids encoding Strep-tagged *Sm*CS mutants C54A and C54S were produced in *E. coli* TOP 10. All other *Sm*CS-encoding genes were expressed in *E. coli* BL21(DE3) expressing *Mtb*FGE [53] from the pRSFDuet*Mtb*FGE plasmid as described previously for several other fGly-containing enzymes [26], [47], [52]. Expression of *Sm*CS from the pASK-IBA5plus vector results in a translational fusion with an N-terminal Strep-Tag. Mutants *Sm*CS C54A and C54S were produced by growing cells to OD_600_ ~ 0.5 at 37 °C in 2 × YT medium containing ampicillin (100 mg L^− 1^), lowering the temperature to 28 °C, inducing expression of the pASK-IBA5plus constructs by the addition of anhydrotetracycline (200 μg L^− 1^) followed by overnight growth at 28 °C. All other variants were expressed by growing cells to OD_600_ ~ 0.5 in 2 × YT medium with ampicillin (100 mg L^− 1^) and kanamycin (50 mg L^− 1^), lowering the temperature to 28 °C, inducing expression of *Mtb*FGE by addition of IPTG (1 mM) ~ 30 min prior to inducing expression of the pASK-IBA5plus construct by addition of anhydrotetracycline (200 μg L^− 1^) followed by overnight growth at 28 °C.

All overnight cultures were harvested by centrifugation and resuspended in 50 mM Tris–HCl (pH 8.0). The cells were lysed with an Emulsiflex C5-homogenizer (Avestin) and cell-free extract (CFE) was obtained by centrifugation at 30,000*g* for 90 min. CFE was loaded onto a Q-Sepharose anion exchange column calibrated in 50 mM Tris–HCl (pH 8.0). The column was washed with 2 column volumes 50 mM Tris–HCl (pH 8.0) and protein was eluted with a gradient of 0–1 M NaCl in 50 mM Tris–HCl (pH 8.0) over 12 column volumes. Protein containing fractions were tested for activity toward sulfate monoester **1b**. Fractions containing active protein were pooled and 1/10 of the pooled volume of 1 M Tris–HCl (pH 8.0) + 1.5 M NaCl was added. An appropriate amount of the pooled protein was subsequently loaded onto 1-mL Strep-Tactin column equilibrated with 100 mM Tris–HCl (pH 8.0) + 150 mM NaCl. The column was washed with 100 mM Tris–HCl (pH 8.0) + 150 mM NaCl to remove unbound protein and the tagged proteins were eluted with 2.5 mM *d*-desthiobiotin in 100 mM Tris–HCl (pH 8.0) + 150 mM NaCl. The active protein-containing fractions were pooled and concentrated to 10–15 mg mL^− 1^ protein and loaded onto a HiLoad 16/600 Superdex 200 prep grade size exclusion column [running in 100 mM Tris–HCl (pH 8.0), 150 mM NaCl]. Active protein eluted at a molecular mass of ~ 240 kDa, corresponding to a tetramer. Protein containing fractions were concentrated to 100–200 μM, divided into the appropriate aliquots, flash frozen in liquid N_2_ and stored at − 20 °C.

### Enzyme assays

Initial rates (*V*_obs_) for the hydrolysis of **1b-4b** were determined by following the 4-nitrophenol formation at 400 nm in a SpectraMax Plus microtiter plate reader at substrate concentrations ranging 4–75 mM in 100 mM imidazole–HCl (pH 6.0–7.2), Tris–HCl (pH 7.2–8.8) or glycine–NaOH (8.8–10.0). Typical enzyme concentrations used were 0.15–0.60 μM for sulfate monoester **1b** and phosphonate monoester **4b** and 2–20 μM for phosphate esters **2b** and **3b** for *Sm*CS WT. For the *Sm*CS mutants, the typical enzyme concentrations were 1–20 μM. All substrate concentrations were determined more precisely by performing full turnover of the substrate stock solutions by adding excess PAS [46] (**1b**), AP (Sigma, cat no. P7923, **2b**) or *Bc*PMH [47] (**3b** and **4b**) and measuring the concentration of 4-nitrophenol formed. Catalytic parameters *k*_cat_, *K*_M_ and/or *k*_cat_/*K*_M_ were obtained from fitting the dependency of *V*_obs_ on substrate concentration ([S]) at constant enzyme concentration ([Enz]). In cases where *V*_obs_ showed saturation at higher substrate conctrations, the data were fitted to Eq. (1). In cases where the dependence of *V*_obs_ on substrate concentration was linear, the data were fitted to Eq. (2). In the latter, *k*_cat_/*K*_M_ was treated as a single parameter.

(1) $$V_{\mathrm{obs}}=\frac{k_{\mathrm{cat}}\times \left[\mathrm{Enz}\right]\times \left[S\right]}{K_{M}+\left[S\right]}$$

(2) $$V_{\mathrm{obs}}=\frac{k_{\mathrm{cat}}}{K_{M}}\times \left[\mathrm{Enz}\right]\times \left[S\right]$$

Activity toward choline-*O*-sulfate **1a** and phosphoryl choline **2a** was determined by monitoring the choline formation over time using the choline/acetylcholine assay kit (Abcam, cat. no. ab65345). For *Sm*CS WT, typically 0.9–1.5 μM enzyme was added to 1 mM choline-*O*-sulfate (**1a**) in 100 mM imidazole–HCl (pH 6.8–7.2) or Tris–HCl (pH 7.2–8.8) and the reaction was left to proceed at 25 °C. Samples of 50 μL were flash frozen in liquid N_2_ at various time points. Prior to choline detection, the flash-frozen samples were quickly heated to 95 °C to inactivate the enzyme. These samples were diluted 10 times and to 50 μL diluted sample 50 μL choline assay mix was added (choline assay mix was made according to the manufacturers' instructions). The samples were incubated at room temperature in a dark place for 30 min and absorption was measured at 570 nm. If total turnover of substrate (starting at concentration [S] = [S]_*t* = 0_) was achieved, the total progress curve of product formation ([P]) *versus* time could be numerically fitted to Eqs. (3), (5) using Micromath Scientist™ to obtain *k*_cat_, and *K*_M_, provided that at the start of the reaction, the substrate concentration was above the *K*_M_. The reaction product choline can act as a competitive inhibitor, albeit a relatively weak one (*K*_IC_ ~ 52–6.3 mM, depending on pH; Fig. S11). When fitting Eqs. (3), (5) to progress curves of *Sm*CS-catalyzed conversion choline-*O*-sulfate **1a**, the concentration of the choline product increases over time, which can cause inhibition at higher choline concentrations. The time-dependent increase in the choline concentration cannot be easily accounted for in the fitting procedure. This possible problem was circumvented by recording the progress curves starting at a low substrate concentration, in which case the maximum concentration of choline product (1 mM) would cause at most 10% reduction in observed rate, which falls within experimental and fitting error. Since 1 mM is not exceeding *K*_M_ > 10-fold, we determined an accurate value for *k*_cat_ by measuring initial rates of *Sm*CS-catalyzed choline production at 10 and 20 mM choline-*O*-sulfate **1a**. These intial rates which were used as proxy to calculate *k*_cat_ (provided they were identical withinin error). This value was subsequently fixed while fitting a product formation progress curve starting from 1 mM choline-*O*-sulfate **1a** (i.e., [S]_*t* = 0_ = 1 mM) to Eqs. (3), (5). An example fit is shown in Fig. S16. If substrate saturation could not be achieved and [S]_*t* = 0_ was well below the *K*_M_, the data could be fitted to Eqs. (4), (5) with *k*_cat_/*K*_M_ treated as a single parameter.

(3) $$\frac{d\left[S\right]}{\mathrm{dt}}=-\frac{k_{\mathrm{cat}}\times \left[\mathrm{Enz}\right]\times \left[S\right]}{K_{M}+\left[S\right]}$$

(4) $$\frac{d\left[S\right]}{\mathrm{dt}}=-\frac{k_{\mathrm{cat}}}{K_{M}}\times \left[\mathrm{Enz}\right]\times \left[S\right]$$

(5) $$\left[P\right]={\left[S\right]}_{t=0}-\left[S\right]$$

Kinetic measurements of active site mutants were performed with 1 mM choline-*O*-sulfate (**1a**) in 100 mM Tris–HCl (pH 7.6) and 20–30 μM *Sm*CS. If the activity was too low to achieve total turnover within ~ 5 days, a lower limit for activity could be derived from the initial rate of product formation. The data for phosphoryl choline were obtained by monitoring the total turnover of 1 mM phosphate monoester **2a** in 100 mM Tris–HCl (pH 7.6) catalyzed by 33 μM *Sm*CS WT.

The errors for the individual parameters shown in Table 1, Table 2 represet those arising from the fitting procedure used. In cases where *k*_cat_/*K*_M_ was calculated from values for *k*_cat_ and *K*_M_ (as obtained from fitting data to Eqs. (1), (3)), the error (*δ*) for *k*_cat_/*K*_M_ was calculated according to Eq. (6).

(6) $$\delta \frac{k_{\mathrm{cat}}}{K_{M}}=|\frac{k_{\mathrm{cat}}}{K_{M}}|\times \sqrt{{\left(\frac{\delta k_{\mathrm{cat}}}{k_{\mathrm{cat}}}\right)}^{2}+{\left(\frac{\delta K_{M}}{K_{M}}\right)}^{2}}$$

Alkylsulfatases Pisa1 and SdsA1, ASs PAS, *Sp*AS1 and *Sp*AS2 and PMH *Rl*PMH were tested for choline-*O*-sulfatase activity by incubating 2–4 μM enzyme with 1 mM sulfate monoester **1a** in 100 mM Tris–HCl (pH 7.6) at 25 °C for 24 h, followed by choline detection as described above.

*Sm*CS was tested for activity toward alkyl sulfates **1c**–**1h** essentially as described previously for Pisa1 [49]. Typical incubations were done with 1.8 μM of enzyme and alkyl sulfate (4 mg mL^− 1^) in 100 mM Tris–HCl (pH 8.0). The mixtures were incubated for 24 h at 30 °C while shaking at 120 rpm. The alcohol product was extracted with ethyl acetate (1:1 with the aqueous phase) and the organic phase was dried over anhydrous sodium sulfate and subsequently derivatized to form the acetate ester by adding DMAP and acetic anhydride. The derivatized product was analyzed by chiral GC-FID [Thermo Finnigan FOCUS GC, Varian Chirasil Dex CB column (25 m × 0.32 mm × 0.25 μm film)] with the following temperature program: injector temperature, 200 °C; flow, 1.3 mL min^− 1^; temperature program, 80 °C; and hold for 1.0 min, 15 °C min^− 1^, to 110 °C, 4 °C min^− 1^, to 130 °C, 10 °C min^− 1^, to 180 °C.

Given that the fGly modification is incomplete (see above), it is possible that the kinetic data reported underestimate the true rates by a factor of ~ 2. The strong effect of the Cys to Ser mutation (~ 1000-fold reduction in *k*_cat_/*K*_M_) on the conversion of sulfate monoesters **1a** and **1b** suggests that the activity of the Cys form (which is likely to be similar to the serine mutant) would contribute very little to the observed activity. This means that the comparison between the different substrates is valid.

### Crystallization and structure determination

Following high-throughput evaluation of sparse matrix crystallization conditions in sitting drops in 96-well plates, crystals of *Sm*CS were observed in several conditions after several days of incubation at 20 °C. After extensive screening and optimization, the best crystals (with dimensions of 600 × 400 × 200 μm) were obtained in 800 mM sodium citrate and 100 mM imidazole (pH 8.0) using hanging drop vapor diffusion with equal volumes of protein containing solution (11.2 mg mL^− 1^) and reservoir solution (3 μL total start volume). For cryoprotection prior to flash-cooling in liquid nitrogen, the crystals were briefly transferred to a drop containing the crystallization solution supplemented with 20% (v/v) glycerol.

Diffraction data were collected on the ID23-2 beamline at the European Synchrotron Radiation Facility, Grenoble, France. Data were processed and reduced using *XDS* [84] and *AIMLESS* [85] using [I]/[*σ*I] cutoff of 2 to define the high resolution cutoff, while ensuring completeness of the data. The phases were obtained by molecular replacement with energy- and electron density-guided model building and refinement *MR-ROSETTA* [86] protocols as implemented in the *PHENIX* suite [87], using a search model based on the coordinates of *Rl*PMH (PDB ID 2VQR). Model improvement was monitored through *R*_free_ during rounds of density modification and reciprocal-space refinement. After extensive manual re-building in *COOT* [88], combined with maximum-likelihood-based restrained refinement in *BUSTER-TNT* [89] and phenix.refine [87], the final model was refined to 2.8-Å resolution and had *R*_work_/*R*_free_ values of 0.205/0.253 (Table S4). The stereochemistry of the structure was assessed and validated with *MOLPROBITY* [90]. The metal ion in the structure was assigned as Ca^2 +^, based on microPIXE experiement and the active site residue 54 modeled as a mixture of formyl glycine and cysteine, both at half occupancies. The structure and corresponding structure factors have been submitted to PDB with accession code 6FNY.

### Molecular mass determination by combined size exclusion chromatography–multi-angle laser light scattering

The oligomeric state of wild-type *Sm*CS and the two truncation mutants Δ12 and Δ23 were determined by loading 100 μL of 2 mg mL^− 1^ protein on a Superdex 200 10/300 HR size exclusion chromatography column running in 100 mM Tris–HCl (pH 8.0) and 150 mM NaCl (0.5 mL min^− 1^) coupled to a Wyatt Dawn multi-angle light scattering detector and a Wyatt T-Rex differential refractive index detector.

### Comparison of *Sm*CS with other AP superfamily members

All structural homology searches and subsequent structural alignments were performed using the PDBeFold server [91] (http://www.ebi.ac.uk/msd-srv/ssm/cgi-bin/ssmserver). The conserved putative active site residues were assigned based on homology with *Sp*AS1 [26] (PDB: 4UPI) and *Rl*PMH [52] (2VQR). The other active site residues were found using the HotSpot Wizard (loschmidt.chemi.muni.cz/hotspotwizard) [92] and manual inspection of the 3D structure.

In order to obtain an indication as to the phylogenetic relationship between *Sm*CS and all other AP-type ASs and PMHs, we aligned *Sm*CS with all 17 ASs/PMHs of known structure using Secondary Structure Matching [91] (see Table S11 for more details on the multiple structural alignment). The 267 positions that aligned structurally for all 18 enzymes (magenta regions in Fig. 7a) were used to build a phylogenetic tree (Fig. 7b) using RAxML HPC2 8.0.24 [93] at the XSEDE sever of the CIPRES Science Gateway [94] (http://www.phylo.org/portal2). We used the “Le and Gascuel” [95] amino acid replacement rate matrix with four category gamma rates, estimated proportion of invariable sites and empirical base frequencies. The optimal tree-building parameters and substitution matrix were calculated from the sequence alignment using ProtTest 2.4 [96].

The phylogenetic relationship between CSs, the dimeric ASs and PMHs was built from a multiple-sequence alignment of 87 (putative) CSs, 60 from α-proteobacteria and 27 from β-proteobacteria (Table S14), with all the sequences included in the previously reported phylogenetic relationship between the PMHs and ASs [26] (Tables S12 and S13, respectively). The multiple-sequence alignment was generated using the 3D coffee mode of the T-coffee multiple-sequence alignment package [97]. The maximum likelihood phylogenetic tree was calculated using RAxML HPC2 8.0.24 [93] at the XSEDE sever of the CIPRES Science Gateway [94] (http://www.phylo.org/portal2) using the “Le and Gascuel” [95] amino acid replacement rate matrix with four category gamma rates, estimated proportion of invariable sites and empirical base frequencies. The optimal tree-building parameters and substitution matrix were calculated from the sequence alignment using ProtTest 3.2 [96].

### Accession number

The coordinates and the structure factors have been deposited in the Protein Data Bank under accession number 6FNY.

## Appendix A Supplementary data

Supplementary material 1 Image 1

Supplementary material 2 Image 2

Supplementary material 3 Image 3

Supplementary material 4 Image 4
