# Supplementary material for: Structural and Mechanistic Analysis of the Choline Sulfatase from Sinorhizobium melliloti: A Class I Sulfatase Specific for an Alkyl Sulfate Ester
Source: J Mol Biol. 2018 Mar 30;430(7):1004–23. doi: 10.1016/j.jmb.2018.02.010 (PMC5870055; doi:10.1016/j.jmb.2018.02.010)
Supplement: Supplementary file 1 — Supplementary material 1 [file mmc1.pdf]

Supporting information for

Structural and Mechanistic Analysis of the Choline Sulfatase from *Sinorhizobium melliloti*: a Class I Sulfatase Specific for an Alkyl Sulfate Ester

Bert van Loo, Markus Schober, Eugene Valkov, Magdalena Heberlein, Erich Bornberg-Bauer, Kurt Faber, Marko Hyvönen and Florian Hollfelder

Table of Contents

Calculation of rate accelerations and 'kinetic penalties' for choline-*O*-sulfate hydrolysis 3

Detection limits for catalytic parameters 4

4-Nitrophenyl sulfo/phosphoesters **1b**-**4b** 4

Choline sulfo/phosphoesters **1a** and **2a** 5

Alkyl sulfates **1c**-**1h**....5

Comparison of the kinetic parameters reported in this study with those reported by Sanchez-Romero & Olguin (2015) [3] 7

Structural comparison of *Sm*CS with all AP-type ASs and PMHs 8

Supporting Figures....9

Fig. S1: pH-rate profile of *Sm*CS towards choline-*O*-sulfate (**1a**, closed circles) and 4-nitrophenyl sulfate (**1b**, open circles). 9

Fig. S2: Alkyl sulfates tested for activity with *Sm*CS. 10

Fig. S3. Interface positioning in choline sulfatase, PMHs and dimeric ASs. 11

Fig. S4. Multiple sequence alignment data of the structural alignment of several AP-superfamily sulfatases: choline sulfatase (CS), dimeric arylsulfatases (ASs)) [9] and phosphonate monoester hydrolases (PMHs) [9-11]. 13

Fig. S5: Elution patterns for *Sm*CS WT (black), Δ12 (red) and Δ23 (blue) on a Superdex 200 size-exclusion column....14

Fig. S6: Stimulation of *Sm*CS WT-catalyzed hydrolysis of 4-nitrophenyl sulfate (sulfate monoester **1b**) by various divalent metal ions....15

Fig. S7: Electron density map for the active site nucleophile in *Sm*CS. 16

Fig. S8. Logo representation of the multiple sequence alignment of 87 different choline sulfatases. 17

Fig. S9. Active site geometry and conservation of *Rl*PMH. 18

Fig. S10. ESI-MS spectrum for choline (*m/z* 104.1073, highlighted) resulting from the *Sm*CS-catalyzed hydrolysis of 5 mM choline-*O*-sulfate (**1a**) in the presence of 50% (v/v) $H_2{}^{18}O$ in 20 mM Tris-HCl pH 7.6. 19

Fig. S11. Inhibition of *Sm*CS-catalyzed hydrolysis of 4-nitrophenyl sulfate (**1b**) by choline. 20

Fig. S12. PMH clade from Fig. 8 displayed in full detail and with labelled taxa. 21

Fig. S13. AS clade from Fig. 8 displayed in full detail and with labelled taxa. 22

Fig. S14. CS clade from Fig. 8 displayed in full detail and with labelled taxa. 23

Fig. S15. Correlation of primary and promiscuous activities for sulfatases. 24

Fig S16. ^{1}H-NMR and ^{13}C-NMR spectra for 3,3-dimethylbutyl sulfate (**1c**). 25

Fig S17. Example fit for determining choline sulfatase activity. 26

Supporting Tables 27

Table S1. Kinetic data for the pH rate profile of *Sm*CS wild type for 4-nitrophenyl sulfate **1b** and choline-*O*-sulfate **1a**. 27

Table S2. First (k_1) and second (k_2) order rate constants for uncatalyzed hydrolysis of sulfoesters an phosphoesters used in this study (Temperature = 25 ^{o}C). 28

Table S3. Comparison kinetic data from Sanchez-Romero & Olguin (2015) [3] with the data from our study. 29

Table S4. Crystallographic data collection and refinement statistics. The coordinates and the structure factors have been submitted to Protein Data Bank (PDB) with accession code 6FNY. 30

Table S5. PDBePISA oligomerization analysis [19] (http://www.ebi.ac.uk/pdbe/pisa/) of *Sm*CS. 31

Table S6. All AP-superfamily type sulfatases and phosphonate monoester hydrolases of known structure. 32

Table S7. Putative active site residues[a] for *Sm*CS, arylsulfatases (ASs) and phosphonate monoester hydrolases (PMHs) of known structure. 33

Table S8. Metal occupancy according to MicroPIXE measurements 34

Table S9. Conserved active pocket (P) and access tunnel (T) residues for choline sulfatases[a]. 35

Table S10. Secondary Structure Matching (SSM)[a] pairwise alignment data for *Sm*CS with all AP-superfamily like ASs and PMHs of known structure and with *E. coli* alkaline phosphatase (*Ec*AP) and *Xanthomonas axonopodis* nucleotide phosphodiesterase (*Xa*NPP). 36

Table S11. Secondary Structure Matching (SSM)[a] data for the multiple structural alignment data for *Sm*CS with all AP-superfamily-like ASs and PMHs of known structure. 37

Table S12. (Putative) phosphonate monoester hydrolases (PMHs) included in the alignment on which phylogenetic trees in Fig. 8 and Fig. S12 are based 38

Table S13. (Putative) arylsulfatases (ASs) included in the alignment on which phylogenetic trees in Fig. 8 and Fig. S13 are based. 43

Table S14. (Putative) choline sulfatases included in the alignment on which phylogenetic trees in Fig. 8 and Fig. S14 are based. 48

Table S15. Primers used for cloning and site-directed mutagenesis 53

Supporting references 54

Calculation of rate accelerations and 'kinetic penalties' for choline-*O*-sulfate hydrolysis

The activation energies for hydrolysis of choline-*O*-sulfate (sulfate monoester **1a**) via attack on the carbon (C-O_{attack}) and the sulfur (S-O_{attack}) center differ considerably as exemplified by their respective rate constants in aqueous solution (k_{uncat} values):

$k_{uncat}^{C\text{-}Oattack}$ = 6.8 × 10^{-12} s^{-1} (assumed to be approximately equal to the rate constant for pentyl sulfate hydrolysis [1])

$k_{uncat}^{S\text{-}Oattack}$ = $10^{(-1.81 \times 13.9 + 3.6)}$ = 2.8 × 10^{-22} s^{-1}(using the published linear free energy relationship [1] and a pK_a of 13.9 for the hydroxyl group in choline [2]).

The difference in Gibbs energy of activation (ΔΔG‡) between these two reaction paths is essentially the kinetic penalty the enzyme pays for using the path that requires a higher activation energy and can be calculated by:

$$\Delta\Delta G^{\ddagger} = R \times T \times \ln[\frac{k_{uncat}^{C-Oattack}}{k_{uncat}^{S-Oattack}}] = 1.987 \times (25 + 273) \times \ln[\frac{6.8 \times 10^{-12}}{2.8 \times 10^{-22}}]$$
$$= 1.42 \times 10^{4}\ cal\ mol^{-1}$$

Therefore, the kinetic penalty for following the catalytic pathway *via* S-O_{attack} over C-O_{attack} is 14.2 kcal mol^{-1}.

Detection limits for catalytic parameters

The identification of potentially weak catalysis e.g. low levels of promiscuous activity or compromised catalysis as a result of deleterious mutations, depends on whether very slow enzymatic rates can be reliably measured. The following paragraphs describe the dynamic range that could be accessed in our experiments.

4-Nitrophenyl sulfo/phosphoesters **1b-4b**

In the presence of *Sm*CS ([Enz] = 20 μM = 2 × 10^{-5} M) and assuming that a 2-fold enhancement of the observed rate (V_{obs} – i.e. the sum of the enzymatic rate (V_{Enz}) and rate of uncatalyzed hydrolysis (V_{uncat})) over the V_{uncat} is necessary to reliably detect catalysis one can define the following equations:

$$V_{obs} = V_{Enz} + V_{uncat}$$

$$\frac{V_{obs}}{V_{uncat}} \geq 2$$

$$\frac{V_{Enz}}{V_{uncat}} \geq 1$$

$$\frac{V_{Enz}}{V_{uncat}} = \frac{\frac{k_{cat}}{K_M} \times [Enz] \times [S]}{k_{uncat} \times [S]} = \frac{\frac{k_{cat}}{K_M} \times [Enz]}{k_{uncat}} = \frac{\frac{k_{cat}}{K_M} \times 2 \times 10^{-5}}{k_{uncat}} \geq 1$$

$$\frac{k_{cat}}{K_M} \geq k_{uncat} \times 5 \times 10^4$$

in which the uncatalyzed rate constant (k_{uncat}) is the sum of all background rates in an aqueous solution at the given pH.

For sulfate monoester **1b** and phosphoesters **3b** and **4b**, k_{uncat} is equal to the first order chemical rate constant k_1 as listed in Table S2. For phosphate monoester **2b** k_{uncat} is the sum of $k_1^{monoanion}$ and $k_1^{dianion}$ (see Table S2 for details), both corrected for the fraction of mono- and dianion present at a given pH (pH 6.0 in this particular case). The fractions of mono- and dianion are based on equation the Henderson-Hasselbalch equation, assuming a pK_a of 5 for the equilibrium between phosphate monoester mono- (acid) and dianions (base).

Choline sulfo/phosphoesters **1a** and **2a**

In principle, the equations for the hydrolysis of 4-nitrophenyl sulfo/phosphoesters described above apply to the conversion of choline-*O*-sulfate (**1a**) and phosphorylcholine (**2a**). However, the sensitivity of the choline detection assay and stability of the enzyme are limiting rather than the minimum required rate-enhancement over background. For example, the k_{uncat} for choline-*O*-sulfate at near neutral pH is expected to be approximately equal to the k_{uncat} for *n*-pentyl sulfate, which is 6.8×10^{-12} s^{-1} [1], assuming that attack at the carbon proceeds with similar rates in both these alkyl sulfates (because sulfate is the leaving group in both cases). It is possible that this comparison underestimates k_{uncat} somewhat, because the quaternary ammonium group is likely to provide some activation of the carbon center due to its electron withdrawing effect. However, due to this effect being remote (connected via three carbon centers, without resonance), it is not expected to be large. Based on this k_{uncat} and the equations listed above, a k_{cat}/K_M of 3.4×10^{-7} s^{-1} M^{-1} for choline-*O*-sulfate should still be measurable. In order for the reaction product to be detected, a minimum of 0.01 nmol choline in 50 μl sample is required (= 0.2 μM), according to the specifications listed for the Abcam Choline/Acetylcholine assay kit (cat. no. ab65345, http://www.abcam.com/cholineacetylcholine-assay-kit-ab65345.html). The measurement of enzymatic activity is most sensitive when the enzyme is operating under pseudo-first order conditions (i.e. $V_{Enz} = k_{cat}/K_M \times$ [Enzyme] $\times$ [Choline ester]), since both enzymatic and background rate increases linearly with the substrate concentration (in contrast to when the enzyme is under saturating conditions, in which case the enzymatic rate is constant while the background rate increases) The enzyme-catalyzed choline-*O*-sulfate hydrolysis with a k_{cat}/K_M of 3.4×10^{-7} s^{-1} M^{-1} would need to proceed for two days at 25 °C in order to reach the detection limit, provided the enzyme has a K_M > 1 M and the substrate concentration is 100 mM. In practice at least three data points are necessary for rate measurements, i.e. 3 × 0.2 μM = 0.6 μM product are to be formed at the end of the reaction. In our hands the enzyme appears to be stable for ~5 days at room temperature. However, in practice the reaction will be carried out at a substrate concentration of 1 mM, due the fact that K_M is expected to be around 10 mM at the most. At 1 mM substrate concentration and 20 μM enzyme the required k_{cat}/K_M to result in at least 0.6 μM choline within five days is 7×10^{-5} s^{-1} M^{-1}.

Alkyl sulfates **1c**-**1h**

The final concentration of alkyl sulfates **1c-1h** ranged between 10-24 mM (4 mg mL^{-1}). All incubations contained 2-4 μM enzyme. After 24 hours, no conversion could be detected. Analysis of the sensitivity of our assay suggests that 1% conversion should still be detectable.

Lowest possible concentration of product ([P]) after 1% conversion: 1% of 10 mM = 100 μM

Lowest possible rate assuming linear product formation for 24 hours: 100 μM/(24 × 3600) = 1.6 nM s^{-1} (V_{obs})

Maximal enzyme concentration ([Enz]) used: 4 μM

Minimal substrate concentration ([S]): 10 mM

Assuming that the enzyme is operating under sub-saturating conditions ([S] <<< K_M), we can state that:

$$V_{obs} = \frac{k_{cat}}{K_M} \times [Enz] \times [S]$$

in which case the lowest possible catalytic efficiency (k_{cat}/K_M) that could still have been detected is:

$$\frac{k_{cat}}{K_M} = \frac{V_{obs}}{[Enz] \times [S]} = \frac{1.6 \times 10^{-9}}{4 \times 10^{-6} \times 10 \times 10^{-3}} = 4 \times 10^{-2}\ s^{-1}M^{-1}$$

Comparison of the kinetic parameters reported in this study with those reported by Sanchez-Romero & Olguin (2015) [3]

The previously published characterization of *Sm*CS [3] states that the enzyme is a dimer in solution (while our data suggests that the enzyme is a tetramer), and has significantly lowered catalytic efficiencies compared to the ones reported in our study (Table S3). Part of the difference in catalytic efficiencies can be attributed to the fact that we, in contrast to Sanchez-Romero & Olguin, co-expressed the fGly-generating enzyme from *Mycobacterium tuberculosis* H37v (*Mtb*FGE) with *Sm*CS. Sanchez-Romero & Olguin indeed suggested that the moderate activity reported in their work was due to lack of modification of Cys54 into fGly. This does, however, not explain why the difference in catalytic efficiencies between our study and theirs is not identical for choline-*O*-sulfate **1a** (2.1×10^2-fold difference) and 4-nitrophenyl sulfate **1b** (7.4-fold difference). Sanchez-Romero & Olguin expressed *Sm*CS with a C-terminal His-tag, and our data show that the C-terminal region plays an important role in oligomerization (Fig. S5). Furthermore, partial removal of the C-terminal region lowers the catalytic efficiency and this effect is stronger for choline-*O*-sulfate **1a** compared to 4-nitrophenyl sulfate **1b** (Table 2). The N-terminal strep-tactin tag introduced by us is not visible in the X-ray structure, which suggest that it is highly flexible and unlikely to have a large effect on catalysis. Sanchez-Romero & Olguin also report experiments that suggest that the enzyme contains a disulfide bridge. However, in our X-ray structure none of the six cysteines are within 10 Å of each other and also not within 10 Å of *any* residue on other protomers. All this suggest that the C-terminal His-tag used by Sanchez-Romero & Olguin has a significant impact on the overall structure, leading to an underestimation of the enzyme's ability to catalyze the hydrolysis of its native substrate. The fact that the promiscuous phosphoryl choline (**2a**) hydrolysis seen by us and Osteras *et al*. (1998) [4], is not observed by Sanchez-Romero & Olguin, is probably caused by the drastically lowered activity toward choline esters (2.1×10^2-fold for choline-*O*-sulfate **1a**), although we cannot rule out that the calorimetric method used by Sanchez-Romero & Olguin for determination of activity toward choline esters is significantly less sensitive than the choline detection method employed in this present work.

Structural comparison of *Sm*CS with all AP-type ASs and PMHs

In order to place *Sm*CS with the AS/PMH subgroup of the AP-superfamily we initially performed a Secondary Structure Matching (SSM) pairwise comparison [5] [using PDBeFold (http://www.ebi.ac.uk/msd-srv/ssm/cgi-bin/ssmserver)] of the X-ray structure of *Sm*CS with all 13 arylsulfatases (ASs) and 4 phosphonate monoester hydrolases (PMHs) that are members of the AP-superfamily. The various similarity indicators for these enzymes in the pairwise comparison did not show clearly which one of these enzymes with known structures is most similar to *Sm*CS (Table S10). An SSM-based multiple sequence alignment including *Sm*CS and the 17 known AS/PMH structures yielded a similar outcome, although there was a trend toward better scores for r.m.s.d. and sequence identity values for the PMHs and the dimeric ASs from *S. pomeroyi* (in comparison to the pairwise alignments, Table S11). The SSM-based multiple structure alignment showed that 268 positions (~52% of all residues in *Sm*CS, indicated in magenta in Fig. 7a) align structurally for all enzymes included (Cα-alignment). The best alignment is located around the central α/β-fold that is characteristic of the superfamily [6-8]. A phylogenetic tree based on the 277 structurally aligning positions shows that *Sm*CS is most closely related to the two dimeric ASs from *S. pomeroyi* [9] (*Sp*AS1 and *Sp*AS2) and the four PMHs [9-11] (Fig. 7b). This relationship is further emphasized by the observation that several structural elements (indicated in red in Fig. 7a) align only between *Sm*CS and these six enzymes.

Supporting Figures

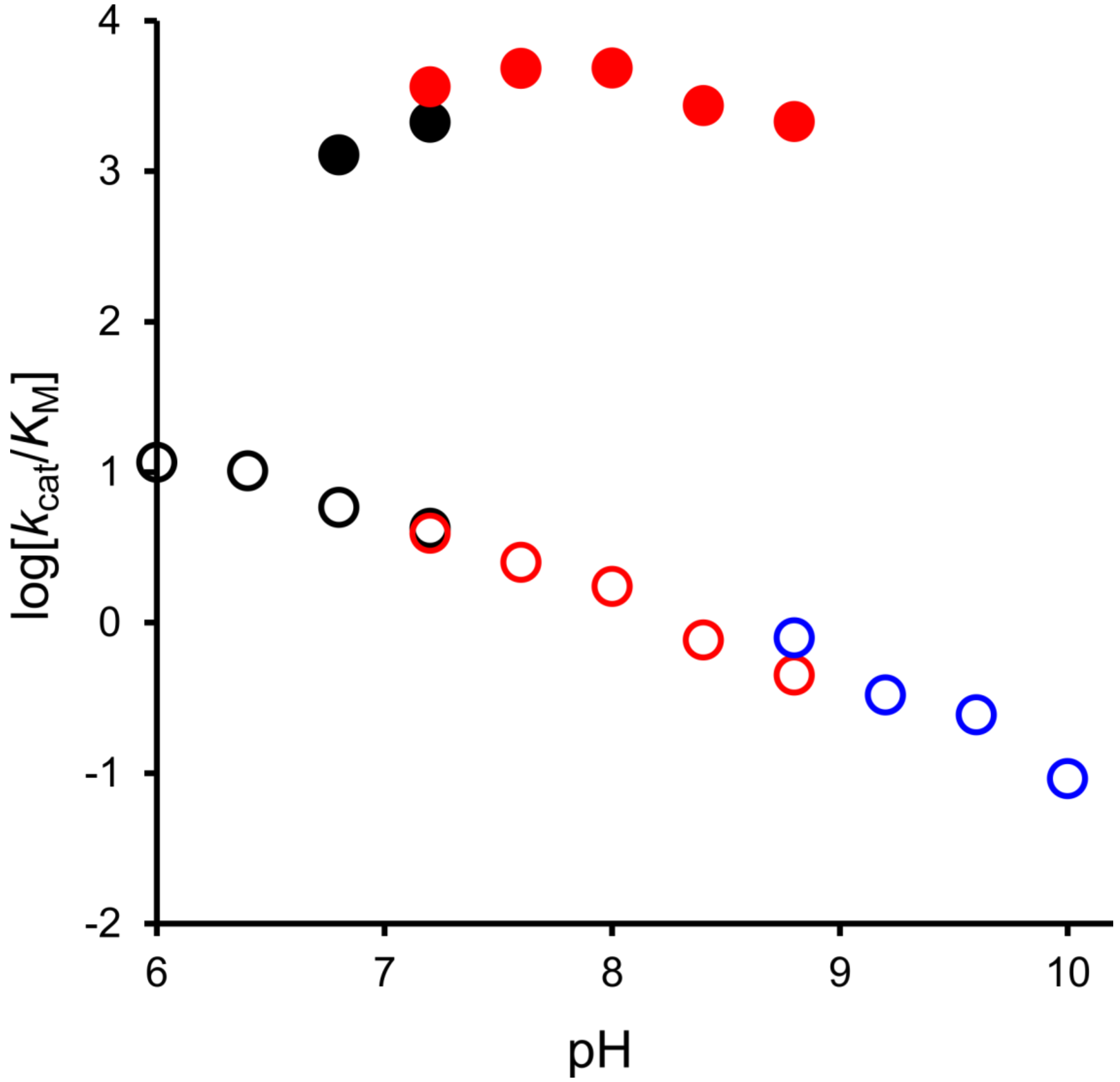

Fig. S1: pH-rate profile of *Sm*CS towards choline-*O*-sulfate (**1a**, closed circles) and 4-nitrophenyl sulfate (**1b**, open circles).

Kinetic data were recorded in 100 mM imidazole (pH 6.0-7.2, black), Tris-HCl (pH 7.2-8.8, red) or glycine-NaOH (pH 8.8-10.0, blue). The data represented in this graph are listed in Table S1.

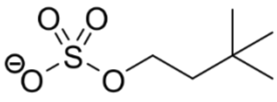

1c

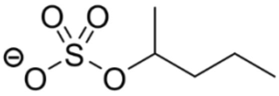

1d

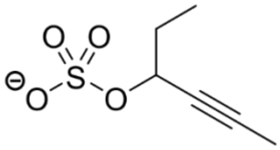

1e

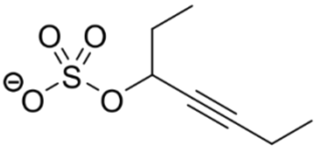

1f

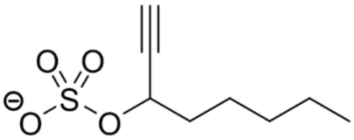

1g

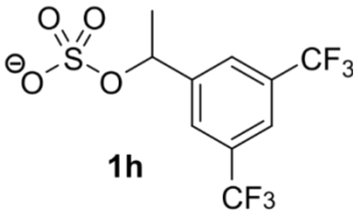

1h

Fig. S2: Alkyl sulfates tested for activity with *Sm*CS.

1c: 3,3-dimethylbutyl sulfate, **1d**: 2-pentyl sulfate, **1e**: 4-hexyn-3-yl sulfate, **1f**: 4-heptyn-3-yl sulfate, **1g:** 1-octyn-3-yl sulfate, **1h:** 1-[3,5-bis(trifluoromethyl)phenyl]ethyl sulfate.

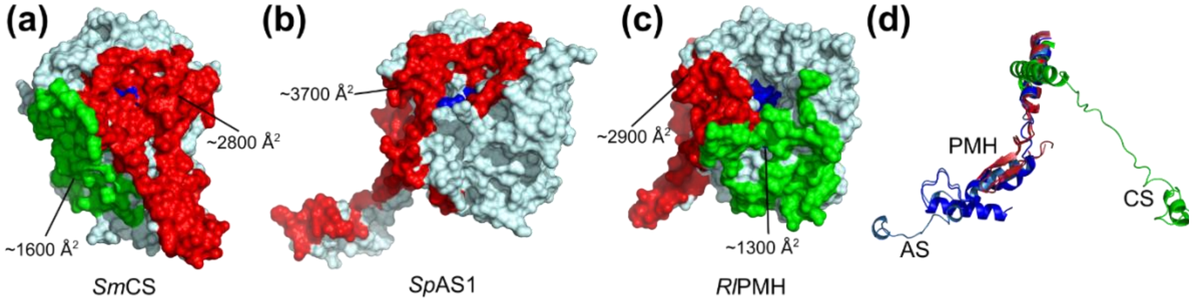

Fig. S3. Interface positioning in choline sulfatase, PMHs and dimeric ASs.

(**a**)-(**c**) Representative protomer structures for all three enzymes classes, with the large (red) and small (green) oligomerization interfaces indicated. The conserved active-site positions are indicated in blue. The displayed orientations are identical for each protomer and result from the multiple structural alignment of *Sm*CS with the four PMHs and two dimeric ASs [9-11]. The red interfaces involve the extreme C-terminus of each of the different protomers. The main difference between *Sm*CS and the other two enzymes lies in the orientation of the two oligomerization interfaces, which has consequences for the oligomeric organization (Fig 4a and b). (**d**) Display of the of only the alignment of the C-terminal region of *Sm*CS (green) with those of the PMHs (shades of red) and the dimeric sulfatases (shades of blue) shows the striking divergence of the extreme C-terminus of *Sm*CS from those of the other two classes of related multimeric enzymes. (Detailed information on the residues that form part of the oligomerization interfaces is indicated in Fig. S4).

```
SmCS     gdpvttGkPNILIIMVDQLNGKLFpdGpA------DFLHAPNLKALAKRSARFHNNYTSS   51
SpAS1    ------N-RNILWIMCDQLRFDYLscY-Gh-----ERLNTPNIDKLAKRGVRFTNAYVQA   50
SpAS2    ----taV-MNILFIMFDQLRWDYLscY-Gh-----KTLNTPHIDRLAAKGVRFDRAYIQS   46
BcPMH    ------R-KNVLLIVVDQWRADFIphL-MraegrePFLKTPNLDRLCREGLTFRNHVTTC   54
RlPMH    ------K-KNVLLIVVDQWRADFVphV-LradgkiDFLKTPNLDRLCREGVTFRNHVTTC   54
ArPMH    ------R-PNVLLISADQWRGDCLsaV-Gh-----ASVKTPNVDALAQDGVLFTRHFAGT   61
SpPMH    ------Q-SNVLFIIIDQLRADCL--W-Gal---aDHVELPHLRALAQDAVSFRRHYSVT   50

SmCS     PLxCAPARASFMAGQLPSRTRVYDNAAEY-QsSIPTYAHHLRRAGYYTALSGKMHFV-G-  107
SpAS1    TVxCGPSRMSAYTGRYVRSHGSTQNGIPLrV-GEPTLGDHLRDVGMRNVLIGKTHMRpDl  108
SpAS2    PI-CGSSRMSTYTGRYVHSHGASWNGIPLkV-GEMTMGDHLRAAGMGCWLVGKTHMR-A-  102
BcPMH    VPxCGPARASLLTGLYLMNHRAVQNTVPLdQ-RHLNLGKALRAIGYDPALIGYTTTT-P-  110
RlPMH    VPxCGPARASLLTGLYLMNHRAVQNTVPLdQ-RHLNLGKALRGVGYDPALIGYTTTV-P-  110
ArPMH    AP-XSPARATLYTGLYQMNHRVCRNGSPLdA-RFDNLALAARRGGYDPTLFGYTDTA-P-  117
SpPMH    NP-CGPSRASILTGQYAMNHRSVRNGTPLrH-DTPNIATEMRKAGYLPLLFGYTDTS-Q-  106

SmCS     -Pd------------------Q-LhGFeERLTTD---------------Iypadfgwtpd  132
SpAS1    dGmkrlgidpd--seigarvgE-G-GF-DAFDRDdgvhpt---gyrkkePayndylrhag  160
SpAS2    -Deegmarlglepdsligarv A-EcGF-DVFERDdgmlpegpdgyydpdGakeynkflra  159
BcPMH    -Dprttsard----prftvlgDiMdGF-RSVGAF---------------Epnmegyfgwv  149
RlPMH    -Dprttspnd----prfrvlgDlMdGF-HPVGAF---------------Epnmegyfgwv  149
ArPMH    -Dprgmdpnd----phlttyeGvLpGF-SARQLL---------------Pehekqwlswl  156
SpPMH    -Dpraydand----palktyeFpMrGF-HEVTEM---------------Rlemsypwqsh  145

SmCS     yrkpg---eridwwyhnlgsvtG-----A----------------G--VAEItnqM---E  163
SpAS1    fqaenpwefwansaegkggenqSgwlltH----------------AdkPARVp--Ee-hS  201
SpAS2    kg--------yesdnpwhdfanS-----GlddegnvqsgwflknaTr-PANIa--Ee-dS  202
BcPMH    aqn-------gfelpenrediwL-----Pegehs-------vpgaTdkPSRIp--Ke-fS  187
RlPMH    aqn-------gfdlpehrpdiwL-----Pegeda-------vagaTdrPSRIp--Ke-fS  187
ArPMH    rs--------rghpeatsrdihI-----Pvgat--------pgeiSdvAPAY---SkdeT  192
SpPMH    lkn-------rgyafddyaqvyV-----Prpdad------gtprlNg-PAMYr--Ae-dS  183

SmCS     YDDEVAFLANQKLYQLsReNddesrrPWCLTVSFTHPHDPYVARRKFWDLYED-CEHlTP  222
SpAS1    ETAYMTRRAMEFMEAA-E-Kdg---rPWCAHLSYIKPHWPYIVPAPYHDMFGPdDVK-PA  255
SpAS2    ETPYLTSRAMEFIEQQ-T-G------PWCCHLSYIKPHWPYIVPEPYASMFGPeHVQ-DV  253
BcPMH    DSTFFTERALTYLKGR-D-Gk-----PFFLHLGYYRPHPPFVASAPYHAMYKAeDMP-AP  239
RlPMH    DSTFFTERALTYLKGR-D-Gk-----PFFLHLGYYRPHPPFVASAPYHAMYRPeDMP-AP  239
ArPMH    QTAFLAGEFIRWLGEQ-D-A------PWFAHVSFLRPHPPFSVPEPYNRMFTPsDGP-AF  243
SpPMH    DTAFLTDQFLANMPAW-A-Gq-----NWFAHLTYIRPHPPLVAPAPYNTMYDPaKLP-LP  235

SmCS     EVgaIPl--D-Eq----DpHSQRIMLS-C--Dyqn---------fdV--TEENVRRSRRA  261
SpAS1    VR--SDeelKaA-----HpLFKAMTEE-V--Ys------------rNfaRDEVREKVIPA  293
SpAS2    VR--SDserQ-Na----HpLFKAFMDTkVgeAf-------------S--RQEVRDAVIPA  291
BcPMH    IR--AEn-pD-Aea-aqHpLMKHYIDH-I--RrgsffhgaegsgatL--DEGEIRQMRAT  289
RlPMH    IR--AAn-pD-Iea-aqHpLMKFYVDS-I--RrgsffqgaegsgatL--DEAELRQMRAT  289
ArPMH    AR--AAn-rE-Aeq-avHpLLAFALPL-I--GkdsfiyggegsasdW--TSEDLSAIRAI  293
SpPMH    AR--LPg-rD-DetaehP-FFGPATRY-S--Spasfvl--gfpdleP--TDETIQTLRAV  283

SmCS     YFANISYLDEKVGELIDTLTRTRMLDDTLILFCSDHGDMLGERGLWFKMNFFEGSARVPL  321
SpAS1    YMGLIKQIDDQLGQLFAFMQERGLDENTMIVFTADHGDYLGDHWMGEKYLFYEAAAKVPL  353
SpAS2    YMGLIKQADDQMGRLFKWLEDTGRMQDTMIVLTSDHGDFLGDHWMGEKTFFHDASTRVPL  351
BcPMH    YCGLITEIDDCLGRVFAYLDETGQWDDTLIIFTSDHGEQLGDHHLLGKIGYNAESFRIPL  349
RlPMH    YCGLITEVDDCLGRVFSYLDETGQWDDTLIIFTSDHGEQLGDHHLLGKIGYNDPSFRIPL  349
ArPMH    YYGMIAEVDTQLGRIWQALKNVGAWDDTLIIFTSDHAEMMGDHWMLGKGGFFDGSYHVPL  353
SpPMH    YLGLATEVDTHIGRVIAHLKETGQYDDTLIVVTADHGEMLGDRHSWGKMTVYDAAYHTPL  343
```

```
SmCS     MIAGPG---I--AP-GLHLTPTSNLDVTPTLADLAGISleeVR-PWTDGVSLVPMVN-G-   372
SpAS1    IIYDPSdkaDa-TRgTVSDALVEMIDLAPTFVDYAGGV---PPmHILEGKSLLPLLH-Dd   408
SpAS2    IIYDPRpeaDa-TRgSVCDALVESIDLAPTFVEAAGGK---PAmHILEGESLIPILHgAr   407
BcPMH    VIKDAG---QnrHAgQIEEGFSESIDVMPTILEWLGGE---TP-RACDGRSLLPFLA-E-   400
RlPMH    VIKDAG---EnaRAgAIESGFTESIDVMPTILDWLGGK---IP-HACDGLSLLPFLS-E-   400
ArPMH    VIRDPGh--Pg-GAgRQVERFTSAADIFPTLCDRLGLV---PD-NHLDGGTLVPFLEgG-   405
SpPMH    IIRAPG---C--KPgHVVEAPTESIDLMPTILDWVGQE---IP-NAVDGRSLRPFLTgE-   393

SmCS     -V-E---RTEPVLMEYAAEasY-----------------APLVAIReG-KWKYVYCAL--   407
SpAS1    dSsW---DRQYVFSELDYS--Nlparlkl----grdiqdCRATMVF-DgRYKLVEVMG--   456
SpAS2    -D-Ht--LRDHVICEYDFS--Aspiahln----disvrqAVMFMVA-DkNWKLIHFEAdp   456
BcPMH    -G-KpsdWRTELHYEFDFR--Dvfydqpqn-svqlsqddCSLCVIE-DeNYKYVHFAAl-   453
RlPMH    -G-RpqdWRTELHYEYDFR--Dvyysepqs-flglgmndCSLCVIQ-DeRYKYVHFAAl-   453
ArPMH    -E-Pe-gWRDAAFWEFDFR--Diakgeaer-hfglksnaCNLAVIR-DeRFKYVHFAGl-   457
SpPMH    -A-Ps-dWRQYSFSELDIS--Epldptlwqqefgfgpsa GAVAILR-DaRFTLVEFAAdl   447

SmCS     DpEQLFDLEA-Dp-LeLTNLAENPrGpvDQATLTAFRDMRAAHWdmeafdaavresqarr   465
SpAS1    FaPILFDLEVdPd-E-LKDLGRDP-S--AEEVRQRLTSALDAWHrntrqritksdaayra   511
SpAS2    R-PMLFDLKN-DpqE-LVDLGGDP-A--HADVIAGMYDKLFRWTrrqsqrttrseeqlia   510
BcPMH    P-PLFFDLKAdP--HeFSNLAGDP-A--YAALVRDYAQKALSWRlshadrtlthyrsspq   507
RlPMH    P-PLFFDLRHdP--NeFTNLADDP-A--YAALVRDYAQKALSWRlkhadrtlthyrsgpe   507
ArPMH    P-PLLYDLAKdP--MeLTNVAADA-D--YAAVRLGYAEKLLSLRaqhldqtlayteltek   511
SpPMH    P-PMLFDHQGeG--E-FRNVAGDP-A--HAADLARLSRQMLRHRmrnmdhtlslcsithe   500

SmCS     wvvyealrngayypwdhqplqkaserymrnhmnldtleeskryprge   512
SpAS1    ldpvlresdpdlmagviigywdedeveaekrriarilgen-------   551
SpAS2    mrtksrkrgivlgiydenetpleltvkyrdkarpykdylkg------   552
BcPMH    glttrnh----------------------------------------   514
RlPMH    glsersh----------------------------------------   514
ArPMH    gpvsrrp----------------------------------------   518
SpPMH    gartqrryd--------------------------------------   509
```

Fig. S4. Multiple sequence alignment data of the structural alignment of several AP-superfamily sulfatases: choline sulfatase (CS), dimeric arylsulfatases (ASs)) [9] and phosphonate monoester hydrolases (PMHs) [9-11].

See Table S7 for PDB IDs of the structures included. The conserved active site residues (see Table S8) are indicated in blue, both oligomerization interfaces are indicated in the color coding corresponding to Fig. S3a-c. Residues that do not align structurally are in lower case letters

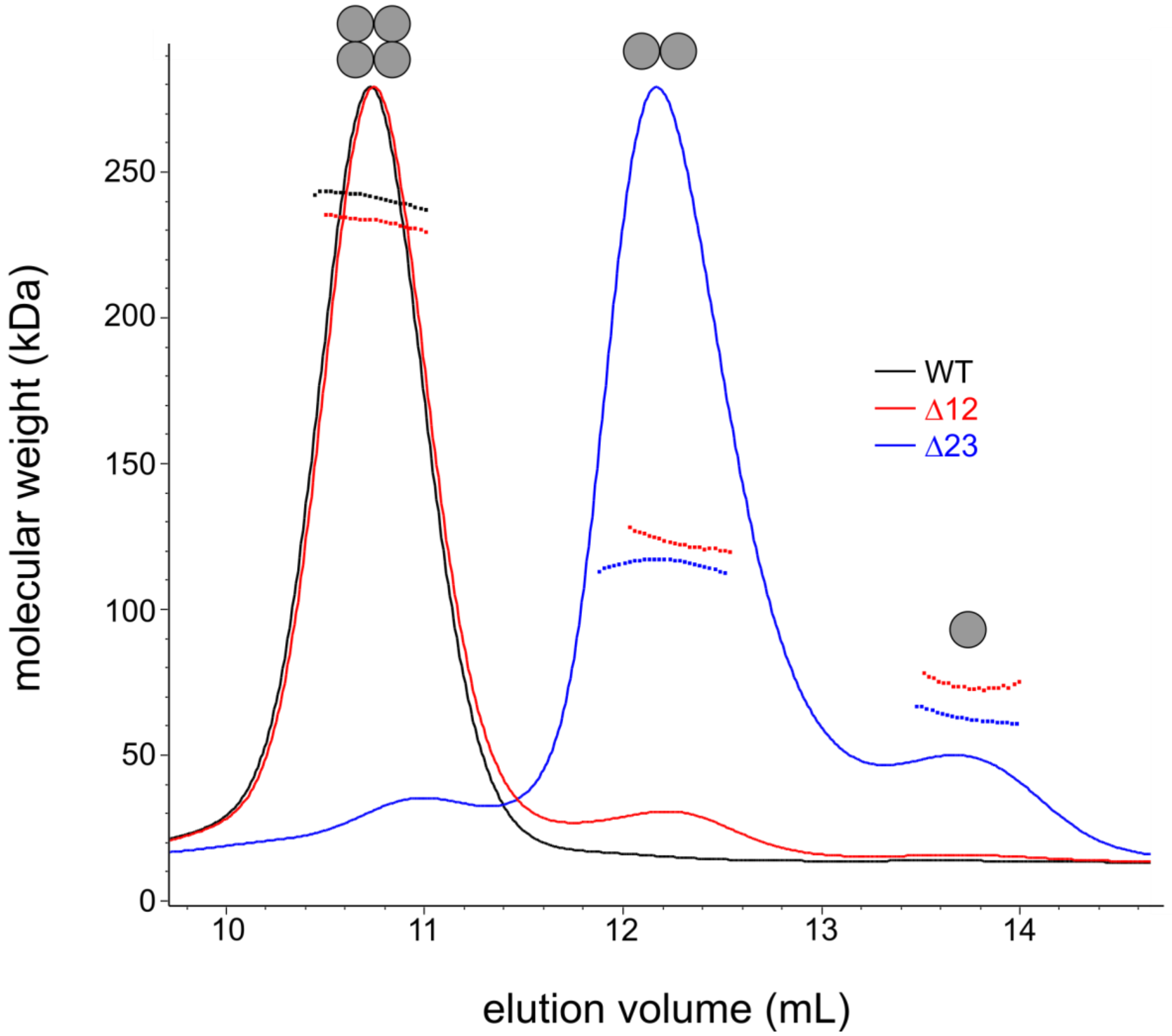

Protein	Tetramer peak MW / mass fraction	Dimer peak MW / mass fraction	Monomer peak MW / mass fraction
*Sm*CS WT	241.3 kDa / 100%	n/d / 0%	n/d/ 0%
*Sm*CS Δ12	233.2 KDa / 86%	123.2 kDa / 11 %	74.3 kDa / 3%
*Sm*CS Δ23	n/d / 0%	119.6 kDa / 81%	63.2 kDa / 19 %

Fig. S5: Elution patterns for *Sm*CS WT (black), Δ12 (red) and Δ23 (blue) on a Superdex 200 size-exclusion column.

Accurate molecular weight data for each variant were obtained by multi-angle light scattering (MALS) and differential refractive index detection directly coupled to the size exclusion chromatography column. These data stand in contradiction to the observations of Sanchez-Romero & Olguin [3] (see discussion above). n/d, not detected.

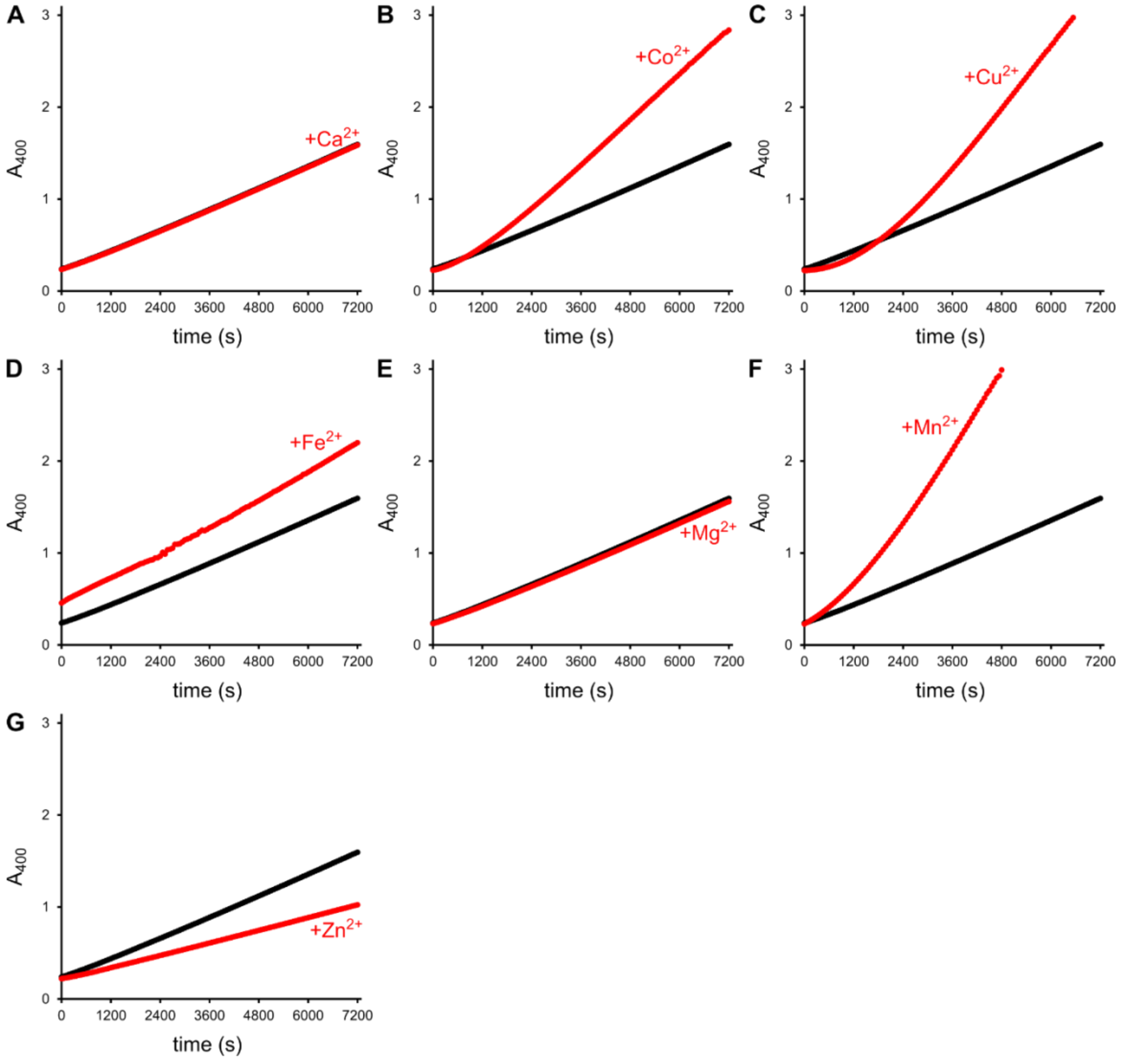

Fig. S6: Stimulation of *Sm*CS WT-catalyzed hydrolysis of 4-nitrophenyl sulfate (sulfate monoester **1b**) by various divalent metal ions.

The experiments were performed in 100 mM Tris-HCl pH 7.6; [M^{2+}] = 0.5 mM; [*Sm*CS WT] = 1.43 μM; [4-nitrophenyl sulfate] = 10.5 mM at 25 °C. Of the metals observed during the microPIXE measurements (Ca^{2+}, Zn^{2+} and Fe^{2+} and Mn^{2+}; Table S8), only Mn^{2+} significantly stimulated enzyme activity, whereas addition of an excess Ca^{2+}, the most abundant metal in the microPIXE experiment, had no effect on activity.

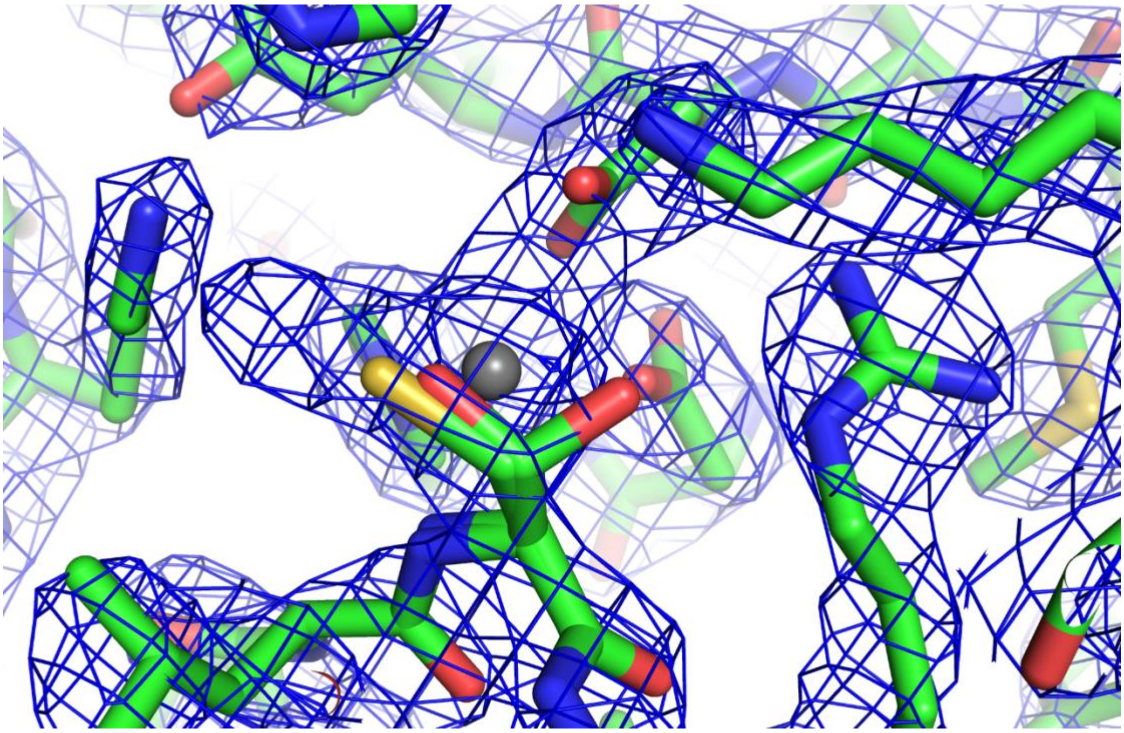

Fig. S7: Electron density map for the active site nucleophile in *Sm*CS.

Weighted $2F_oF_c$ electron density map of the refined structure is shown at 1σ level around the nucleophile in the active site with the nucleophile modelled as a mixture of cysteine and formyl glycine and Ca^{2+} as the active site metal ion.

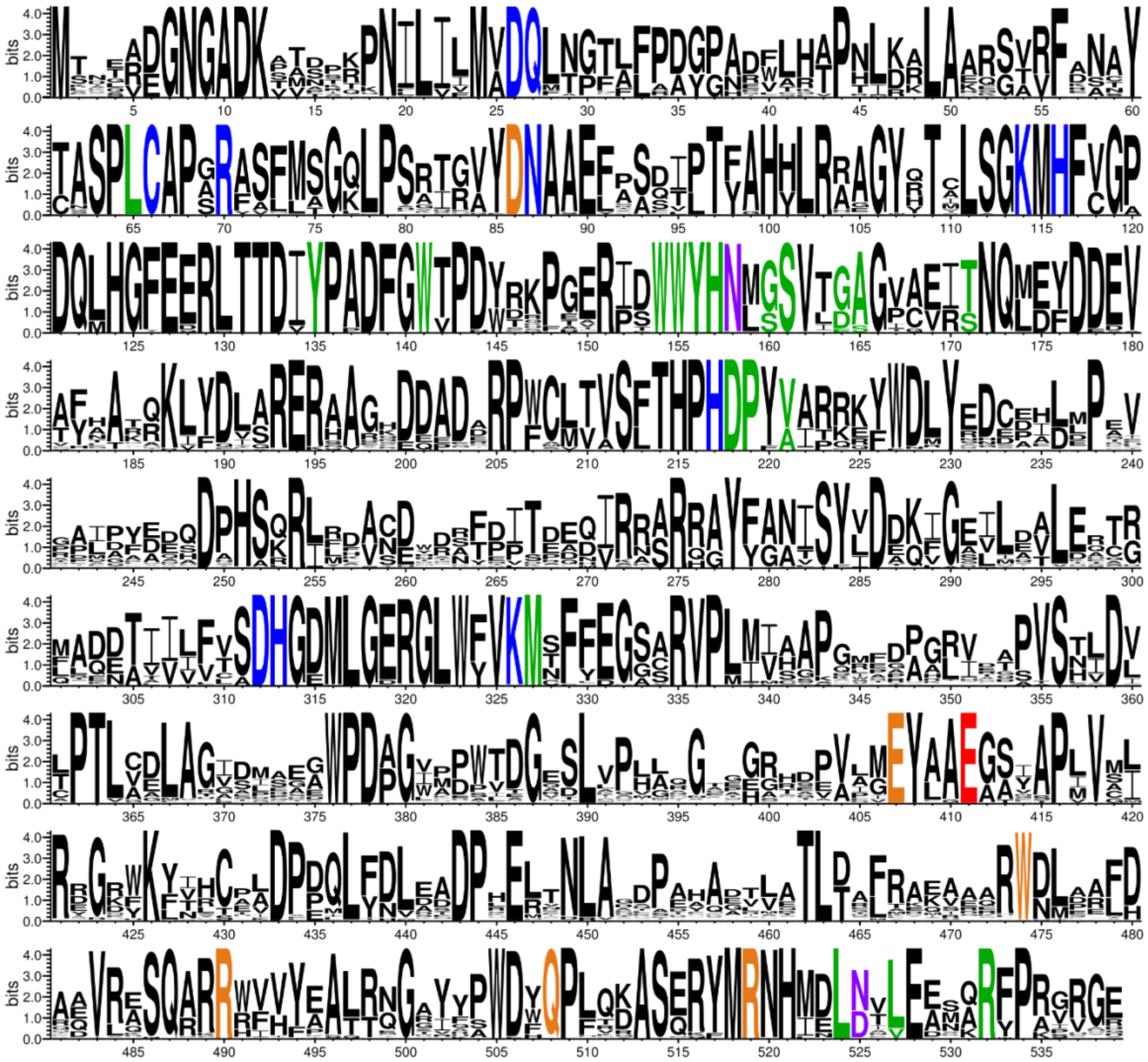

Fig. S8. Logo representation of the multiple sequence alignment of 87 different choline sulfatases.

The height of the letters reflects the conservation of that particular residue (see Table S14 for details on the included sequences). Active-site positions that are conserved between CSs, ASs and PMHs are indicated in blue. The position corresponding to the glutamate residue involved in quaternary ammonium binding (Glu386 in *Sm*CS) is indicated in red. Residues that form salt-bridges as part of the oligomerization interface(s) are indicated in orange (see also Fig 4c). The hydrogen bonding residue pair that 'controls' the entrance to the active site is indicated in purple (Asn146 and Asp500 in *Sm*CS, see also Fig. 5b). Residues indicated in green are part of the active site cavity (see also Fig 5c and Table S9).

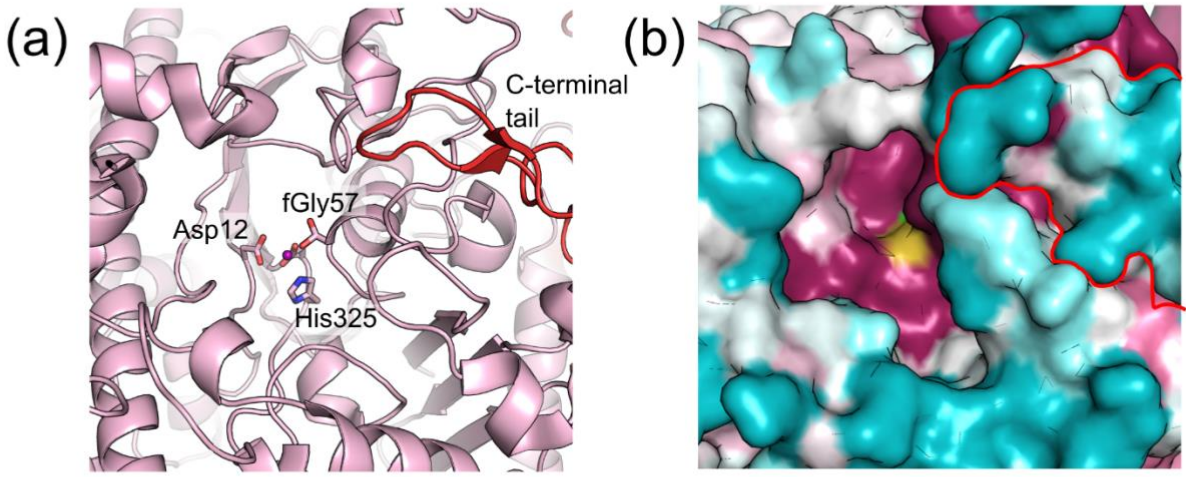

Fig. S9. Active site geometry and conservation of *Rl*PMH.

In contrast to the narrow, L-shaped active site of *Sm*CS, *Rl*PMH has a wide-open, solvent-exposed active site 'valley' which, apart from the catalytic site, is relatively poorly conserved among PMHs. (**a**) Ribbon diagram of the wide-open active site of *Rl*PMH with one protomer colored in pink and the other one, with only its C-terminal tail visible, in red. (**b**) The same view as in (**a**) but showing the molecular surface of the protein, colored by conservation with purple being most conserved and dark cyan non-conserved. The active site is marked with yellow and the tail of the adjoining protomer is outlined with red.

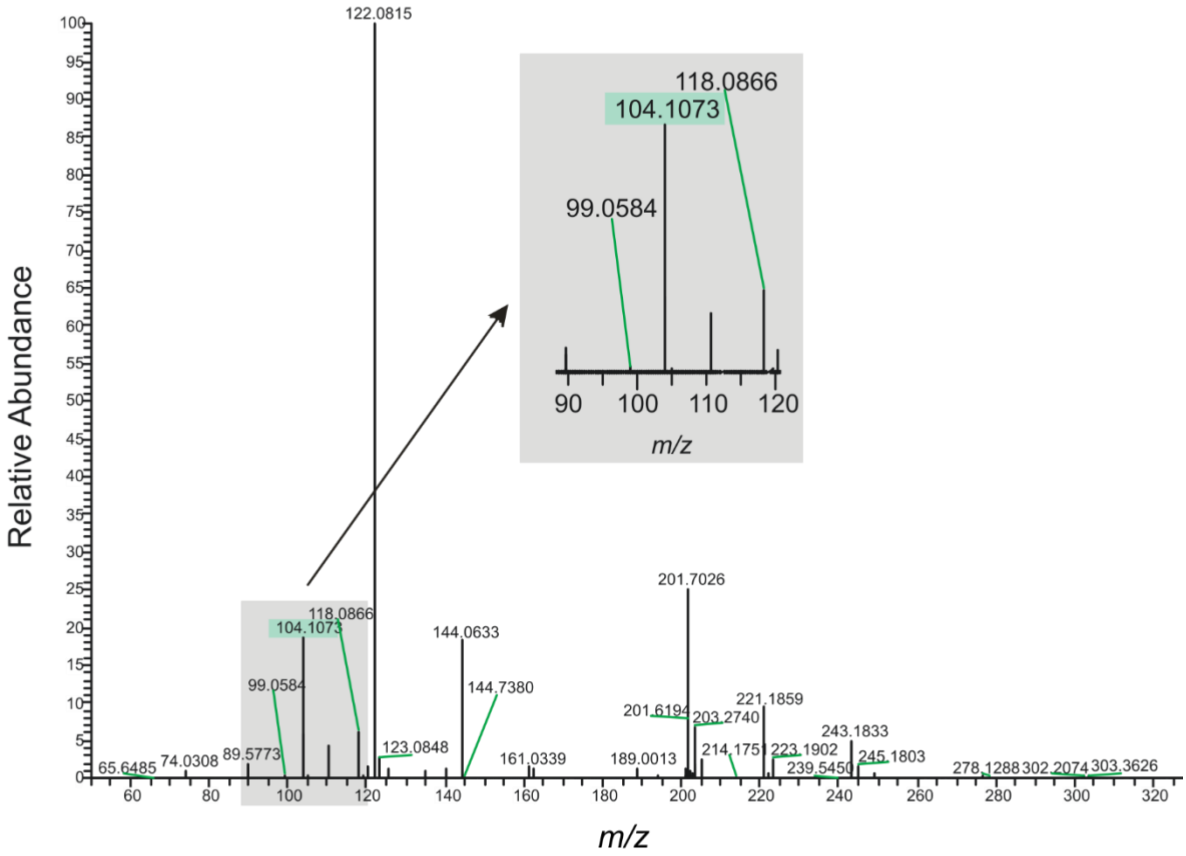

Fig. S10. ESI-MS spectrum for choline (*m/z* 104.1073, highlighted) resulting from the *Sm*CS-catalyzed hydrolysis of 5 mM choline-*O*-sulfate (**1a**) in the presence of 50% (v/v) $H_2{}^{18}O$ in 20 mM Tris-HCl pH 7.6.

The large peak at *m/z* 122.0815 is from Tris-H^+. The absence of a peak at *m/z* 106.1 indicates there is no ^{18}O-incorporation in the choline leaving group as a result of enzyme-catalyzed hydrolysis. These data suggest that *Sm*CS catalyzes hydrolysis of sulfate monoester **1a** by attack at the sulfur center.

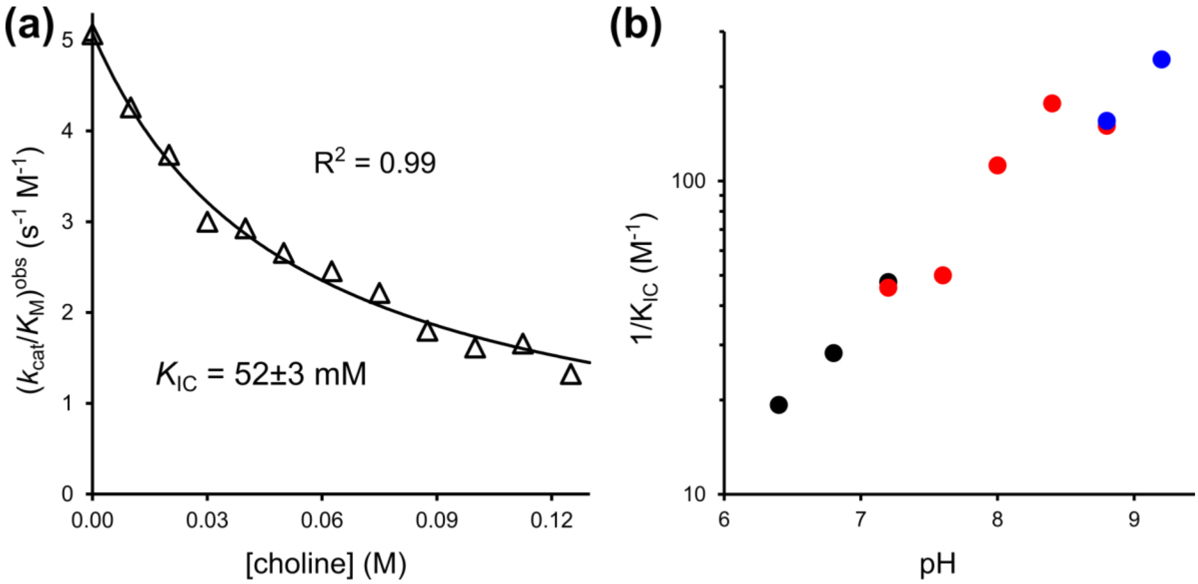

Fig. S11. Inhibition of *Sm*CS-catalyzed hydrolysis of 4-nitrophenyl sulfate (**1b**) by choline. (**a**) Observed k_{cat}/K_M-values determined in the presence of increasing concentrations of choline, recorded in 100 mM imidazole-HCl buffer at pH 6.4. The data were fitted to the equation $(k_{cat}/K_M)^{obs} = (k_{cat}/K_M)^{\text{no inhibitor}} \times (1/(1+[\text{choline}]/K_{IC})$. (**b**) *Increased binding affinity (1/K_I) with increasing pH.* Data were determined in 100 mM imidazole-HCl (black circles), Tris-HCl (red) and glycine-NaOH (blue).

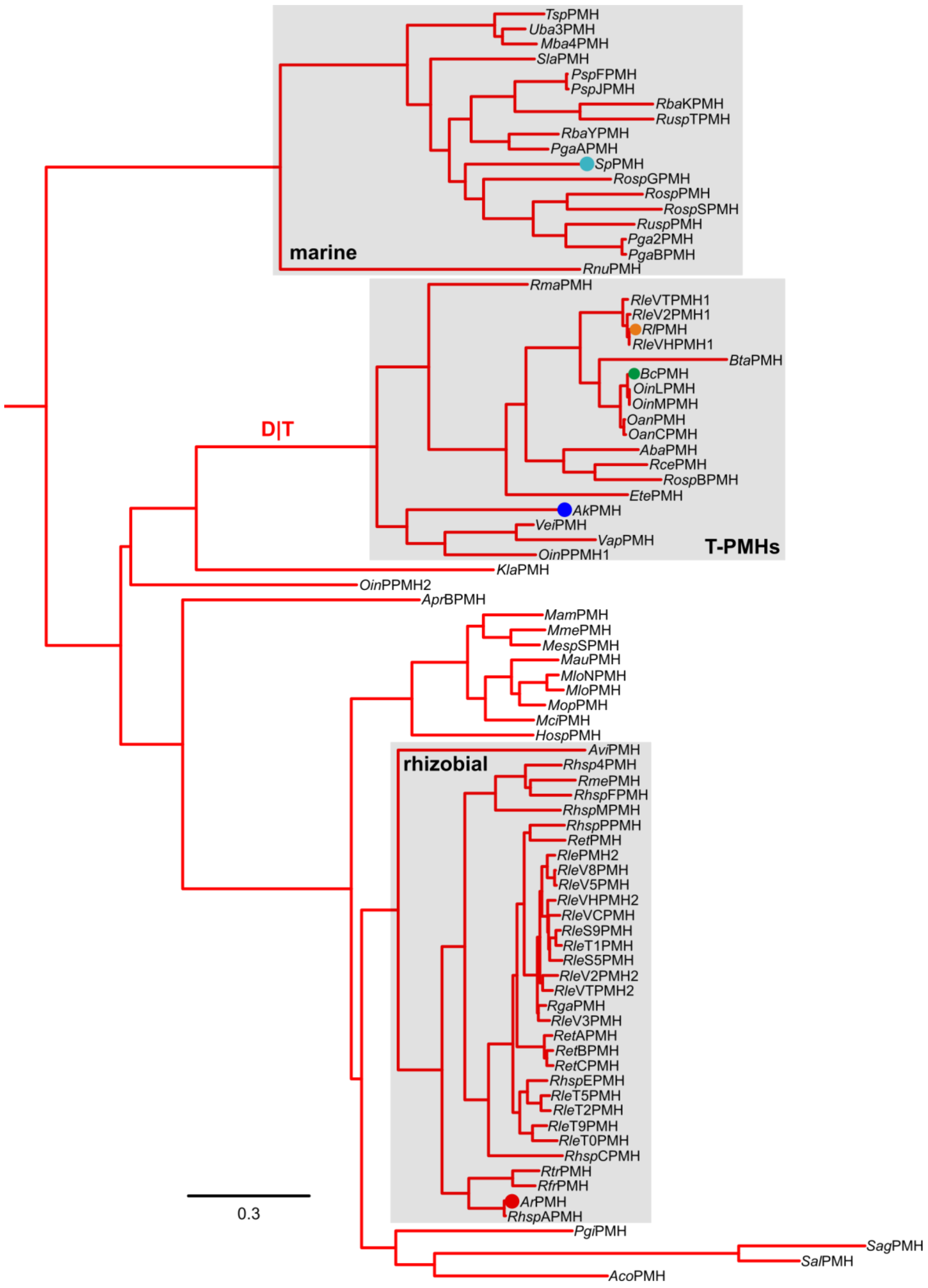

Fig. S12. PMH clade from Fig. 8 displayed in full detail and with labelled taxa.

A description of the enzyme names is reported in Table S12.

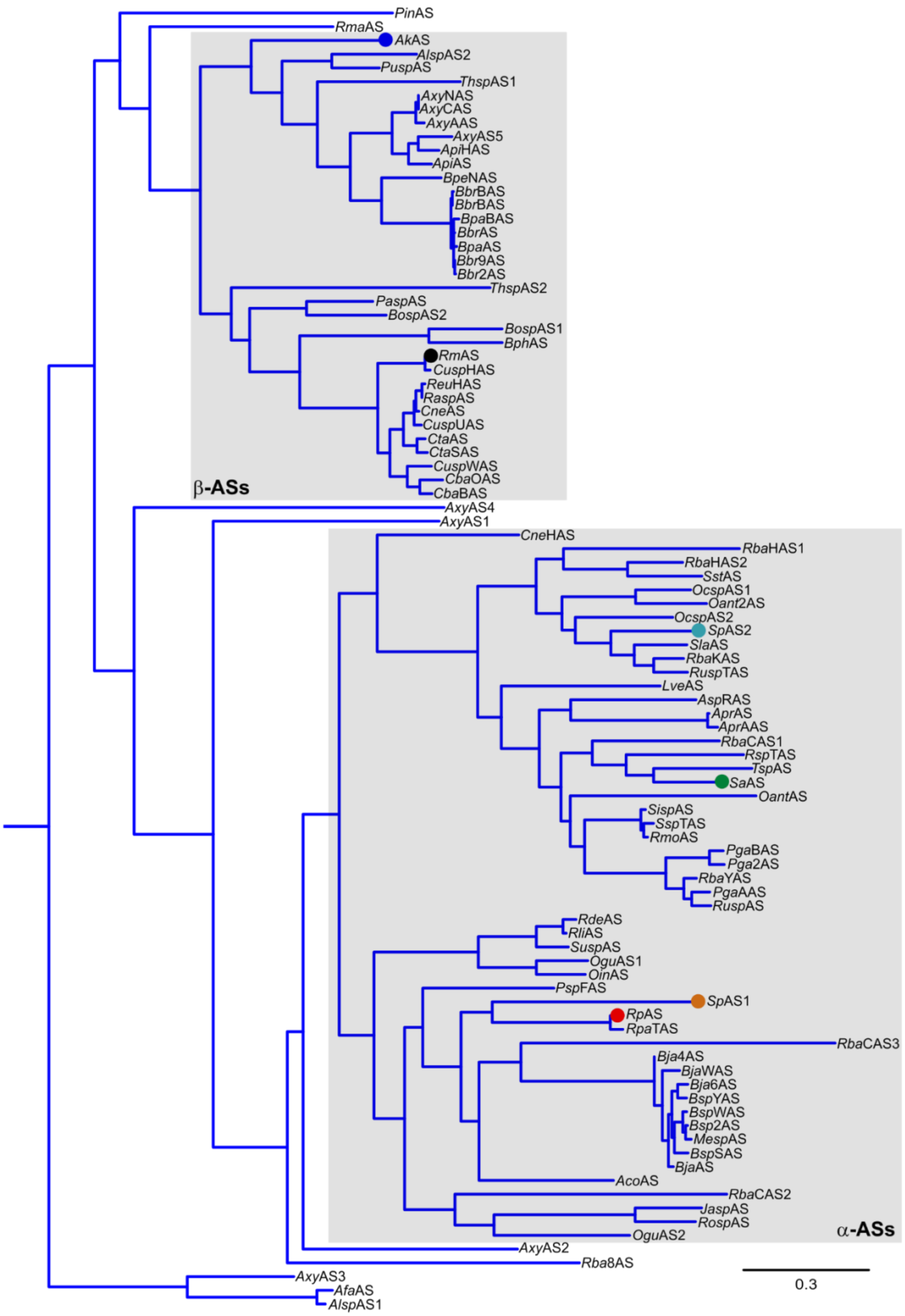

Fig. S13. AS clade from Fig. 8 displayed in full detail and with labelled taxa.

A description of the enzyme names is reported in Table S13.

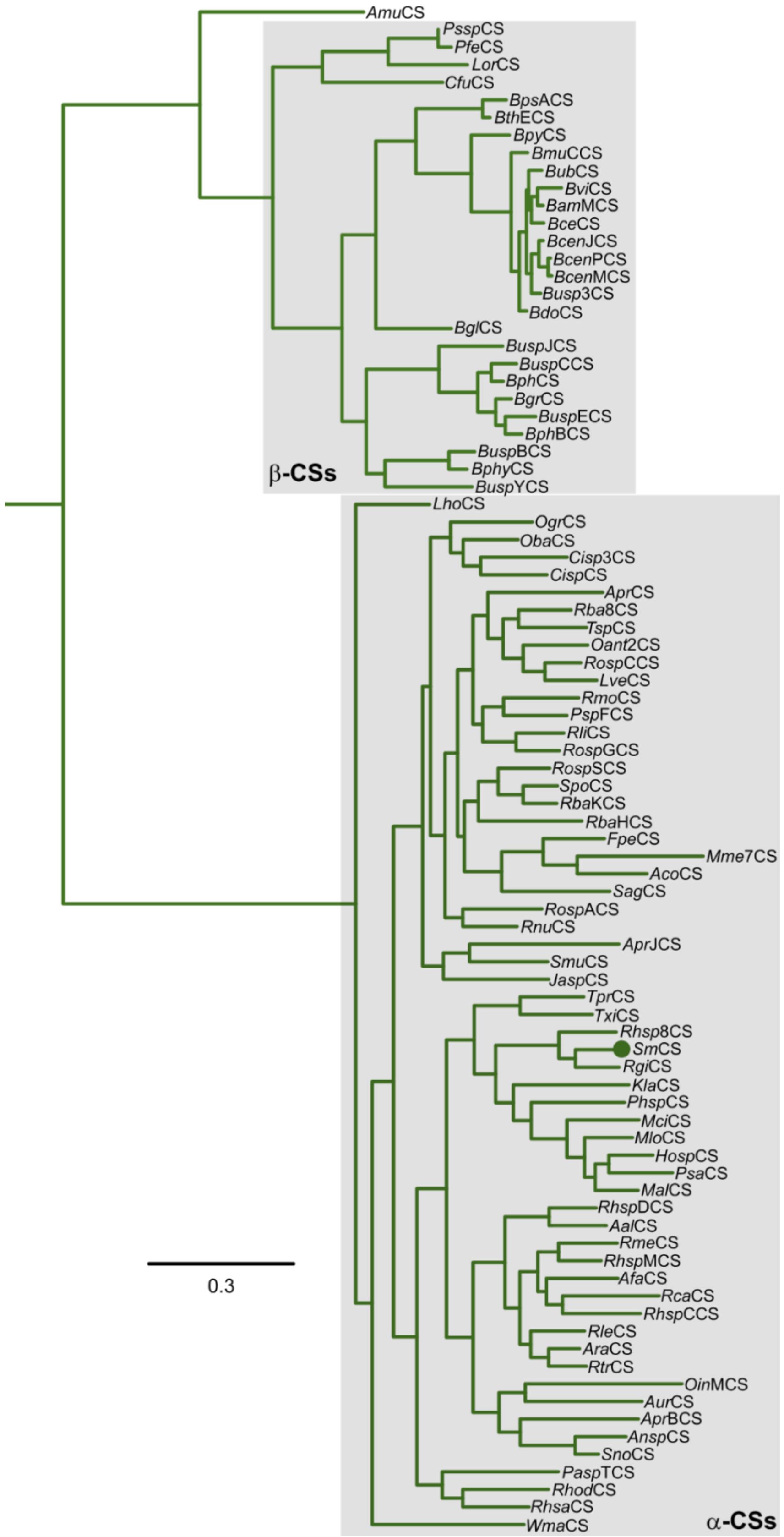

Fig. S14. CS clade from Fig. 8 displayed in full detail and with labelled taxa.
A description of the enzyme names is reported in Table S14.

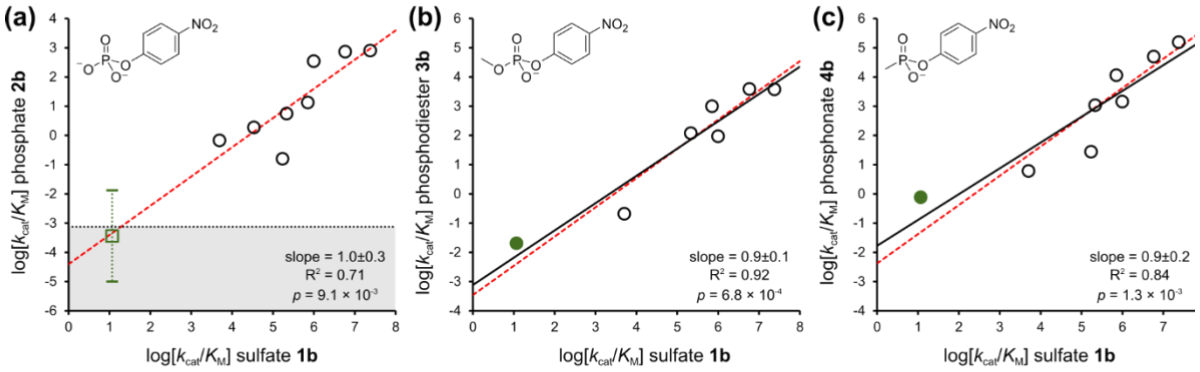

Fig. S15. Correlation of primary and promiscuous activities for sulfatases.

The previously published correlations between the catalytic efficiencies (k_{cat}/K_M values) toward sulfate monoester **1b** and phosphoesters **2b**-**4b** (panel (**a**)-(**c**)) for sulfatases [9] (black open circles) were extended with the data for *Sm*CS (blue circle in panel (**b**) and (**c**). The black solid line represents the fitted linear correlation with the slope, R^2 and *p*-value indicated at the bottom right of the graph. The red dotted line represents the fitted linear correlation when the slope is fixed at 1. The inclusion of *Sm*CS does change the correlation slope for phosphodiester **3b** and phosphonate monoester **4b**, it is however still close to 1. Extrapolation of the correlation for phosphate monoester **2b** to the k_{cat}/K_M-value for sulfate monoester **1b** shows an expected value below the indicated detection limit of 7.5 × 10^{-4} s^{-1} M^{-1} (blue square, with calculated confidence indicated. Despite its relatively low ‘primary’ activity toward sulfate monoester **1b**, *Sm*CS behaves like other AP-superfamily-type arylsulfatases towards sulfo- and phosphoesters **1b**-**4b**.

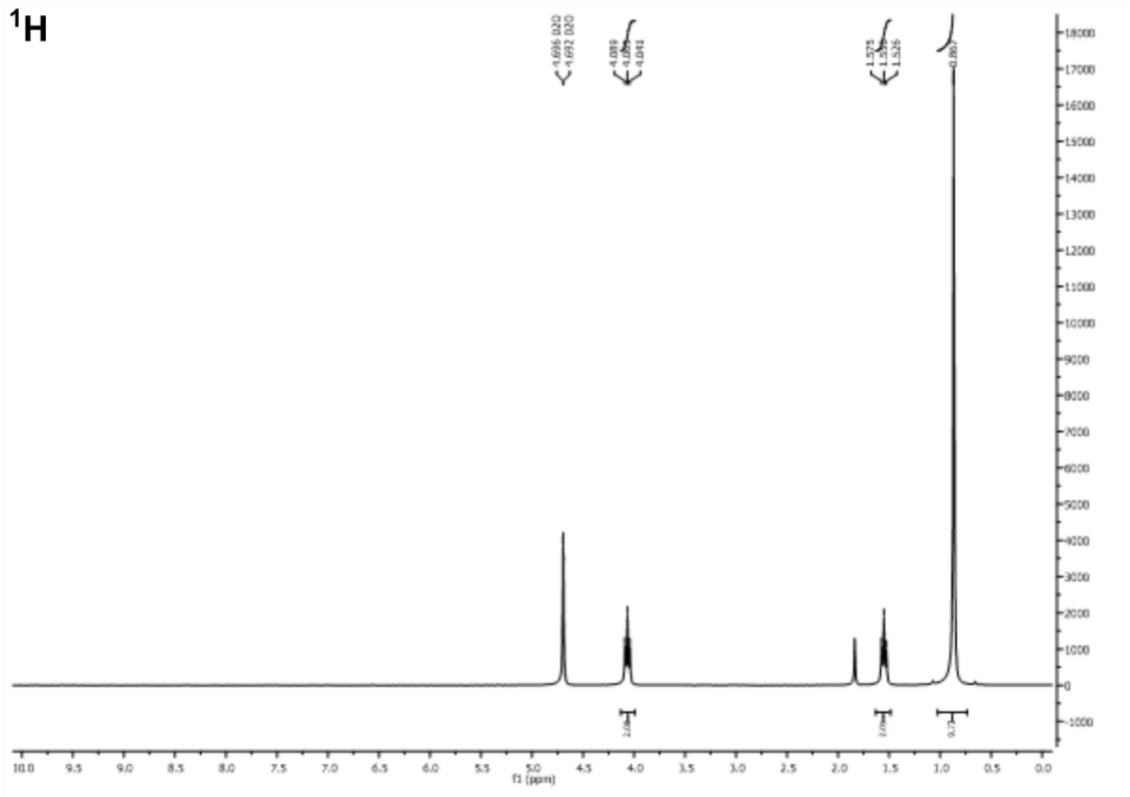

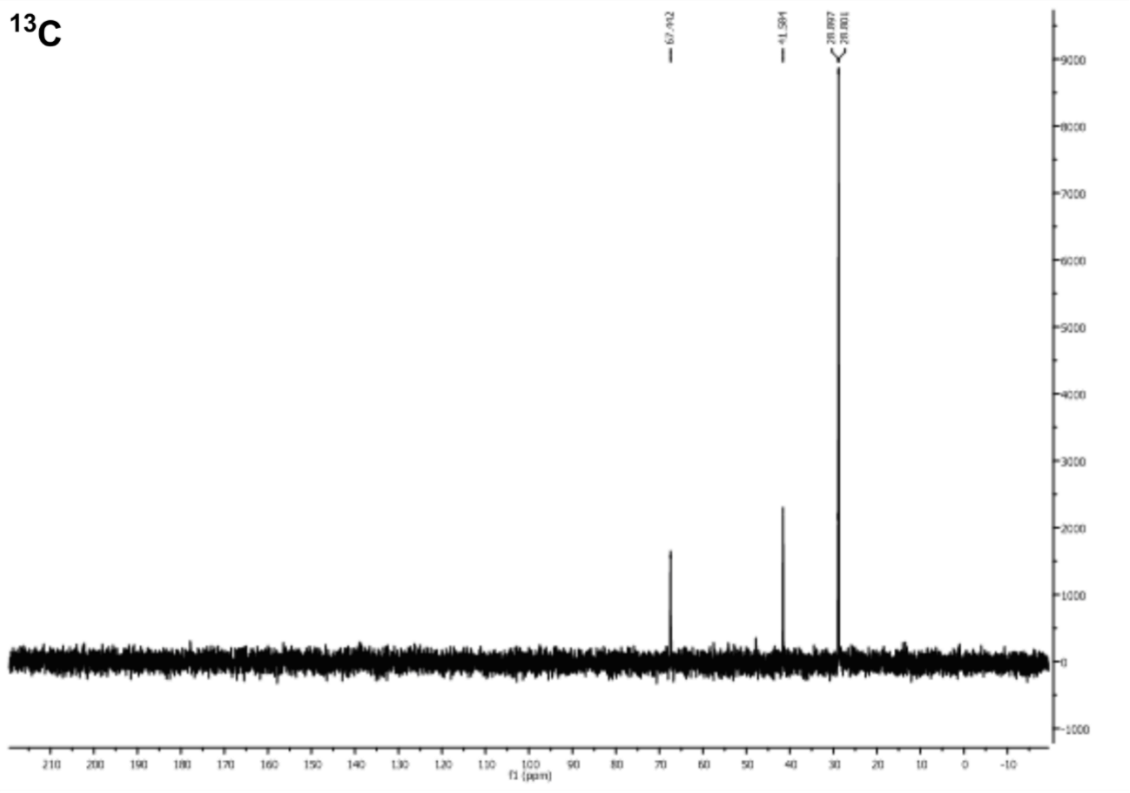

Fig S16. ^{1}H-NMR and ^{13}C-NMR spectra for 3,3-dimethylbutyl sulfate (**1c**).

^{1}H NMR (300 MHz, D_2O): δ = 4.07 (t, J = 7.2 Hz, 2H), 1.55 (t, J = 7.4 Hz, 2H), 0.87 (s, 10H);

^{13}C NMR (75 MHz, D_2O): δ = 67.4, 41.6, 28.9, 28.8.

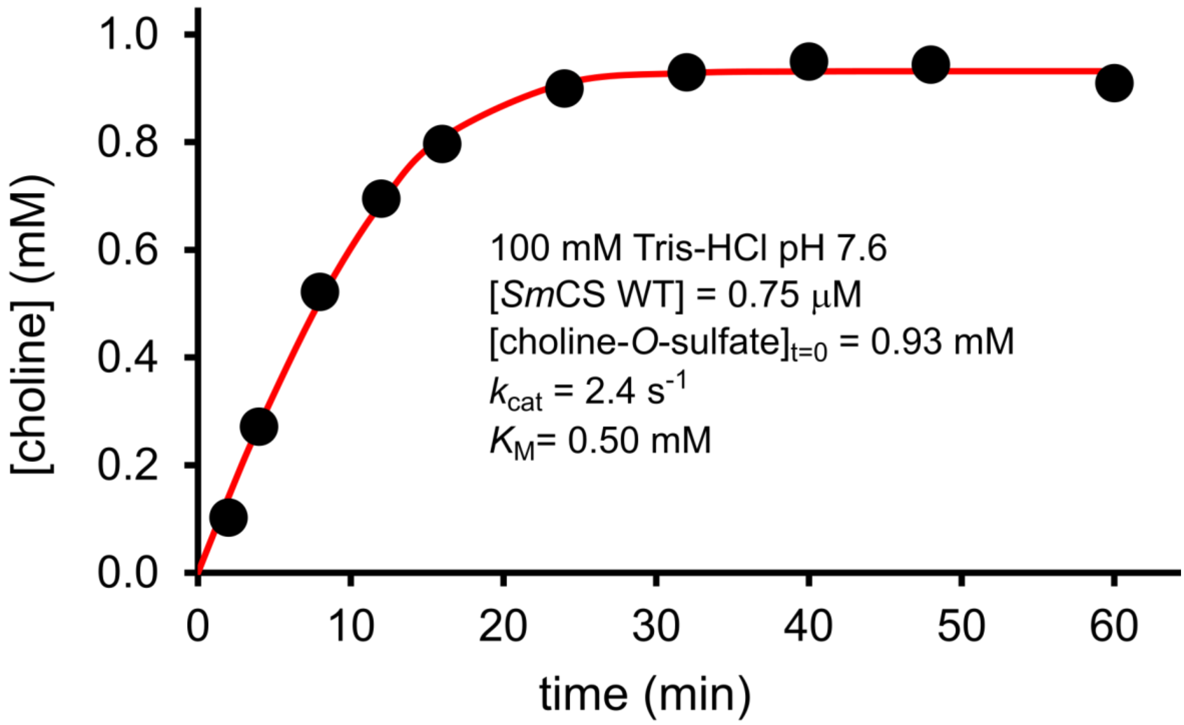

Fig S17. Example fit for determining choline sulfatase activity.

Black dots show the amount of choline formed at specific time points. The red curve is the numerical fit calculated with Micromath Scientist according to equations (3 and 5) described in the materials and methods.

Supporting Tables

Table S1. Kinetic data for the pH rate profile of *Sm*CS wild type for 4-nitrophenyl sulfate **1b** and choline-*O*-sulfate **1a**.

Substrate	pH	Buffer (100 mM)	k_{cat}/K_M (s^{-1} M^{-1})
**1b**	6.0	Imidazole-HCl	$(1.2\pm0.2) \times 10^1$
	6.4		$(1.0\pm0.2) \times 10^1$
	6.8		5.8 ± 0.7
	7.2		4.2 ± 0.6
	7.2	Tris-HCl	3.9 ± 0.7
	7.6		2.5 ± 0.3
	8.0		1.7 ± 0.6
	8.4		$(7.6\pm0.1) \times 10^{-1}$
	8.8		$(4.5\pm0.0) \times 10^{-1}$
	8.8	Glycine-NaOH	$(7.9\pm0.1) \times 10^{-1}$
	9.2		$(3.3\pm0.0) \times 10^{-1}$
	9.6		$(2.4\pm0.0) \times 10^{-1}$
	10.0		$(9.2\pm0.3) \times 10^{-2}$
**1a**	6.8	Imidazole-HCl	$(1.3\pm0.2) \times 10^3$
	7.2		$(2.1\pm0.2) \times 10^3$
	7.2	Tris-HCl	$(3.6\pm0.3) \times 10^3$
	7.6		$(4.8\pm0.5) \times 10^3$
	8.0		$(4.8\pm0.2) \times 10^3$
	8.4		$(2.7\pm0.1) \times 10^3$
	8.8		$(2.1\pm0.0) \times 10^3$

Table S2. First (k_1) and second (k_2) order rate constants for uncatalyzed hydrolysis of sulfoesters an phosphoesters used in this study (Temperature = 25 ^oC).

substrate	k_1^a (s^{-1})	k_2^a (s^{-1} M^{-1})
**1a**[b]	2.8×10^{-22}	5.0×10^{-24}
**1b**[c]	1.9×10^{-10}	3.5×10^{-12}
**2a** (mononanion)[d]	2.2×10^{-9}	3.9×10^{-11}
**2a** (dianion)[e]	4.8×10^{-18}	8.7×10^{-20}
**2b** (mononanion)[f]	1.6×10^{-7}	2.8×10^{-9}
**2b** (dianion)[g]	1.3×10^{-9}	2.4×10^{-11}
**3b**[h]	3.7×10^{-10}	6.8×10^{-12}
**4b**[i]	1.2×10^{-9}	2.1×10^{-11}

[a]Based on either published k_1 (a.k.a. k_{uncat}) or k_2 (a.k.a. k_w). k_1 and k_2 are correlated via the formula $k_1 = k_2 \times [H_2O]$, in which $[H_2O]$ in a normal aqueous solution is 55 M.

[b]Rate constants for nucleophilic attack at the sulfur center (S-O attack) extrapolated from the published leaving group dependence [1], assuming a pK_a of 13.9 [2] for the choline leaving group.

[c]According to Edwards *et al*. [1].

[d]Derived from the rate constant for the hydrolysis of the monoanionic form of phosphate monoester **2b** ($k_{monoanion}$) using the published Brønsted relation for the hydrolysis of phosphate monoester monoanions ($\beta_{leaving\ group}$ = -0.27 [12]) assuming pK_a values of 7.02 [13] and 13.9 [2] for 4-nitrophenol and choline, respectively. The $k_{monoanion}$ for phosphate monoester **2b** was calculated from the published $\Delta H^{\ddagger}$ and $\Delta S^{\ddagger}$ values [12].

[e]Derived from the rate constant for the hydrolysis of the dianionic form of phosphate monoester **2b** ($k_{dianion}$) using the published Brønsted relation for the hydrolysis of phosphate monoester dianions ($\beta_{leaving\ group}$ = -1.23 [14]) assuming pK_a values of 7.02 [13] and 13.9 [2] for 4-nitrophenol and choline, respectively. The $k_{dianion}$ for phosphate monoester **2b** was calculated from the published $\Delta H^{\ddagger}$ and $\Delta S^{\ddagger}$ values [14].

[f]Calculated from the published $\Delta H^{\ddagger}$ and $\Delta S^{\ddagger}$ values for $k_{monoanion}$ [12].

[g]Calculated from the published $\Delta H^{\ddagger}$ and $\Delta S^{\ddagger}$ values for $k_{dianion}$ [14].

[h]Derived from the second order rate constant for hydroxide attack (k_{OH}) at 25 ^oC, using a β_{nuc} of +0.31 [15] and a pK_{nuc} of -1.7 and 15.7 for H_2O and OH^- respectively. The k_{OH} at 25 ^oC was estimated from the published k_{OH} at 42 ^oC [16], using the reported temperature dependence for the k_{OH} of 4-nitrophenyl ethylphosphate [17].

[i]Derived from the second order rate constant for hydroxide attack (k_{OH}) at 25 ^oC [18], using a β_{nuc} of +0.33 [18] and a pK_{nuc} of -1.7 and 15.7 for H_2O and OH^-, respectively.

Table S3. Comparison kinetic data from Sanchez-Romero & Olguin (2015) [3] with the data from our study.

substrate	kinetic parameters	Data from [3][a]	Our data[b]	ratio
**1a**	k_{cat} (s^{-1})	0.27±0.02	2.4±0.2	8.9±1.0
	K_M (M)	$(1.1±0.1) \times 10^{-2}$	$(5.0±0.6) \times 10^{-4}$	$(4.5±0.7) \times 10^{-2}$
	k_{cat}/K_M (s^{-1} M^{-1})	$(2.4±0.3) \times 10^{1}$	$(4.8±0.5) \times 10^{3}$	$(2.1±0.3) \times 10^{2}$
**1b**	k_{cat} (s^{-1})	$(3.5±0.4) \times 10^{-2}$	$(3.4±0.7) \times 10^{-1}$	9.7±2.3
	K_M (M)	$(7.6±1.5) \times 10^{-2}$	$(9.9±1.9) \times 10^{-2}$	1.3±0.4
	k_{cat}/K_M (s^{-1} M^{-1})	$(4.6±1.1) \times 10^{-1}$	3.4±1.0	7.4±2.8

[a]In 200 mM Tris-HCl pH 7.5 + 500 mM NaCl at 25 °C.

[b]In 100 mM Tris-HCl pH 7.6 at 25 °C.

Table S4. Crystallographic data collection and refinement statistics. The coordinates and the structure factors have been submitted to Protein Data Bank (PDB) with accession code 6FNY.

**Data collection statistics**	
Wavelength (Å)	0.87260
Synchrotron/beamline	ESRF/id23-2
Data collection temperature (K)	100
Space group	$P2_1$
Unit cell parameters: a, b, c (Å); α, β, γ (°)	119.6, 163.2, 177.6; 90, 94.4, 90
Resolution range (outer shell in brackets; Å)	95.55 – 2.79 (2.84 – 2.79)
Unique reflections	167,730
Total observations	649,768
$<I / \sigma(I)>$: all (outer shell)	14.7 (1.4)
$R_{pim.}$: all (outer shell)	11.6 (59.0)
$CC_{1/2}$	0.958 (0.992)
Completeness: all (outer shell) (%)	99.4 (89.0)
Multiplicity	3.9
Wilson B-factor (Å^2)	43.5

**Refinement statistics**	
Non-hydrogen protein atoms (solvent)	34,715 (1713)
Metal ions	8 × Ca^{2+}
Solvent content (%)	66.5
Bond length deviation from ideal values (Å)	0.003
Bond angle deviation from ideal values (°)	0.57
Peptide omega torsion angles (°)	2.56
Average B-factor protein/solvent (Å^2)	27.6 /22.9
Ramachandran favoured/allowed/outliers (%)	95.76/4.1/0.17
All-atom clashscore (100th percentile)	6.27
Random reflections assigned for cross-validation	8,431
R_{work}/R_{free} (%)	20.5/25.2
MolProbity score (99th percentile)	1.89
Luzzati plot estimate of the coordinate error (Å)	0.38

Table S5. PDBePISA oligomerization analysis [19] (http://www.ebi.ac.uk/pdbe/pisa/) of *Sm*CS.

	Interface 1 (red[a])	Interface 2 (green[a])
Area (Å)	2761 (13.2%)	1604 (7.7%)
P-value	0.592	0.224
ΔG_f (kcal mol^{-1})	-9.5	-12.1

[a]As indicated in Fig. S3a and Fig. S4.

Table S6. All AP-superfamily type sulfatases and phosphonate monoester hydrolases of known structure.

Name	Origin	PDB ID	Reference
*Sm*CS	*Sinorhizobium meliloti*	6FNY	This study
*Ar*PMH	*Agrobacterium radiobacter* K84	4UPH	[9]
*Bc*PMH	*Burkholderia caryophili* PG2982	2W8S	[11]
*Rl*PMH	*Rhizobium leguminosarum bv. viciae* 3841	2VQR	[10]
*Sp*PMH	*Silicibacter pomeroyi* DSS-3	4UPK	[9]
*Sp*AS1	*Silicibacter pomeroyi* DSS-3	4UPI	[9]
*Sp*AS2	*Silicibacter pomeroyi* DSS-3	4UPL	[9]
*Bf*AS	*Bacteroides fragilis*	2QZU	
*Bt*2OS	*Bacteroides thetaiotaomicon* Vpi-5482	3B5Q	[20]
*Bt*6OS2	*Bacteroides thetaiotaomicon* Vpi-5482	5G2V	
*Ec*AS	*Escherichia coli*	3ED4	
PAS	*Pseudomonas aeruginosa*	1HDH	[21]
*Hs*ASA	*Homo sapiens*	1AUK	[22]
*Hs*ASB	*Homo sapiens*	1FSU	[23]
*Hs*ASC	*Homo sapiens*	1P49	[24]
*Hs*GalN6S	*Homo sapiens*	4FDI	[25]
*Hs*I2S	*Homo sapiens*	5FQL	[26]
*Hs*SGSH	*Homo sapiens*	4MHX	[27]

Table S7. Putative active site residues[a] for *Sm*CS, arylsulfatases (ASs) and phosphonate monoester hydrolases (PMHs) of known structure.

	fGly	Asn^A	Asn^B	Asp^A	Asp^B	Asp^C	Arg^A	His^A	His^B	Lys^A	Lys^B
*Sm*CS	Cys54	His297	Asn75	Asp296	Gln15	Asp14	Arg58	H104	His201	Lys102	Lys309
*Ar*PMH	Cys64	His329	Asn85	Asp328	Gln25	Asp24	Arg68	Asp114	His222	Tyr112	Lys341
*Bc*PMH	Cys57	His325	Asn78	Asp324	Gln13	Asp12	Arg61	Thr107	His218	Tyr105	Lys337
*Rl*PMH	Cys57	His325	Asn78	Asp324	Gln13	Asp12	Arg61	Thr107	His218	Tyr105	Lys337
*Sp*PMH	Cys53	His319	Asn74	Asp318	Gln14	Asp13	Arg57	Asp103	His214	Tyr101	Lys331
*Sp*AS1	Cys53	His329	Asn74	Asp328	Gln14	Asp13	Arg57	His103	His234	Lys101	Lys341
*Sp*AS2	Cys49	His327	Asn70	Asp326	Gln10	Asp9	Arg53	His99	His232	Lys97	Lys339
*Bf*AS	Ser76	His314	Asn97	Asp313	Gln37	Asp36	Arg80	His134	His232	Lys132	Lys326
*Bt*2OS	Ser64	His284	Asn85	Asp283	His25	Asp24	Arg68	His119	His180	Lys117	Lys296
*Bt*6OS2	Ser77	Gln350	Asn98	Asp37	Asp38	Asp349	Arg81	His127	His199	Lys125	Lys362
*Ec*AS	Ser91	Asn320	Trp112	Asp319	Asp52	Asp51	Arg95	His146	His242	Lys144	Lys346
PAS	Cys51	Asn318	Met72	Asp317	Asp14	Asp13	Arg55	His115	His211	Lys113	Lys375
*Hs*ASA	Cys69	Asn282	Val91	Asp281	Asp30	Asp29	Arg73	His125	His229	Lys123	Lys302
*Hs*ASB	Cys91	Asn301	Ile114	Asp300	Asp54	Asp53	Arg95	His147	His242	Lys145	Lys318
*Hs*ASC	Cys75	Gln343	Val101	Asp342	Asp36	Asp35	Arg79	His136	His290	Lys134	Lys368
*Hs*GalN6S	Cys79	Asn289	Tyr108	Asp288	Asp39	Asp40	Arg83	His142	His236	Lys140	Lys310
*Hs*I2S	Cys84	His335	Phe105	Asp334	Asp45	Asp46	Arg88	His137	His229	Lys135	Lys347
*Hs*SGSH	Cys70	Asn274	Leu91	Asp273	Asp31	Asp32	Arg74	His125	His181	Lys123	Arg282

[a] Nomenclature according to Hanson *et al*. [28], expanded with Asn^B [10], as done previously [9].

Table S8. Metal occupancy according to MicroPIXE measurements

Metal	*Sm*CS-Occupancy	2YT metal content[b]
Ca	0.39±0.09	0.296 (240)
Cu	-	0.014 (11.2)
Fe	0.11±0.05	0.032 (26)
Mg[a]	-	0.644 (523)
Mn	0.15±0.05	0.0005 (0.42)
Zn	0.18±0.09	0.014 (11.2)
total	0.83±0.28	1

[a] Not detectable with microPIXE

[b] Based on literature data from Boehm *et al*. [29] (calcium) and Grant & Pramer [30], normalized to the sum of all 6 metals. The calculated concentrations in 2×YT medium are indicated in μM in parentheses.

Table S9. Conserved active pocket (P) and access tunnel (T) residues for choline sulfatases[a].

residue[b]	part of	α-CS[c]		β-CS[c]	
		residue	%	residue	%
Leu53	P	Leu	100	Leu	100
Tyr123	P/T	Tyr	100	Tyr	100
Trp129	P	Trp	100	Trp	100
Trp142	T	Trp	100	-	100
Trp143	P/T	Trp	100	Trp	100
Tyr144	P/T	Tyr	100	Tyr	100
His145	P/T	His	100	His	100
Gly148	T	Gly	100	Ser/Gly	85/15
Ser149	T	Ser	100	Ser	100
Gly152	T	Gly	100	Asp/Glu	85/15
Ala153	T	Ala/Ser	88/12	Ala	100
Thr159	T	Thr/Ser	65/35	Thr	100
Asp202	P/T	Asp	100	Asp	100
Pro203	T	Pro	100	Pro	100
Val205	T	Val	100	Ala/Val	96/4
Met310	P/T	Met	100	Met	100
Glu386[d]	P	Glu	100	Glu	100
Leu499	T	Leu	100	Leu	100
Leu502	T	Leu/Val	84/16	Leu	100
Arg507	T	Arg	100	Arg	100

[a]Indicated in Green in Fig. S8. [b]Numbering corresponding to *Sm*CS. [c]Phylogenetic division as indicated in Fig. 8. [d]Indicated in red in Fig. S8.

Table S10. Secondary Structure Matching (SSM)[a] pairwise alignment data for *Sm*CS with all AP-superfamily like ASs and PMHs of known structure and with *E. coli* alkaline phosphatase (*Ec*AP) and *Xanthomonas axonopodis* nucleotide phosphodiesterase (*Xa*NPP).

Enzyme	PDB ID	r.m.s.d.[b] (Å)	aligning residues[b]	sequence identity[b] (%)
*Ar*PMH	4UPH	2.04	369 (72%)	29
*Bc*PMH	2W8S	2.00	374 (73%)	28
*Rl*PMH	2VQR	1.98	369 (72%)	**31**
*Sp*PMH	4UPK	2.03	371 (73%)	27
*Sp*AS1	4UPI	2.00	**390 (76%)**	27
*Sp*AS2	4UPL	2.01	382 (75%)	27
*Bf*AS	2QZU	1.83	356 (70%)	25
*Bt*2OS	3B5Q	1.87	369 (72%)	27
*Bt*6OS2	5G2V	1.90	361 (71%)	24
*Ec*AS	3ED4	2.02	330 (65%)	30
PAS	1HDH	2.35	342 (67%)	28
*Hs*ASA	1AUK	2.28	323 (63%)	27
*Hs*ASB	1FSU	2.04	325 (63%)	23
*Hs*ASC	1P49	2.04	329 (64%)	28
*Hs*GalN6S	4FDI	2.14	325 (64%)	25
*Hs*I2S	5FQL	**1.74**	340 (67%)	27
*Hs*SGSH	4MHX	2.08	340 (67%)	26
*Xa*NPP	2GSN	2.14	239 (47%)	20
*Ec*AP	1ED8	3.42	216 (42%)	15

[a]Done at the PDBeFold server (http://www.ebi.ac.uk/msd-srv/ssm/cgi-bin/ssmserver) [5].
[b]Best score indicated in bold.

Table S11. Secondary Structure Matching (SSM)[a] data for the multiple structural alignment data for *Sm*CS with all AP-superfamily-like ASs and PMHs of known structure.

Enzyme	PDB ID	r.m.s.d.[b] (Å)	Δr.m.s.d.[c] (Å)	sequence identity[b] (%)
*Ar*PMH	4UPH	1.98	-0.06	**33**
*Bc*PMH	2W8S	1.78	-0.22	30
*Rl*PMH	2VQR	**1.76**	-0.22	32
*Sp*PMH	4UPK	1.98	-0.05	29
*Sp*AS1	4UPI	1.83	-0.17	32
*Sp*AS2	4UPL	1.92	-0.09	32
*Bf*AS	2QZU	2.14	+0.31	26
*Bt*2OS	3B5Q	2.03	+0.16	27
*Bt*6OS2	5G2V	1.94	+0.04	28
*Ec*AS	3ED4	2.51	+0.49	32
PAS	1HDH	2.56	+0.21	27
*Hs*ASA	1AUK	2.64	+0.36	28
*Hs*ASB	1FSU	2.42	+0.38	23
*Hs*ASC	1P49	2.44	+0.40	29
*Hs*GalN6S	4FDI	2.56	+0.42	24
*Hs*I2S	5FQL	1.94	+0.20	31
*Hs*SGSH	4MHX	2.17	+0.03	26

[a]Done at the PDBeFold server (http://www.ebi.ac.uk/msd-srv/ssm/cgi-bin/ssmserver) [5].

[b]Best score indicated in bold.

[c]Δr.m.s.d = r.m.s.d.(multiple structural alignment) – r.m.s.d.(pairwise structural alignment)

Table S12. (Putative) phosphonate monoester hydrolases (PMHs) included in the alignment on which phylogenetic trees in Fig. 8 and Fig. S12 are based

Acronym	Source	NCBI accession number[a]	Annotation[b]
*Aba*PMH	*Acetobacteraceae bacterium* AT-5844	EHM00979	phosphonate monoester hydrolase
*Aco*PMH	*Amorphus coralli* DSM 19760	WP_018697840	hypothetical protein
***Ak*PMH**	*Advenella kashmirensis* WT001	AFK62654	sulfatase
*Apr*BPMH	alpha proteobacterium BAL199	EDP65618	phosphonate monoester hydrolase
***Ar*PMH**	*Agrobacterium radiobacter* K84	WP_012652905	phosphonate monoester hydrolase
*Avi*PMH	*Agrobacterium vitis* S4	WP_012654598	phosphonate monoester hydrolase
***Bc*PMH**	*Burkholderia caryophili* PG2982	AAC44467	phosphonate monoester hydrolase
*Bta*PMH	*Bartonella tamiae* Th307	EJF95542	hypothetical protein
*Ete*PMH	*Elioraea tepidiphila* DSM 17972	WP_019015433	hypothetical protein
*Hosp*PMH	*Hoeflea* sp. 108	WP_018429825	hypothetical protein
*Kla*PMH	*Kiloniella laminariae* DSM 19542	WP_020593376	hypothetical protein
*Mam*PMH	*Mesorhizobium amorphae* CCNWGS0123	WP_006200068	phosphonate monoester hydrolase
*Mau*PMH	*Mesorhizobium australicum* WSM2073	WP_015315407	phosphonate monoester hydrolase
*Mba*4PMH	marine bacterium 01-004080	ACA21535	putative phosphonate monoester hydrolase
*Mci*PMH	*Mesorhizobium ciceri biovar biserrulae* WSM1271	WP_013529293	phosphonate monoester hydrolase
*Mesp*SPMH	*Mesorhizobium* sp. STM 4661	CCV11144	sulfatase
*Mlo*NPMH	*Mesorhizobium loti* NZP2037	WP_019861583	phosphonate monoester hydrolase
*Mlo*PMH	*Mesorhizobium loti* MAFF303099	BAB50528	phosphonate monoester hydrolase

*Mme*PMH	*Mesorhizobium metallidurans* STM 2683	CCV04987	sulfatase
*Mop*PMH	*Mesorhizobium opportunistum* WSM2075	WP_013892676	phosphonate monoester hydrolase
*Oan*CPMH	*Ochrobactrum anthropi* CTS-325	WP_010659804[c]	sulfatase
*Oan*PMH	*Ochrobactrum anthropi* ATCC 49188	ABS14552	sulfatase
*Oin*LPMH	*Ochrobactrum intermedium* LMG 3301	EEQ96067[d]	phosphonate monoester hydrolase
*Oin*MPMH	*Ochrobactrum intermedium* M86	ELT48946[e]	sulfatase
*Oin*PPMH1	*Oceanibaculum indicum* P24	EKE72976	sulfatase
*Oin*PPMH2	*Oceanibaculum indicum* P24	EKE78819	sulfatase
*Pga*2PMH	*Phaeobacter gallaeciensis* 2.10	WP_014873285	phosphonate monoester hydrolase
*Pga*APMH	*Phaeobacter gallaeciensis* ANG1	WP_019297675	phosphonate monoester hydrolase
*Pga*BPMH	*Phaeobacter gallaeciensis* BS107	WP_014881248	phosphonate monoester hydrolase
*Pgi*PMH	*Polymorphum gilvum* SL003B-26A1	WP_013653825	phosphonate monoester hydrolase
*Psp*FPMH	*Pseudovibrio* sp. FO-BEG1	WP_014286191	phosphonate monoester hydrolase
*Psp*JPMH	*Pseudovibrio* sp. JE062	EEA96525	phosphonate monoester hydrolase
*Rba*KPMH	*Rhodobacteraceae bacterium* KLH11	WP_008758954	phosphonate monoester hydrolase
*Rba*YPMH	*Rhodobacterales bacterium* Y4I	EDZ48240	phosphonate monoester hydrolase
*Rce*PMH	*Roseomonas cervicalis* ATCC 49957	EFH13478	phosphonate monoester hydrolase
*Ret*APMH	*Rhizobium etli* CNPAF512	EGE57255[f]	phosphonate monoester hydrolase protein
*Ret*BPMH	*Rhizobium etli* Brasil 5	WP_010009876	phosphonate monoester hydrolase protein
*Ret*CPMH	*Rhizobium etli* CIAT 652	ACE93199	phosphonate monoester hydrolase
*Ret*PMH	*Rhizobium etli* CFN 42	WP_011427174	phosphonate monoester hydrolase
*Rfr*PMH	*Rhizobium freirei* PRF 81	ENN84210[g]	putative sulfatase (sulfuric ester hydrolase) protein

*Rga*PMH	*Rhizobium gallicum bv. gallicum* R602sp	WP_018448351	phosphonate monoester hydrolase
*Rhsp*4PMH	*Rhizobium* sp. 42MFCr.1	WP_018858910	phosphonate monoester hydrolase
*Rhsp*APMH	*Rhizobium* sp. AP16	WP_007694713	phosphonate monoester hydrolase
*Rhsp*CPMH	*Rhizobium* sp. CF142	WP_007824881	phosphonate monoester hydrolase
*Rhsp*EPMH	*Rhizobium* sp. CCGE 510	WP_007632259[h]	phosphonate monoester hydrolase
*Rhsp*FPMH	*Rhizobium* sp. CF122	WP_007798237	phosphonate monoester hydrolase
*Rhsp*MPMH	*Rhizobium* sp. 2MFCol3.1	WP_018901080	phosphonate monoester hydrolase
*Rhsp*PPMH	*Rhizobium* sp. Pop5	EJZ20888	phosphonate monoester hydrolase
***Rl*PMH**	*Rhizobium leguminosarum bv. viciae* 3841	CAK03956	putative sulfatase
*Rle*PMH2	*Rhizobium leguminosarum bv. viciae* 3841	CAK10140	putative phosphonate monoester hydrolase
*Rle*S5PMH	*Rhizobium leguminosarum bv. trifolii* SRDI565	WP_017966076	phosphonate monoester hydrolase
*Rle*S9PMH	*Rhizobium leguminosarum bv. trifolii* SRDI943	WP_017995530	phosphonate monoester hydrolase
*Rle*T0PMH	*Rhizobium leguminosarum bv. trifolii* WSM2012	WP_003567611	phosphonate monoester hydrolase
*Rle*T1PMH	*Rhizobium leguminosarum bv. trifolii* WSM1325	WP_012759471	phosphonate monoester hydrolase
*Rle*T2PMH	*Rhizobium leguminosarum bv. trifolii* WSM2304	WP_012559338	phosphonate monoester hydrolase
*Rle*T5PMH	*Rhizobium leguminosarum bv. trifolii* WSM597	WP_003589614	phosphonate monoester hydrolase
*Rle*T9PMH	*Rhizobium leguminosarum bv. trifolii* WSM2297	EJC81803	arylsulfatase A family protein
*Rle*V2PMH1	*Rhizobium leguminosarum bv. viciae* 248	WP_020047683	phosphonate monoester hydrolase
*Rle*V2PMH2	*Rhizobium leguminosarum bv. viciae* 248	WP_020052154	phosphonate monoester hydrolase
*Rle*V3PMH	*Rhizobium leguminosarum bv. viciae* 128C53	WP_018244095	phosphonate monoester hydrolase
*Rle*V5PMH	*Rhizobium leguminosarum bv. viciae* WSM1455	WP_003543883	phosphonate monoester hydrolase
*Rle*V8PMH	*Rhizobium leguminosarum bv. viciae* WSM1481	WP_017989817	phosphonate monoester hydrolase

*Rle*VCPMH	*Rhizobium leguminosarum bv. viciae* Vc2	WP_018481475	phosphonate monoester hydrolase
*Rle*VHPMH1	*Rhizobium leguminosarum bv. viciae* Vh3	WP_018496166	phosphonate monoester hydrolase
*Rle*VHPMH2	*Rhizobium leguminosarum bv. viciae* Vh3	WP_018494184	phosphonate monoester hydrolase
*Rle*VTPMH1	*Rhizobium leguminosarum bv. viciae* TOM	WP_017957474	phosphonate monoester hydrolase
*Rle*VTPMH2	*Rhizobium leguminosarum bv. viciae* TOM	WP_017962563	phosphonate monoester hydrolase
*Rma*PMH	*Reyranella massiliensis* 521	WP_020697859	hypothetical protein
*Rme*PMH	*Rhizobium mesoamericanum* STM3625	CCM77813	putative sulfatase/phosphonate monoester hydrolase
*Rnu*PMH	*Roseovarius nubinhibens* ISM	EAP75110	phosphonate monoester hydrolase, putative
*Rosp*BPMH	*Roseomonas* sp. B5	WP_019459218[i]	sulfatase
*Rosp*GPMH	*Roseobacter* sp. GAI101	EEB82878	phosphonate monoester hydrolase
*Rosp*PMH	*Roseobacter* sp. MED193	EAQ43397	phosphonate monoester hydrolase, putative
*Rosp*SPMH	*Roseobacter* sp. SK209-2-6	EBA17301	phosphonate monoester hydrolase, putative
*Rtr*PMH	*Rhizobium tropici* CIAT 899	AGB73001	phosphonate monoester hydrolase
*Rusp*PMH	*Ruegeria* sp. R11	EEB69722	phosphonate monoester hydrolase
*Rusp*TPMH	*Ruegeria* sp. TW15	WP_010443425	phosphonate monoester hydrolase
*Sag*PMH	*Stappia aggregata* IAM 12614	WP_006936552	phosphonate monoester hydrolase
*Sal*PMH	*Labrenzia alexandrii* DFL-11	WP_008196700	phosphonate monoester hydrolase
*Sla*PMH	*Silicibacter lacuscaerulensis* ITI-1157	EEX11451	phosphonate monoester hydrolase

***Sp*PMH**	*Silicibacter pomeroyi* DSS-3	AAV97522	phosphonate monoester hydrolase, putative
*Tsp*PMH	*Thalassiobium* sp. R2A62	WP_009159518[j]	phosphonate monoester hydrolase
*Uba*3PMH	uncultured alpha proteobacterium 01-003886	ACB13617[k]	putative phosphonate monoester hydrolase
*Vap*PMH	*Verminephrobacter aporrectodeae* subsp. *tuberculatae* At4	WP_010104960	sulfatase
*Vei*PMH	*Verminephrobacter eiseniae* EF01-2	ABM57990	sulfatase

[a]For the NCBI protein database

[b]Putative function associated with the accession number listed here

[c]Correct reading frame starts 258 bp upstream (=+86 aa at N-terminus) of the coding sequence associated with accession number

[d]Correct reading frame starts 66 bp upstream (=+22 aa at N-terminus) of the coding sequence associated with accession number

[e]Correct reading frame starts 210 bp upstream (=+70 aa at N-terminus) of the coding sequence associated with accession number

[f]Correct reading frame starts 39 bp downstream (=-13 aa at N-terminus) of the coding sequence associated with accession number

[g]Correct reading frame starts 126 bp upstream (=+42 aa at N-terminus) of the coding sequence associated with accession number

[h]Correct reading frame starts 117 bp upstream (=+39 aa at N-terminus) of the coding sequence associated with accession number

[i]Correct reading frame starts 57 bp upstream (=+19 aa at N-terminus) of the coding sequence associated with accession number

[j]Correct reading frame starts 57 bp downstream (=-19 aa at N-terminus) of the coding sequence associated with accession number

[k]Correct reading frame starts 33 bp downstream (=-11 aa at N-terminus) of the coding sequence associated with accession number

Table S13. (Putative) arylsulfatases (ASs) included in the alignment on which phylogenetic trees in Fig. 8 and Fig. S13 are based.

Acronym	Source	NCBI accession number[a]	Annotation[b]
*Aco*AS	*Amorphus coralli* DSM 19760	WP_018699465	hypothetical protein
*Afa*AS	*Alcaligenes faecalis* subsp. *faecalis* NCIB 8687	EJC65132	sulfatase family protein 5
***Ak*AS**	*Advenella kashmirensis* WT001	ZP_09480898	sulfatase
*Alsp*AS1	*Alcaligenes* sp. HPC1271	EKU28206	sulfatase family protein 5
*Alsp*AS2	*Alcaligenes* sp. HPC1271	EKU28248	sulfatase
*Api*HAS	*Achromobacter piechaudii* HLE	EJO30007	sulfatase family protein 10
*Api*AS	*Achromobacter piechaudii* ATCC 43553	EFF74148	arylsulfatase
*Apr*AAS	alpha proteobacterium SCGC AAA076C03	WP_020056865[c]	phosphonate monoester hydrolase
*Apr*AS	*Rhodobacterales bacterium* HTCC2255	EAU52967[c]	sulfatase family protein
*Asp*RAS	*Ahrensia* sp. R2A130	EFL87619	sulfatase
*Axy*AAS	*Achromobacter xylosoxidans* AXX-A	EGP48313	sulfatase family protein 10
*Axy*CAS	*Achromobacter xylosoxidans* C54	EFV84062	sulfatase
*Axy*NAS	*Achromobacter xylosoxidans* NH447841996	CCH10057	putative sulfatase
*Axy*AS1	*Achromobacter xylosoxidans* A8	ADP17527	sulfatase family protein 9
*Axy*AS2	*Achromobacter xylosoxidans* A8	ADP17154	sulfatase family protein 8
*Axy*AS3	*Achromobacter xylosoxidans* A8	ADP16373	sulfatase family protein 5
*Axy*AS4	*Achromobacter xylosoxidans* A8	ADP19340[d]	sulfatase family protein 13
AxyAS5	*Achromobacter xylosoxidans* A8	ADP17824	sulfatase family protein 10
*Bbr*2AS	*Bordetella bronchiseptica* 253	CCJ53644	putative sulfatase
*Bbr*9AS	*Bordetella bronchiseptica* 1289	CCN22493	putative sulfatase

*Bbr*BAS	*Bordetella bronchiseptica* Bbr77	CCN05088	putative sulfatase
*Bbr*DAS	*Bordetella bronchiseptica* D445	CCN19505	putative sulfatase
*Bbr*AS	*Bordetella bronchiseptica* RB50	CAE32285	putative sulfatase
*Bja*4AS	*Bradyrhizobium japonicum* USDA 124	WP_018643868	phosphonate monoester hydrolase
*Bja*6AS	*Bradyrhizobium japonicum* USDA 6	BAL05689	phosphonate monoester hydrolase
*Bja*AS	*Bradyrhizobium japonicum* USDA 110	WP_011083258	phosphonate monoester hydrolase
*Bja*WAS	*Bradyrhizobium japonicum* WSM2793	WP_018318842	phosphonate monoester hydrolase
*Bosp*AS1	*Bordetella* sp. FB8	WP_019937074	phosphonate monoester hydrolase
*Bosp*AS2	*Bordetella* sp. FB8	WP_019937234	hypothetical protein
*Bpa*BAS	*Bordetella parapertussis* Bpp5	CCJ48824	putative sulfatase
*Bpa*AS	*Bordetella parapertussis* 12822	CAE37634	putative sulfatase
*Bpe*AS	*Bordetella petrii* DSM 12804	CAP43760	putative sulfatase
*Bph*AS	*Burkholderia phytofirmans* PsJN	ACD16460	sulfatase
*Bsp*2AS	*Bradyrhizobium* sp. WSM1253	EIG60463	arylsulfatase A family protein
*Bsp*SAS	*Bradyrhizobium* sp. S23321	BAL73642	putative phosphonate monoester hydrolase
*Bsp*WAS	*Bradyrhizobium* sp. WSM471	EHQ99909	arylsulfatase A family protein
*Bsp*YAS	*Bradyrhizobium* sp. YR681	EJN11942	arylsulfatase A family protein
*Cba*BAS	*Cupriavidus basilensis* B-8	WP_017228287	phosphonate monoester hydrolase
*Cba*OAS	*Cupriavidus basilensis* OR16	EHP44391	sulfatase
*Cne*HAS	*Cupriavidus necator* HPC(L)	EKN55318	sulfatase family protein
*Cne*AS	*Cupriavidus necator* N-1	AEI80490	sulfatase
*Cta*AS	*Cupriavidus taiwanensis* LMG 19424	WP_012356146	phosphonate monoester hydrolase

*Cta*SAS	*Cupriavidus taiwanensis* STM 6070	WP_018007253	phosphonate monoester hydrolase
*Cusp*HAS	*Cupriavidus* sp. HMR1	EKZ95862	sulfatase
*Cusp*UAS	*Cupriavidus* sp. UYPR2.512	WP_018310934	phosphonate monoester hydrolase
*Cusp*WAS	*Cupriavidus* sp. WS	WP_020205660	phosphonate monoester hydrolase
*Jasp*AS	*Jannaschia* sp. CCS1	ABD54485	sulfatase
*Lve*AS	*Loktanella vestfoldensis* SKA53	EAQ05466	sulfatase family protein
*Mesp*AS	*Mesorhizobium* sp. WSM4349	WP_018459858	phosphonate monoester hydrolase
*Oant*2AS	*Octadecabacter antarcticus* 238	AGI74443	sulfatase
*Oant*AS	*Octadecabacter antarcticus* 307	AGI66430	sulfatase, putative
*Ocsp*AS1	*Oceanicola* sp. S124	WP_010139804	phosphonate monoester hydrolase
*Ocsp*AS2	*Oceanicola* sp. S124	WP_010139987	phosphonate monoester hydrolase
*Ogu*AS1	*Oceaniovalibus guishaninsula* JLT2003	EKE43222[e]	sulfatase family protein
*Ogu*AS2	*Oceaniovalibus guishaninsula* JLT2003	EKE45655	putative sulfatase
*Oin*AS	*Oceanibulbus indolifex* HEL-45	EDQ05822[c]	sulfatase family protein
*Pasp*AS	*Pandoraea* sp. B6	WP_017235511	hypothetical protein
*Pga*2AS	*Phaeobacter gallaeciensis* 2.10	AFO86385	sulfatase
*Pga*AAS	*Phaeobacter gallaeciensis* ANG1	WP_019297020	phosphonate monoester hydrolase
*Pga*BAS	*Phaeobacter gallaeciensis* BS107	AFO90144	sulfatase
*Pin*AS	*Pseudacidovorax intermedius* NH-1	WP_017760293	hypothetical protein
*Psp*FAS	*Pseudovibrio* sp. FO-BEG1	AEV39842	sulfatase family protein
*Pusp*AS	*Pusillimonas* sp. T7-7	AEC22137	putative sulfatase
*Rasp*AS	*Ralstonia* sp. GA33	EON16278	sulfatase
*Rba*8AS	*Rhodobacteraceae bacterium* HTCC2083	EDZ44044	sulfatase family protein

*Rba*CAS1	*Rhodobacterales bacterium* HTCC2150	EBA02327	sulfatase family protein
*Rba*CAS2	*Rhodobacterales bacterium* HTCC2150	EBA02400	putative sulfatase
*Rba*CAS3	*Rhodobacterales bacterium* HTCC2150	EBA02113	putative phosphonate monoester hydrolase
*Rba*HAS1	*Maritimibacter alkaliphilus* HTCC2654	EAQ14969	sulfatase family protein
*Rba*HAS2	*Maritimibacter alkaliphilus* HTCC2654	EAQ13871[f]	sulfatase family protein
*Rba*KAS	*Rhodobacteraceae bacterium* KLH11	EEE35532	sulfatase family protein
*Rba*YAS	*Rhodobacterales bacterium* Y4I	EDZ46738	sulfatase
*Rde*AS	*Roseobacter denitrificans* OCh 114	ABG33120	sulfatase family protein
*Reu*HAS	*Ralstonia eutropha* H16	CAJ96315	sulfatase
*Rli*AS	*Roseobacter litoralis* Och 149	AEI92845	putative sulfatase
*Rma*AS	*Reyranella massiliensis* 521	WP_020698612	hypothetical protein
***Rm*AS**	*Cupriavidus metallidurans* CH34	ABF08681	sulfatase
*Rmo*AS	*Ruegeria mobilis* F1926	ENZ89560	sulfatase
*Rosp*AS	*Roseobacter* sp. MED193	EAQ46993	putative sulfatase
***Rp*AS**	*Rhodopseudomonas palustris* CGA009	CAE26808	putative sulfatase
*Rpa*TAS	*Rhodopseudomonas palustris* TIE-1	ACF00106	sulfatase
*Rsp*TAS	*Roseibium* sp. TrichSKD4	EFO29812	sulfatase
*Rusp*AS	*Ruegeria* sp. R11	EEB72807	sulfatase
*Rusp*TAS	*Ruegeria* sp. TW15	WP_010441354[g]	phosphonate monoester hydrolase
***Sa*AS**	*Stappia aggregata* IAM 12614	EAV45217	sulfatase family protein
*Sisp*AS	*Silicibacter* sp. TM1040	ABF64845[h]	sulfatase
*Sla*AS	*Silicibacter lacuscaerulensis* ITI-1157	EEX11418	sulfatase family protein

***Sp*AS1**	*Silicibacter pomeroyi* DSS-3	AAV97258	sulfatase family protein
***Sp*AS2**	*Silicibacter pomeroyi* DSS-3	AAV96818	sulfatase family protein
*Ssp*TAS	*Silicibacter* sp. TrichCH4B	EEW59682	sulfatase
*Sst*AS	*Sagittula stellata* E-37	EBA10122	sulfatase family protein
*Susp*AS	*Sulfitobacter* sp. EE-36	EAP83576[c]	putative sulfatase
*Thsp*AS1	*Thiomonas* sp. FB6	WP_018912210	hypothetical protein
*Thsp*AS2	*Thiomonas* sp. FB6	WP_018914950	hypothetical protein
*Tsp*AS	*Thalassiobium* sp. R2A62	EET46687	sulfatase

[a]For the NCBI protein database

[b]Putative function associated with the accession number listed here

[c]Correct reading frame starts 24 bp upstream (=+8 aa at N-terminus) of the coding sequence associated with accession number

[d]Correct reading frame starts 24 bp downstream (=-8 aa at N-terminus) of the coding sequence associated with accession number

[e]Correct reading frame starts 60 bp downstream (=-20 aa at N-terminus) of the coding sequence associated with accession number

[f]Correct reading frame starts 30 bp upstream (=+10 aa at N-terminus) of the coding sequence associated with accession number

[g]Correct reading frame starts 171 bp upstream (=+57 aa at N-terminus) of the coding sequence associated with accession number

[h]Correct reading frame starts 90 bp downstream (=-30 aa at N-terminus) of the coding sequence associated with accession number

Table S14. (Putative) choline sulfatases included in the alignment on which phylogenetic trees in Fig. 8 and Fig. S14 are based.

Acronym	Source	NCBI accession number[a]	Annotation[b]
*Aal*CS	*Agrobacterium albertimagni* AOL15	EKF58369	choline sulfatase
*Aco*CS	*Amorphus coralli* DSM 19760	WP_040400895[c]	choline sulfatase
*Afa*CS	*Agrobacterium fabrum* str. C58	AAK88799	choline sulfatase
*Amu*CS	*Acidiphilium multivorum* AIU301	BAJ80229	choline sulfatase
*Ansp*CS	*Ancylobacter* sp. FA202	WP_018388827	choline sulfatase
*Apr*BCS	alpha proteobacterium BAL199	EDP64145	choline sulfatase
*Apr*CS	alpha proteobacterium HTCC2255	EAU52513	choline sulfatase
*Apr*JCS	alpha proteobacterium SCGC AAA300J04	WP_019977153	choline sulfatase
*Ara*CS	*Agrobacterium radiobacter* K84	ACM25228	choline sulfatase
*Aur*CS	*Aureimonas ureilytica* DSM 18598	WP_019996780	choline sulfatase
*Bam*MCS	*Burkholderia ambifaria* MC40-6	ACB67455	choline sulfatase
*Bce*CS	*Burkholderia cepacia* GG4	AFQ49765	choline sulfatase
*Bcen*JCS	*Burkholderia cenocepacia* J2315	CAR56172	putative choline sulfatase
*Bcen*MCS	*Burkholderia cenocepacia* MC0-3	ACA94342	choline sulfatase
*Bcen*PCS	*Burkholderia cenocepacia* PC184	EAY66500[d]	arylsulfatase A
*Bdo*CS	*Burkholderia dolosa* AUO158	WP_006766372	choline sulfatase
*Bgl*CS	*Burkholderia gladioli* BSR3	WP_013689655	choline sulfatase
*Bgr*CS	*Burkholderia graminis* C4D1M	EDT07622	choline sulfatase
*Bmu*CCS	*Burkholderia multivorans* CGD1	EEE02548	choline sulfatase
*Bph*BCS	*Burkholderia phenoliruptrix* BR3459a	AFT89806	choline sulfatase

*Bph*CS	*Burkholderia phytofirmans* PsJN	ACD19439	choline sulfatase
*Bphy*CS	*Burkholderia phymatum* STM815	ACC72446	choline sulfatase
*Bps*ACS	*Burkholderia pseudomallei* 1106a	ABN94520	choline sulfatase
*Bpy*CS	*Burkholderia pyrrocinia* CH-67	WP_017328634	choline sulfatase
*Bth*ECS	*Burkholderia thailandensis* E264	ABC34076	choline sulfatase
*Bub*CS	*Burkholderia ubonensis* Bu	WP_010089893	choline sulfatase
*Busp*3CS	*Burkholderia* sp. 383	WP_011354195	choline sulfatase
*Busp*BCS	*Burkholderia* sp. BT03	EUC19381	choline sulfatase
*Busp*CCS	*Burkholderia* sp. Ch11	EIF33895	choline sulfatase
*Busp*ECS	*Burkholderia* sp. CCGE1003	ADN61799	choline sulfatase
*Busp*JCS	*Burkholderia* sp. SJ98	EKS67237	choline sulfatase
*Busp*YCS	*Burkholderia* sp. JPY347	WP_018438864	choline sulfatase
*Bvi*CS	*Burkholderia vietnamiensis* G4	WP_011880763	choline sulfatase
*Cfu*CS	*Collimonas fungivorans* Ter331	AEK61239	choline sulfatase
*Cisp*3CS	*Citreicella* sp. 357	EIE49541	choline sulfatase
*Cisp*CS	*Citreicella* sp. SE45	EEX15190	choline sulfatase
*Fpe*CS	*Fulvimarina pelagi* HTCC2506	EAU43046	choline sulfatase
*Hosp*CS	*Hoeflea* sp. 108	WP_018426936	choline sulfatase
*Jasp*CS	*Jannaschia* sp. CCS1	WP_011454618	choline sulfatase
*Kla*CS	*Kiloniella laminariae* DSM 19542	WP_020593088	choline sulfatase
*Lho*CS	*Loktanella hongkongensis* DSM 17492	WP_017927100	choline sulfatase
*Lor*CS	*Leeia oryzae* DSM 17879	WP_018150207	choline sulfatase
*Lve*CS	*Loktanella vestfoldensis* SKA53	EAQ07054	choline sulfatase

*Mal*CS	*Mesorhizobium alhagi* CCNWXJ122	EHK53565	choline sulfatase
*Mci*CS	*Mesorhizobium ciceri biovar biserrulae* WSM1271	ADV14026	choline sulfatase
*Mlo*CS	*Mesorhizobium loti* MAFF303099	BAB54039	choline sulfatase
*Mme*7CS	*Martelella mediterranea* DSM 17316	WP_018065286	choline sulfatase
*Oant*2CS	*Octadecabacter antarcticus* 238	WP_015494050	choline sulfatase
*Oba*CS	*Oceanicola batsensis* HTCC2597	EAQ04277	choline sulfatase
*Ogr*CS	*Oceanicola granulosus* HTCC2516	EAR49742	choline sulfatase
*Oin*MCS	*Ochrobactrum intermedium* M86	ELT47968	choline sulfatase
*Pasp*TCS	*Paracoccus* sp. TRP	WP_010400371	choline sulfatase
*Pfe*CS	*Pseudogulbenkiania ferrooxidans* 2002	EEG08674	choline sulfatase
*Phsp*CS	*Phyllobacterium* sp. YR531	EJN02641	choline sulfatase
*Psa*CS	*Pseudaminobacter salicylatoxidans* KCT001	WP_019172128	choline sulfatase
*Psp*FCS	*Pseudovibrio* sp. FO-BEG1	WP_014284790	choline sulfatase
*Pssp*CS	*Pseudogulbenkiania* sp. NH8B	BAK76882	choline sulfatase
*Rba*8CS	*Rhodobacterales bacterium* HTCC2083	EDZ41682	choline sulfatase
*Rba*HCS	*Rhodobacterales bacterium* HTCC2654	EAQ12274	choline sulfatase
*Rba*KCS	*Rhodobacteraceae bacterium* KLH11	EEE37986	choline sulfatase
*Rca*CS	*Rhodobacter capsulatus* SB 1003	ADE87020	choline sulfatase
*Rgi*CS	*Rhizobium giardinii bv. giardinii* H152	WP_018329443	choline sulfatase
*Rhod*CS	*Rhodobacter* sp. SW2	EEW26103	choline sulfatase
*Rhsa*CS	*Rhodobacter sphaeroides* ATCC 17025	WP_011907552	choline sulfatase
*Rhsp*8CS	*Rhizobium* sp. CF080	EUB95417	choline sulfatase
*Rhsp*CCS	*Rhizobium* sp. CF142	EJJ27032	choline sulfatase

*Rhsp*DCS	*Rhizobium* sp. PDO1076	EHS48732	choline sulfatase
*Rhsp*MCS	*Rhizobium* sp. 2MFCol3.1	WP_018898138	choline sulfatase
*Rle*CS	*Rhizobium leguminosarum bv. viciae* 3841	WP_011655165	choline sulfatase
*Rli*CS	*Roseobacter litoralis* Och 149	WP_013961109	choline sulfatase
*Rme*CS	*Rhizobium mesoamericanum* STM3625	CCM74096	choline sulfatase
*Rmo*CS	*Ruegeria mobilis* F1926	ENZ91036	choline sulfatase
*Rnu*CS	*Roseovarius nubinhibens* ISM	EAP76557	choline sulfatase
*Rosp*ACS	*Roseobacter* sp. AzwK-3b	WP_007816210	choline sulfatase
*Rosp*CCS	*Roseobacter* sp. CCS2	EBA12538	choline-sulfatase
*Rosp*GCS	*Roseobacter* sp. GAI101	EEB82971	choline-sulfatase
*Rosp*SCS	*Roseobacter* sp. SK209-2-6	EBA16545	choline sulfatase
*Rtr*CS	*Rhizobium tropici* CIAT 899	AGB69832	choline sulfatase
*Sag*CS	*Stappia aggregata* IAM 12614	EAV42108	choline sulfatase
***Sm*CS**	*Sinorhizobium meliloti* 1021	O69787	choline sulfatase
*Smu*CS	*Salipiger mucosus* DSM 16094	EPX76305	choline sulfatase
*Sno*CS	*Starkeya novella* DSM 506	ADH87472	choline sulfatase
*Spo*CS	*Silicibacter pomeroyi* DSS-3	AAV94383	choline sulfatase
*Tpr*CS	*Thalassospira profundimaris* WP0211	EKF09353	choline sulfatase
*Tsp*CS	*Thalassiobium* sp. R2A62	EET48863	choline sulfatase
*Txi*CS	*Thalassospira xiamenensis* M5 = DSM 17429	AJD53430	choline sulfatase
*Wma*CS	*Wenxinia marina* DSM 24838	WP_018303808	choline sulfatase

[a]For the NCBI protein database

[b]Putative function listed in the NCBI database

[c]Correct reading frame starts 30 bp upstream (=+10 aa at N-terminus) of the coding sequence associated with accession number.

[d]Correct reading frame starts 102 bp downstream (=-34 aa at N-terminus) of the coding sequence associated with accession number.

Table S15. Primers used for cloning and site-directed mutagenesis

Function	Mutation	Primers		Restriction site
Cloning	n.a	forward	5'-gcgcgcggatcccGTGACCACCGGTAAGCCCAACATT-3'	*Bam*HI
	n.a	reverse	5’-cgcgcgaagcttTCATTCTCCTCGCGGATAGCGTTTG-3’	*Hind*III
Mutagenesis	C54A	forward	5’-ACCTCGTCGCCCCTG**gcT**GCCCCTGCCCGCGCA-3’	n.a.
		reverse	5’-TGCGCGGGCAGGGGC**Agc**CAGGGGCGACGAGGT-3'	n.a.
	C54S	forward	5’-ACCTCGTCGCCCCTG**tca**GCCCCTGCCCGCGCA-3’	n.a.
		reverse	5’-TGCGCGGGCAGGGGC**tga**CAGGGGCGACGAGGT-3’	n.a.
	K102L	forward	5’-ACCGCGCTTTCCGGC**ctG**ATGCATTTCGTCGGG-3’	n.a.
		reverse	5’-CCCGACGAAATGCAT**Cag**GCCGGAAAGCGCGGT-3’	n.a.
	H104A	forward	5’-CTTTCCGGCAAGATG**gcT**TTCGTCGGGCCGGAC-3’	n.a.
		reverse	5’-GTCCGGCCCGACGAA**Agc**CATCTTGCCGGAAAG-3’	n.a.
	H201A	forward	5’-TCCTTCACCCACCCG**gcC**GACCCCTATGTCGCG-3’	n.a.
		reverse	5’-CGCGACATAGGGGTC**Ggc**CGGGTGGGTGAAGGA-3’	n.a.
	K309L	forward	5’-CGCGGCCTCTGGTTC**ctG**ATGAACTTCTTCGAA-3’	n.a.
		reverse	5’-TTCGAAGAAGTTCAT**Cag**GAACCAGAGGCCGCG-3’	n.a.
	E386L	forward	5'-ATGGAATACGCGGCT**ctG**GCCTCCTATGCACCG-3'	n.a.
		reverse	5'-CGGTGCATAGGAGGC**Cag**AGCCGCGTATTCCAT-3'	n.a.
	E490stop	forward	5'-CTGCAAAAGGCATCG**tga**CGCTACATGCGCAAC-3'	n.a.
	(Δ23)	reverse	5'-GTTGCGCATGTAGCG**tca**CGATGCCTTTTGCAG-3'	n.a.
	T501stop	forward	5'-CACATGAACCTCGAC**tga**CTCGAGGAATCCAAA-3'	n.a.
	(Δ12)	reverse	5'-TTTGGATTCCTCGAG**tca**GTCGAGGTTCATGTG-3'	n.a.

Supporting references

[1] Edwards, D. R., Lohman, D. C. & Wolfenden, R. Catalytic proficiency: the extreme case of S-O cleaving sulfatases. J Am Chem Soc 2012;134:525-31.

[2] Dawson, R. M. C. (1959). *Data for Biochemical Research*, Clarendon Press, Oxford.

[3] Sánchez-Romero, J. J. & Olguin, L. F. Choline sulfatase from *Ensifer* (*Sinorhizobium*) *meliloti*: Characterization of the unmodified enzyme. Biochem Biophys Rep 2015;3:161-8.

[4] Osteras, M., Boncompagni, E., Vincent, N., Poggi, M. C. & Le Rudulier, D. Presence of a gene encoding choline sulfatase in *Sinorhizobium meliloti bet* operon: choline-*O*-sulfate is metabolized into glycine betaine. Proc Natl Acad Sci U S A 1998;95:11394-9.

[5] Krissinel, E. & Henrick, K. Secondary-structure matching (SSM), a new tool for fast protein structure alignment in three dimensions. Acta Crystallogr D Biol Crystallogr 2004;60:2256-68.

[6] Galperin, M. Y., Bairoch, A. & Koonin, E. V. A superfamily of metalloenzymes unifies phosphopentomutase and cofactor-independent phosphoglycerate mutase with alkaline phosphatases and sulfatases. Protein Sci 1998;7:1829-35.

[7] Galperin, M. Y. & Jedrzejas, M. J. Conserved core structure and active site residues in alkaline phosphatase superfamily enzymes. Proteins 2001;45:318-24.

[8] Jonas, S. & Hollfelder, F. Mapping Catalytic Promiscuity in the Alkaline Phosphatase Superfamily. Pure Appl Chem 2009;81:731-42.

[9] van Loo, B., Bayer, C. D., Jonas, S., Fisher, G., Valkov, E., Mohammed, M. F., Vorobieva, A., Dutruel, C., Hyvonen, M. & Hollfelder, F. Balancing Specificity and Promiscuity in Enzyme Evolution: Multidimensional Activity Transitions in the Alkaline Phosphatase Superfamily. submitted 2018;

[10] Jonas, S., van Loo, B., Hyvonen, M. & Hollfelder, F. A new member of the alkaline phosphatase superfamily with a formylglycine nucleophile: structural and kinetic characterisation of a phosphonate monoester hydrolase/phosphodiesterase from *Rhizobium leguminosarum*. J Mol Biol 2008;384:120-36.

[11] van Loo, B., Jonas, S., Babtie, A. C., Benjdia, A., Berteau, O., Hyvonen, M. & Hollfelder, F. An efficient, multiply promiscuous hydrolase in the alkaline phosphatase superfamily. Proc Natl Acad Sci U S A 2010;107:2740-5.

[12] Kirby, A. J. & Vargolis, A. G. The Reactivity of Phosphate Esters. Monoester Hydrolysis. J Am Chem Soc 1967;89:415-23.

[13] van Loo, B., Berry, R., Dokphrom, U., Golicnik, M., Hengge, A. C. & Hollfelder, F. *Pseudomonas aeruginosa* Arylsulfatase Sulfatase and Phosphatase Reactions: Comparative Mechanistic Analysis. Manuscript in prep 2018;

[14] Kirby, A. J. & Jencks, W. P. The reactivity of nucleophilic reagents toward the *p*-nitrophenyl phosphate dianion. J Am Chem Soc 1965;87:3209-16.

[15] Kirby, A. J. & Younas, M. The Reactivity of Phosphate Esters. Reactions of Diesters with Nucleophiles. J Chem Soc B 1970;1165-72.

[16] Zalatan, J. G. & Herschlag, D. Alkaline phosphatase mono- and diesterase reactions: comparative transition state analysis. J Am Chem Soc 2006;128:1293-303.

[17] Purcell, J. & Hengge, A. C. The thermodynamics of phosphate versus phosphorothioate ester hydrolysis. J Org Chem 2005;70:8437-42.

[18] McWhirter, C., Lund, E. A., Tanifum, E. A., Feng, G., Sheikh, Q. I., Hengge, A. C. & Williams, N. H. Mechanistic study of protein phosphatase-1 (PP1), a catalytically promiscuous enzyme. J Am Chem Soc 2008;130:13673-82.

[19] Krissinel, E. & Henrick, K. Inference of macromolecular assemblies from crystalline state. J Mol Biol 2007;372:774-97.

[20] Ulmer, J. E., Vilen, E. M., Namburi, R. B., Benjdia, A., Beneteau, J., Malleron, A., Bonnaffe, D., Driguez, P. A., Descroix, K., Lassalle, G., Le Narvor, C., Sandstrom, C., Spillmann, D. & Berteau, O. Characterization of glycosaminoglycan (GAG) sulfatases from the human gut symbiont *Bacteroides thetaiotaomicron* reveals the first GAG-specific bacterial endosulfatase. J Biol Chem 2014;289:24289-303.

[21] Boltes, I., Czapinska, H., Kahnert, A., von Bulow, R., Dierks, T., Schmidt, B., von Figura, K., Kertesz, M. A. & Uson, I. 1.3 A structure of arylsulfatase from *Pseudomonas aeruginosa* establishes the catalytic mechanism of sulfate ester cleavage in the sulfatase family. Structure 2001;9:483-91.

[22] Lukatela, G., Krauss, N., Theis, K., Selmer, T., Gieselmann, V., von Figura, K. & Saenger, W. Crystal structure of human arylsulfatase A: the aldehyde function and the metal ion at the active site suggest a novel mechanism for sulfate ester hydrolysis. Biochemistry 1998;37:3654-364.

[23] Bond, C. S., Clements, P. R., Ashby, S. J., Collyer, C. A., Harrop, S. J., Hopwood, J. J. & Guss, J. M. Structure of a human lysosomal sulfatase. Structure 1997;5:277-89.

[24] Hernandez-Guzman, F. G., Higashiyama, T., Pangborn, W., Osawa, Y. & Ghosh, D. Structure of human estrone sulfatase suggests functional roles of membrane association. J Biol Chem 2003;278:22989-97.

[25] Rivera-Colon, Y., Schutsky, E. K., Kita, A. Z. & Garman, S. C. The structure of human GALNS reveals the molecular basis for mucopolysaccharidosis IV A. J Mol Biol 2012;423:736-51.

[26] Demydchuk, M., Hill, C. H., Zhou, A., Bunkoczi, G., Stein, P. E., Marchesan, D., Deane, J. E. & Read, R. J. Insights into Hunter syndrome from the structure of iduronate-2-sulfatase. Nat Commun 2017;8:15786.

[27] Sidhu, N. S., Schreiber, K., Propper, K., Becker, S., Uson, I., Sheldrick, G. M., Gartner, J., Kratzner, R. & Steinfeld, R. Structure of sulfamidase provides insight into the molecular pathology of mucopolysaccharidosis IIIA. Acta Crystallogr D Biol Crystallogr 2014;70:1321-35.

[28] Hanson, S. R., Best, M. D. & Wong, C. H. Sulfatases: structure, mechanism, biological activity, inhibition, and synthetic utility. Angew Chem Int Ed 2004;43:5736-63.

[29] Boehm, D. F., Welch, R. A. & Snyder, I. S. Calcium is required for binding of *Escherichia coli* hemolysin (HlyA) to erythrocyte membranes. Infect Immun 1990;58:1951-8.

[30] Grant, C. L. & Pramer, D. Minor element composition of yeast extract. J Bacteriol 1962;84:869-70.
